# Supplementary material for: An Integrated Analysis of Mechanistic Insights into Biomolecular Interactions and Molecular Dynamics of Bio-Inspired Cu(II) and Zn(II) Complexes towards DNA/BSA/SARS-CoV-2 3CLpro by Molecular Docking-Based Virtual Screening and FRET Detection
Source: Biomolecules. 2022 Dec 15;12(12):1883. doi: 10.3390/biom12121883 (PMC9775322; doi:10.3390/biom12121883)
Supplement: Supplementary file 1 [file biomolecules-12-01883-s001.zip › biomolecules-2057922-supplementary.pdf]

<u>**Electronic Supplementary Information File**</u>

An integrated analysis of mechanistic insights into biomolecular interactions and molecular dynamics of bio-inspired Cu(II) and Zn(II) complexes towards DNA/BSA/SARS-CoV-2 3CL^{pro} by molecular docking-based virtual screening and FRET detection

Karunganathan Sakthikumar [1], Bienfait Kabuyaya Isamura [1, 3] and Rui Werner Maçedo Krause [1, 2] *

[1] *Department of Chemistry, Center for Chemico- and Biomedicinal Research (CCBR), Faculty of Science, Rhodes University, Grahamstown 6140, Eastern Cape, South Africa*

[2] *Center for Chemico- and Biomedicinal Research (CCBR), Faculty of Science, Rhodes University, Grahamstown 6140, Eastern Cape, South Africa*

[3] *Department of Chemistry, The University of Manchester, Manchester M13 9PL, Great Britain*

* *Correspondence: r.krause@ru.ac.za*

* *Correspondence should be addressed to Rui Werner Maçedo Krause:*

Prof. Rui WM Krause,
Professor, Organic & Medicinal Chemistry,
Department of Chemistry, Faculty of Science, Rhodes University, Grahamstown 6140,
Eastern Cape, South Africa.
Email: r.krause@ru.ac.za,
Phone: +27 741622674; +27 46 603 7030

2a. Experimental section

2.1. Resources and Techniques

We purchased the essential chemical substances and other reagents from Sigma Aldrich repository. Numerous analytical and spectral measurements were utilized to describe all the test compounds, including Bruker 300 MHz ^{1}H NMR spectrometer, or Avance III HD Nanobay 400 MHz, Shimadzu FT-IR spectrometer, Shimadzu UV-Vis 1800, Varian E112 EPR spectrometer, Bruker Daltonics ESI-MS 3000 or Bruker Compact qTOFMS coupled to an LC system and (Varian Inc, USA, 410 proStar binary LC with 500 MS, IT, and PDA detectors, or Thermo Ultimate 3000 with PDA), Bruker AXS KAPPA APEX-2 or D8 ADVANCE diffractometers (SCXRD) and other analytical techniques. The further details were encapsulated in our previous reports [4, 18].

2.2. DNA/BSA interaction studies

2.2.1. DNA cleavage property

The experiment was executed for all test compounds with DNA in the presence of hydrogen peroxide in Tris-HCl buffer solution (pH=7.2) under the aerobic conditions at 35 °C for two hours. The samples (Cocktail mixture) consisting of 15 µL CT-DNA (30 µM), 5 µL test sample (500 µM), 29 µL buffer solution (50 mM TrisHCl & 18 mM NaCl; pH = 7.2) and 1 µL H_2O_2 (500 µM) were shaken well and incubated at 25 °C for two hrs. Following incubation, 1 mL of a 0.25 percentage bromophenol blue dye and a 40 percentage sucrose solution was added to the cocktail mixture. Injections of the resultant mixture were made into chamber wells of 1% agarose gel. A layer of 1 percentage agarose gel was made by dissolving 1g of agarose gel in 98 mL of distilled water and 2 mL of 50x stock solution. Then, ethidium bromide (**EB**) (0.5 µg/mL) was blended to it and heated at 100° C. A 1 percentage agarose gel layer was produced by forming chamber wells after the resultant

solution had been cooled at 25 °C. By soaking the gel in the 2 percentage tank buffer solution, the gel became stained. 20 mL of 50x stock solution containing 2.4228 g Tris base (1M), 8.5 mL ethylenediamineteraacetic acid (EDTA) (0.1 M) solution and 11.42 mL of glacial acetic acid were used to create the 2 percentage tank solution. The CT-DNA was observed to migrate towards the positive pole when 50 V of electric current was applied to the tank buffer solution. The electric current is turned off during the end of electrophoresis. After the completion of the experiment, the gel layer was taken out of the tank solution and exposed to a UV transilluminator. All band lanes were evaluated and compared to the control (DNA+H_2O_2) using bromophenol blue, which acts as a photosensitizer and is useful for visually analyzing the migrated band lanes for the degree of DNA cleavage [19–20].

2.2.2. Evaluation of DNA-binding characteristics

By increasing the concentration of DNA (0–50 μM), electronic absorption titrations were performed at the fixed concentration (50 μM) of all test compounds in aqueous media at room temperature. The Tris-HCl buffer (5mM Tris-HCl /50 mM NaCl, pH = 7.2) solution was guaranteed to be pure and contain sufficient amounts of DNA. Using the molar extinction coefficient (6600 M^{-1} cm^{-1}) at 260 nm, it was also possible to measure the DNA concentration per nucleotide and polynucleotide, denoting that the DNA was sufficiently free of protein by the ratio of absorbance at 260 and 280 nm of roughly 1.8–1.9 [21, 22]. When raising the concentration of DNA in compound solution, up to the point of completely binding, the decrease in absorbance intensity was carefully observed.

$$\frac{[DNA]}{(\varepsilon_a-\varepsilon_f)} = \frac{[DNA]}{(\varepsilon_b-\varepsilon_f)} + \frac{1}{K_b(\varepsilon_b-\varepsilon_f)} \quad \text{(S1)}$$

$$\frac{(\varepsilon_b-\varepsilon_f)}{(\varepsilon_a-\varepsilon_f)} = \frac{1}{K_b[DNA]} + 1 \quad \text{(S2)}$$

$$\frac{1}{(A_X-A_0)} = \frac{1}{(A_{max}-A_0)\,K_b[DNA]} + \frac{1}{(A_{max}-A_0)} \quad \text{(S3)}$$

Where, $\frac{(\varepsilon_b - \varepsilon_f)}{(\varepsilon_a - \varepsilon_f)} = \frac{(A_{max} - A_0)}{(A_X - A_0)}$

$$\frac{(A_{max} - A_0)}{(A_X - A_0)} = \frac{1}{K_b[DNA]} + 1 \qquad (S4)$$

$$\frac{A}{(A_0 - A)} = \frac{1}{K_b[DNA]} + 1 \qquad (S5)$$

$$\log\left(\frac{A}{A_0 - A}\right) = \log\left(\frac{1}{[DNA]}\right) + \log\left(\frac{1}{K_b}\right)$$

$$\log\left(\frac{1}{[DNA]}\right) = \log\left(\frac{A}{A_0 - A}\right) + \log K_b \qquad (S6)$$

ε_a is represented as the apparent absorption coefficient value for the MLCT band at a specific concentration of deoxyribonucleic acid and evaluated from Abs/[complex]. ε_f and ε_b are the absorption coefficients for the chemical substance alone and completely interacted with deoxyribonucleic acid, respectively. $\Delta A_{max} = (A_{max} – A_0)$; $\Delta A = (A_x – A_0)$, where A_0, A_x and A_{max} are denoted as the optical density of chemical substances alone, the middle form, and the completely interacted form with deoxyribonucleic acid, respectively.

$$\Delta G_b^{\circ} = -\,\mathrm{RT}\ln K_b \qquad (S7)$$

$$\%\,H = \frac{(\varepsilon_b - \varepsilon_f)}{\varepsilon_f} \times 100 \qquad (S8)$$

2.2.3. Evaluation of biothermodynamic properties

The thermal denaturation study of CT-DNA is an efficient tool for examining the stability of test compounds bound to double-stranded DNA. The T_m of DNA reveals the change from double to single stranded DNA. The biothermodynamic parameters also involve powerful proof for binding between test compounds and DNA. The denaturation of the biomolecules depends on T_m, pH and non-physiological concentrations of salt, organic solvents and other chemical agents, etc. The DNA double helix breaks into two single strands as the temperature or pH rises, which is ascribed to the breaking of hydrophobic stacking attractions and the dissolution of H-bonds between the DNA base pairs as a result of the hydroxide ions'

withdrawing of the hydrogen ions (H^+) from DNA base pairs. It causes a hyperchromic shift at 260 nm and found that the rate of denaturation is directly proportional to absorbance and inversely proportional to viscosity. Low salt concentration, conversely, can also denature DNA double-strands by eliminating the ions that stabilize the negative charges on the two strands from each other. By using UV-Vis analysis in a temperature-controlled sample holder, DNA thermal denaturation was carried out both in the absence and presence of the test compounds. In 5mM Tris-HCl /50 mM NaCl buffer (pH = 7.4) solution, each test substance (50 µM) was incubated in a 1:1 ratio with CT-DNA (50 µM). All samples were heated in the range of 25 to 100 °C at a rate of 5 °C per minute and the absorbance changes at 260 nm were constantly monitored. T_m of DNA alone was evaluated as the midpoint of the sigmoid' curves plotted absorbance *vs* temperature profiles. The T_m is depends on the double-stranded DNA stability. Due to substantial intercalation in DNA-complex adducts, the reduction in DNA absorbance was seen in the presence of test compounds at room temperature. When increasing temperature, double-stranded DNA converts into two single strands at the melting point, which leads to hyperchromic effect due to reducing the base-base interaction. As compared to DNA alone at the same high temperature, the incubated DNA solution with test compounds leads to hyporchromic effect accompanied with red shift. Strong interactions between complexes and DNA, which restrict the separation of the double helix, cause the stabilization, and as a result, T_m rises. In accordance with the T_m, 50% of DNA is in its native double-stranded state, and the remaining 50% is in its denaturalized single-stranded state [23–25].

$$\ln\left[\frac{K_2}{K_1}\right] = \left[\frac{\Delta H^\circ}{R}\right]\left[\frac{T_2 - T_1}{T_1\,T_2}\right] \quad \text{(S9)}$$

$$\Delta G^\circ = -R.T_m.\ \ln K_m = \Delta H^\circ - T_m\,\Delta S^\circ \quad \text{(S10)}$$

Where K_1 is represented as the binding constant value at 298 K (T_r), the binding constant K_2 (K_m), which indicates the temperature at which substances melt their DNA (T_m K), This

technique offers crucial data on binding constants and associated ΔG°, ΔS° and ΔH° findings for compound-deoxyribonucleic acid systems.

2.2.4. Viscometry-based titration for investigation of DNA binding

The Viscometry-based titration measurements were further supportive and delivered data about the binding modes between test compound and DNA base pairs. Nevertheless, it does not deliver definite confirmation for the exact mode of interactions. The experiment performed for all test compounds and ethidium bromide (control) at various concentrations 20, 40, 60, 80, 100 μM and each compound mixed with the CT-DNA solution (100 μM) in the Ostwald viscometer. With an accuracy of 0.01 seconds, a digital stopwatch was used to measure the average flow time. Due to the enhancement of the DNA's overall contour length during the separation of base pairs at intercalation sites, the classical intercalative mode significantly increases the viscosity of the DNA solution. Generally, the compounds may result in less positive/negative or no viscosity change if they bind with DNA base pairs *via* major or minor grooves [26]. It is found that viscosity also rises while it binds with test compounds *via* intercalation.

$$\left(\frac{\eta}{\eta_0}\right)^{1/3} = \frac{\left\{\frac{(t_{complex} - t_0)}{t_0}\right\}}{\left\{\frac{(t_{DNA} - t_0)}{t_0}\right\}} \qquad (S11)$$

2.2.5. DNA/BSA binding assay by fluorescence titration

The titration was executed by the JASCO FP-6300 spectrofluorometer in the range of 200–800 nm, which is also supported to prove the interacting mode of all test compounds with DNA. To assess the relative DNA binding abilities of the test compound to CT-DNA, ethidium bromide (EB) was utilized as a reference. In the experiment, EB solution (20 μM) in 50 mM Tris–HCl /1 mM NaCl buffer solution (pH=7.2) was incubated with the DNA

solution (240 μM) at saturated binding levels and stored in a dark place for two hours. The tested compounds (1–240 μM) were titrated with pre-incubated EB-bound DNA. Additionally, from 610 nm to 510 nm, in the absence and presence of DNA (240 μM), the intensity changes for initial emission and excitation of EB were cautiously observed. As a conjugate planar molecule, EB's acquired fluorescence intensity is extremely low, but it increases significantly as it intercalates with DNA base pairs. While increasing the concentration of the compounds (0-240 μM), the intensity of the EB-DNA system decreases owing to the quenching effect of DNA by the manner of static quenching. It further attributes the generation of ground state DNA – complex adduct owing the cationic metal complexes interacting with the negatively charged oxygen in phosphodiester of DNA and the contraction of DNA pushes a few EB molecules out of DNA base pairs [27]. Also, Emission titration experiments were measured for BSA interaction in the range of 300–400 nm, using a fluorescence cuvette with a path length of 1 cm. all test compounds have no substantial fluorescence emission rate in any of the above ranges in Tris-HCl buffer solution (pH=7.2). Therefore, BSA interaction tests are not impaired in the interaction with the complexes [28]. In the experiment, the emission of BSA solution (25 μM) in 50 mM Tris–HCl /1 mM NaCl buffer solution (pH 7.2) was observed at 350 nm. While raising the test compound's concentration (0-25 μM), the intensity of the BSA decreases due to static quenching in the generation of ground state BSA – complex adduct. The observations of the BSA molecule at the excitation wavelength (278 nm) and at the emission wavelength (350 nm) that both of them did not contribute to the inner filter effect, as evident from their very low absorbance values.

$$F_{corr} = F_{obs} \times e^{[(A_{ex} \times d_{ex}) + (A_{em} \times d_{em})]/2} = F_{obs} \times e^{(A_{ex} + A_{em})/2} \quad (S12)$$

F_{corr} and F_{obs} are represented as the IFE-corrected emission and observed (uncorrected) emission intensities, respectively. d_{ex} and d_{em} are denoted as the cuvette path lengths in the

excitation and emission directions, respectively. Also, the alteration in absorbance (OD) at the excitation and fluorescence wavelengths, respectively, is sometimes referred to as A_{ex} and A_{em}.

$$\frac{F_0}{F} = 1+K_{SV}\ [Q] = 1+ K_q\ \tau_0 \qquad \text{(S13)}$$

Where [Q] is represented as the sample concentration (quencher), the emission intensities F_0 and F are those in the presence and absence of the quencher, respectively.

$$\frac{F_0}{F} - 1 = \frac{F_0 - F}{F} = K_{SV}\ [Q]$$

$$\log\left(\frac{F_0 - F}{F}\right) = \log K_{ass} + n\log\ [Q] \qquad \text{(S14)}$$

$$K_{EB}[EB] = K_{app}[compound] \qquad \text{(S15)}$$

$$\left(\frac{1}{F_0 - F}\right) = \frac{1}{F_0\ K_{LB}\ [Q]} + \frac{1}{F_0} \qquad \text{(S16)}$$

$\Delta F = (F_0–F)$, Equation (16) was utilized to examine the Lineweaver–Burk binding constant (K_{LB}) from the linear regression plot of $1/(F_0–F)$ *vs* 1/[Q].

$$\left(\frac{\gamma}{C_F}\right) = K_{SC}\ (n - \gamma) \qquad \text{(S17)}$$

$\gamma = (F_0–F)/F_0$, C_F is denoted as the concentration of sample alone, the linear regression plot of (γ/C_F) *vs* γ was employed to support equation (17), which was used to determine the values of K_{SA} and n, respectively.

2.2.6. FRET calculation by Förster's theory

Calculating FRET (Fluorescence Resonance Energy Transfer) is a crucial approach in determining the critical distance (r) between donor (BSA) and acceptor (test compound) molecules, in which the binding affinity between the BSA-complex systems could be evalued according to Förster's theory. The overlap of the emission spectrum of the donor (BSA) and the absorption spectrum of the acceptor (compound) describes that the acceptor (compound) group may get excited as a result of the excited donor's (BSA) energy being lost to the ground

state. Resonance refers to the phenomenon of matching energy. Therefore, the more overlap of spectra, the better a donor can transfer energy to an acceptor. Overlapping spectra are represented by the overlap integral J(λ) between the donor and acceptor. Energy (not an electron) transfers from the excited donor to the acceptor group via the non-radiative process, which is described as "resonance". This phenomenon is the Förster energy transfer, which is highly distance-dependent and therefore allows one to probe biological structures. FRET is also known as the spectroscopic ruler due to its intrinsic expediency. A donor group (D) is excited by a photon and then relaxes to the lowest excited singlet state, S_1 (by Kasha's rule). If the acceptor group is not too far, the energy librated while the electron returns back to the ground state (S_0) may concurrently excite the donor group. After excitation, the excited acceptor discharges a photon and returns back to the ground state, if the other quenching states do not exist. When overlapping the emission spectrum of the donor and the absorption spectrum of the acceptor, the energy is lost from the excited donor to the ground state, which further excites the acceptor group. The energy matching is known as the resonance phenomenon and the overlap of spectra between the donor and the acceptor is described as the overlap integral J (λ). Also, the FRET efficiency is enhanced while the donor group has good capabilities to absorb and emit the photons, which means that the donor group should have a large absorption coefficient and a large quantum yield. Thus, the more overlap of spectra, the better a donor can transfer energy to the acceptor [29–31].

$$\mathrm{E} = \left(1 - {}^{\mathrm{F}}/_{\mathrm{F_0}}\right) = \ [\mathrm{R_0^6} \ / \ (\mathrm{R_0^6} + \mathrm{r^6})] \qquad \text{(S18)}$$

When transmit efficiency is 50%, the observed critical distance is R_0, which is denoted as the Förster radius characterizing the donor/acceptor pair and is evaluated from the equation (S19).

$$\mathrm{R_0^6} = 8.79 \ \times 10^{-25} \ \mathrm{K^2} \, n^{-4} \Phi \mathrm{J} \qquad \text{(S19)}$$

The relative orientation factor of the dipoles (K^2) is associated with the geometry of the BSA and complex of the dipoles, the value for random orientation (K_2 = 2/3) like in a fluid solution.

$$J = \left[\frac{\int_0^{\infty} F(\lambda)\, \varepsilon(\lambda)\, \lambda^4\, d\lambda}{\int_0^{\infty} F(\lambda)\, d\lambda} \right] \qquad \text{(S20)}$$

F(λ) is represented as the corrected or the normalized emission intensity(OD) of the bovine serum albumin in the range of λ–(λ+Δλ), ε(λ) is denoted as the molar extinction coefficient of the compound at λ.

$$\Phi = \frac{\tau}{\tau_0} = \frac{k_r}{k_r + k_{nr}} = \frac{k_r}{k_r + k_{nr} + k_q\,[Q]} \qquad \text{(S21)}$$

The radiative, non-radiative decay and quenching rate constants are denoted as k_r, k_{nr} and k_q, respectively, and the concentration of complex (quenching species) is described as [Q].

$$k_{ET} = \frac{1}{\tau_0}\left(\frac{R_0}{r}\right)^6 = KJ\, e^{\left(-\frac{2r}{L}\right)} \qquad \text{(S22)}$$

$$B = \Phi\, \varepsilon \qquad \text{(S23)}$$

2.2.7. Evaluation of DNA interaction properties using cyclic voltammetry technique

The electrochemical analysis is a critical entity to examine the electron transfer reactions of the test compounds. With a scan rate of 0.1V, the CHI 620C electrochemical analyzer was utilized with a three-electrode system consisting of glassy carbon as the working electrode, Ag/AgCl as the reference electrode, and platinum wire as the counter/auxiliary electrode to monitor changes in peak current and peak potential during the interaction between test compound and DNA. The changes in peak current and peak potential further afford data on the binding constant as well as the mode of interaction for test compound–DNA adducts. In single-compartment cells of 5–15 mL, electrochemical titrations for all test compounds were executed at 25 °C in the absence and presence of DNA. The solutions were then deoxygenated by passing them through N_2 before measurements were made. The electrochemical analysis for free compounds was executed at a 10 μM concentration in the presence of 5mM Tris-HCl /50 mM NaCl (pH = 7.2) buffer solution. Changes in the anodic

and cathodic peak currents, as well as potential shifts, have been observed as CT-DNA concentrations in each solution of complexes rise from 1 to 10 μM [32].

$$\log\left(\frac{I_0 - I}{I}\right) = \log[DNA] + \log K_b \qquad (S24)$$

The peak currents of the chemical in the absence and presence of deoxyribonucleic acid are designated as I_0 and I, respectively.

$$\log[DNA] = \log\left(\frac{I_0 - I}{I}\right) + \log\frac{1}{K_b}$$

$$\log\left(\frac{1}{[DNA]}\right) = \log\left(\frac{I}{I_0 - I}\right) + \log K_b \qquad (S25)$$

K_b values for all samples were estimated through the linear regression plot of log (1/[DNA]) versus log (I/I_0–I) by method-I. Equation (S26) was attained from equation (S25).

$$\left(\frac{I_0 - I}{I}\right) = K_b[DNA] \qquad (S26)$$

$$\left(\frac{I_0 - I}{I}\right) = \left(\frac{C_b}{C_f}\right) \qquad (S27)$$

C_f and C_b are represented as the free substance concentration and DNA-interacted compound, respectively, and equation (S28) was obtained by comparing equations (S26) and (S27).

$$\left(\frac{C_b}{C_f}\right) = K_b[DNA] \qquad (S28)$$

$$\left(\frac{C_b}{C_f}\right) = \frac{K_b[\text{free basr pairs}]}{S} \qquad (S29)$$

$$\left(\frac{C_b}{C_f}\right) = \frac{K_b[DNA]}{2\,S} + 1 \qquad (S30)$$

$$E_s^{\circ} = \left(E_b^{\circ} - E_f^{\circ}\right) = \frac{0.0591}{n}\log\left(\frac{K_{[red]}}{K_{[oxi]}}\right) \qquad (S31)$$

$$\left(\frac{K_{[red]}}{K_{[oxi]}}\right) = \text{Ant.}\log n\left(\frac{\left(E_b^{\circ} - E_f^{\circ}\right)}{0.0591}\right) \qquad (S32)$$

$$I_p^2 = \frac{1}{K_b\,[DNA]}\left(I_{p_0}^2 - I_p^2\right) + I_{p_0}^2 - [DNA] \qquad (S33)$$

$$I_{pa} = 2.69 \times 10^5\, n^{3/2}\, \alpha^{1/2}\, A\, C_0^*\, D_0^{1/2}\, \nu^{1/2} \qquad (S34)$$

I_{pa} is denoted as the anodic peak current in amperes, n is represented as the number of electrons participating in the redox (M^{1+}/M^{2+}) process (n=1), The activation coefficient (charge transfer coefficient) is shown by the symbol α. and the value is equal to 0.5, which is a typically supported approximation for numerous quasi-reversible systems.

$$I_{pa} = 13314.7 D_0^{1/2} \, v^{1/2} \quad \text{(S35)}$$

2.2.8. UV-Vis absorption titrations for the BSA binding assay

The test compound concentrations (0–25 μM) were varied during the electronic absorption titrations, which were conducted at a fixed BSA concentration (25 μM) in aqueous media (pH = 7.2) at 25 °C. The property of BSA is also confirmed by the observation of a strong absorption band in the UV region at 278 nm. The BSA property is further supported by the evaluation of a strong absorption band in the UV region at 278 nm. The increment of the test compound causes a gradual increase in the BSA absorption band, which leads to the hyperchromism effect with hypsochromic shift, indicating that structural changes happened owing to non-covalent interaction of the test compound with BSA [33].

$$\frac{(A_\infty - A_0)}{(A_X - A_0)} = \frac{1}{K_b[\text{Compound}]} + 1 \quad \text{(S36)}$$

$\Delta A_{max} = (A_\infty - A_0)$, $\Delta A = (A_x - A_0)$, A_0 , A_x and A_∞ are denoted as the absorbance of free BSA, the absorbance of BSA in the increment concentrations of the compound, and the absorbance value of the fully bound form of bovine serum albumin with substance, respectively.

2.3. DFT and molecular modelling studies

DFT calculations were performed to comprehend the electronic structure and reactivity of ligand (**HL**) and metal complexes (**1b-2b**) using the Gaussian 09 package [34]. Geometries were fully optimized both in the gas phase and methanol solution using B3LYP, and comparatively later with the CAM-B3LYP and M06-2X functionals. For each complex, the

transition metal was treated by the LAL2DZ basis set, while 6-31G(d) was employed for the rest of atoms. In line with several previous studies, the solvent effect was accounted for in the framework of the Polarizable Continuum Model using the integral equation formalism (IEF-PCM) [35, 36]. Furthermore, molecular docking studies were performed using Autodock Vina to get insight into the binding profile of ligand (**HL**) and complexes (**1b-2b**) into the active sites of the bovine serum albumin (BSA), SARS-CoV-2 main protease (Mpro) and the calf-thymus DNA dodecamer (CT-DNA). The preparation of the host macromolecules involved the automated addition of missing H-atoms and the removal of water molecules as well as the co-crystallized native ligand of Mpro. The active sites were defined as in our previous studies. The best docking poses were visualized using Discovery Studio [4, 37–39].

2.4. UV-Vis absorption titrations for in vitro antioxidant assay

The electronic absorption technique was used to assess the scavenging activities of all test compounds at concentrations of 40, 80, 120, 160, 200, and 240 μM. The IC_{50} observations for all test compounds were compared with standard ascorbic acid. As per the technique coined by Choi et al., the DPPH radical scavenging evaluation of all test compounds was executed in the presence of 2, 2-diphenyl-1-(2,4,6-trinirophenyl)hydrazyl (DPPH) free radical. In order to reduce the loss of free radical activity in the DPPH stock solution, the absorbance of DPPH alone was measured at 517 nm. Hydroxyl radical scavenging activity for all test compounds was measured by the method formulated by Avani Patel et al. 3 mL of pyrogallol solution (0.05 M pyrogallol in 0.05 M phosphate buffer, pH 6.5) and 0.05 to 0.1 mL of different concentrations of all test samples were pipetted out into cuvettes and 0.5 mL of 1 % hydrogen peroxide was introduced in the test cuvettes and monitored the changes in absorbance for every 30 seconds up to 3 minutes at 430 nm. The superoxide radical scavenging activity for all test compounds was evaluated by the method invented by Nishikimi et al. This activity

also was usually observed by the reduction of nitroblue tetrazolium (NBT) solution, which is blended in a test tube and the reaction mixture was diluted up to 3 mL with phosphate buffer. The absorbance of the solution was noted the following illumination for 5 minutes at 590 nm. Higher absorbance denotes lesser antioxidant power. The nitric oxide scavenging property was also analyzed by the Griess Illosvay reaction method. Sodium nitroprusside 5mM was prepared with the help of phosphate buffer pH 7.4. The different concentrations of all test compounds (1 mL) were added with 0.3 mL of sodium nitroprusside. After 2-3 hours of incubation at 25 °C with the resulting test solutions, 0.5 mL of Griess reagent was introduced. When nitrite was diazotized with sulphanilamide and then coupled with naphthyl ethylene diamine, the chromophore's absorbance was measured at 546 nm [40–42].

$$\text{Scavanging (\%)} = \left[\frac{(A_0 - A_S)}{A_0}\right] \times 100 \qquad \text{(S37)}$$

Where A_0 and A_S are represented as the electronic intensity of free DPPH (control) and DPPH mixed substances in ethanol, respectively.

2.5. Agar disc diffusion method for in vitro antimicrobial characteristics

The agar disc diffusion technique was utilized to test the antimicrobial property for all compounds against selected seven different pathogenic bacteria [*(-) Escherichia coli (**A**), (-) Salmonella enteric serovar typhi (**B**), (-) Chromo bacterium violaceum (**C**), (-) Pseudomonas aeruginosa (**D**), (-) Shigella flexneri (**E**), (+) Staphylococcus Aureus (**F**), (+) Bacillus cereus,(**G**)*] and three different fungal species [*Aspergillus niger (**H**), Candida albicans (**I**), Mucor indicus (**J**)*] [43–45]. Using the subsequent components, the agar nutrient was prepared; Beef infusion (300 g), Acid hydrolysate of casein (17.5 g), Starch (1.5 g) and agar (17 g) was made up in one litre of double distilled water. The finally blended solution was heated in a water bath until the agar completely dissolved, and it was then sterilized for 15 minutes at 121 °C and 15 lbs of pressure in an autoclave. After gelling, the pH of the agar

medium was kept at 7.2 at 25 °C. Some test substances may lose or have greater potency when the pH is very low. The reverse effects could happen if the pH is too high. While still molten, 20 mL of autoclaved Mueller-Hinton nutrient agar medium was poured up to a depth of around 4 mm into 100 mm flat-bottomed sterilized glass/plastic Petri plates. The plates were then uniformly wiped down across a culture plate, permitting them to solidify on a flat table. A refrigerator (2 to 8°C) was used to keep the agar media for 24 hours. Twenty milliliters of Mueller-Hinton medium was placed in Petri plates, which were then seeded or streaked with bacterial and fungal strains for 24 hours. The dehydrated surface of the agar medium inoculated with certain microorganisms was placed with filter paper discs measuring 6mm in diameter that had been coated with specific 20 µL of test compounds (100 µM). The test samples diffuse into the agar medium from the filter paper. The preserved packages of plates were placed in an incubator set at 37 °C for bacterial strains and 22 °C for fungal strains, with each disc pressed down to make sure it made thorough contact with the agar surface. After 48 hours for bacteria and 7 days for fungal strains of incubation, the diameter of clear zone inhibition was evaluated using a ruler under the plate's base. The finally evaluated clearance zone (mm) values were also compared with standard antibacterial drugs *Amikacin, Streptomycin* and antifungal drugs *Ketoconazole, Amphotericin B.*

$$\text{Inhibition}\ \% = \left[\frac{(\mathrm{T-C})}{\mathrm{T}}\right] \times 100 \qquad \text{(S38)}$$

Where T and C are represented as the diameter of microbial growth of the sample plates and the control plate (6 mm), respectively.

2.6. MTT Cell Viability Assay for cytotoxic properties

All substances were examined for their in vitro anticancer characteristics towards the A549, HepG2, MCF-7, and NHDF cell lines by the MTT [3-(4,5-dimethylthiazol-2-yl)-2,5-diphenyltetrazolium bromide] technique. The MTT test is an enzyme-based colorimetric

method that is quantitative, sensitive, and consistently used to measure the viability, cytotoxicity, and proliferation of cells. Following a 24-hour incubation period at 37 °C in a humidified environment with 5 percentage CO_2, axenic cancer cells were plated in a 96-well plate at a density of 1×10^5 cells per well. The cells were then incubated in medium containing varied doses of the test substances (10–100 µg/mL in DMSO). After 24 hrs, the cell medium was separated out and washed with sterile phosphate buffer saline (PBS), 190 ml RPMI-1640 sodium bicarbonate buffer system, which is utilized to keep physiological pH and 10 mL of the tetrazolium dye (5mg ml^{-1}) solution was introduced to each well and the cells were incubated for an additional four hrs at 37 °C. The viable cells consists of NAD(P)H-dependent oxidoreductase enzymes, which diminish the water–soluble yellow MTT tetrazolium salt into water-insoluble purple-blue coloured stable formazan crystals. While the cells die, they lose the colour-changing capability, which is also very supportive and performs as a suitable marker of the viable cells. Then, the medium was separated out and blended with 100 µL of DMSO. After shaking slowly twice for 5 s, the absorbance of the purple-blue formazan dye generated by the cells is evaluated in an ELISA plate reader at 570 nm. The acquired data (mean O.D. ± S.D. from three duplicate wells) were supported to calculate the IC_{50} and compare it to the standard anticancer agent (cisplatin) in terms of the percentage of cell viability/growth inhibition for all test compounds [46].

$$\%\ \text{Growth inhibition}(\text{Cytotoxiciy}) = \left[1 - \left(\frac{\text{Mean absorbance of Sample}}{\text{Mean absorbance of Control}}\right)\right] \times 100 \qquad \text{(S39)}$$

$$\text{Cell viability}(\%) = [100 - \text{Cytotoxiciy}(\%)] \qquad \text{(S40)}$$

(**Note:** Refer to the manuscript for references of the experimental section: 2a (2.1 – 2.6)

3a. Results and Discussions

3.1. Structural Characterization

Table S1 Analytical and physical data of ligand (**HL**) and its mixed ligand complexes (**1b–2b**).

**Compounds (EF & FW)**	**Colour**	**Yield (%)**	**M.P (°C)**	**Found (Calcd) (%)**				**^m**
				**C**	**H**	**N**	**M**	
(**HL**) ($C_{17}H_{18}N_2O_2$) (282.33) Ligand	Dark yellow	87.52	148	72.55 (72.25)	06.53 (06.37)	10.04 (09.91)	----	16.12
(**1b**) ($C_{46}H_{42}N_6O_4$)Cu (805.71)[Cu(L)$_2$(phen)]	Bluish Green	84.51	152	68.64 (68.51)	05.32 (05.21)	10.55 (10.43)	08.01 (07.89)	54.43
(**2b**) ($C_{46}H_{42}N_6O_4$)Zn (807.58)[Zn(L)$_2$(phen)]	Reddish Brown	81.74	168	68.40 (68.35)	05.31 (05.20)	10.46 (10.40)	08.21 (08.10)	37.45

EF → Empirical Formula, FW→ Formula Weight, M.P→ Melting Point, $\wedge_m$ → Molar Conductance

3.1.1. Synthesis of Schiff base ligand (HL)

2-(4-morpholinobenzylideneamino)phenol Schiff base ligand (**HL**) was synthesized by stirring an equal molar quantity (0.01 M) of ortho amino phenol (0.03301g) and 4-(4-morpholinyl) benzaldehyde (0.01 M) (0.0574g) in ethanol (30 ml) and the dark yellow precipitate was obtained after refluxing for three hours and in a water bath, the solution's volume was decreased to one-third and cooled at 25 °C. In vacuum desiccators over anhydrous $CaCl_2$, the gathered pure dark yellow solid ligand was progressively dried at ambient temperature. It was discovered that the isolated ligand's (**HL**) yield was 87.52 percentages (**Scheme 1**).

3.1.2. Synthesis of mixed ligand complexes (1b–2b)

A solution of ligand (**HL**) (primary ligand) (0.002 M) (0.01696 g) in methanol (30 mL) was gradually added to a solution of Metal(II) acetate salts (0.001 M) [$Cu^{II}(OAc)_2.H_2O$ (0.005995g), and $Zn^{II}(OAc)_2.2H_2O$ (0.006592g)] in 30 mL of distilled and after stirring for 30 minutes, the hot solution mixture was gradually added to the methanolic solution of 1, 10 phenanthroline (phen) as secondary ligands (0.001 M) (0.005412 g). The obtained solution

mixture was further refluxed for three hours. The solid product was obtained by filtration and they are also purified by recrystallization technique in the presence of methanol-petroleum ether mixture. Moisture and other solvents were removed by anhydrous $CaCl_2$ in the vacuum desiccators. Similar techniques were used to synthesize mixed ligand complexes (**1b-2b**), and the yield was obtained to be 81.74–84.51 % percentages (**Scheme 1**) and they were summarized in **Table S1**.

3.1.3. Analytical Methods: Elemental Analysis and Molar Conductance Studies

The synthesized ligand (**HL**) and its mixed ligand complexes (**1b**–**2b**) were found to be intensely coloured and they are soluble in CH_3OH, C_2H_5OH, $CHCl_3$ and DMSO. The molar conductance ($\wedge_m$) was observed in the range of 37.45 – 54.43 $Ohm^{-1}\ cm^2\ mol^{-1}$, which was higher than that of free ligand (16.12 $Ohm^{-1}cm^2\ mol^{-1}$). The molar conductance results are attributed that they are non-electrolytes. The analytical information data and physical properties of all test compounds are enclosed in **Table S1**.

3.1.4. Mass Spectra (ESI/LC–MS)

Ligand (**HL**) shows the molecular ion peak at *m/z* 282.3 (M) consequent to $[C_{17}H_{18}N_2O_2]^+$ and other fragmented molecular ion peaks are found at 244.3 (M+1) $[C_{14}H_{15}N_2O_2]^+$, 216.2 (M+2) $[C_{13}H_{14}N_2O]^+$, 189.3 (M+1) $[C_{11}H_{12}N_2O]^+$, 176.3 (M+2) $[C_{11}H_{12}NO]^+$, 162.2 (M+1) $[C_{10}H_{11}NO]^+$ and 118.1 (M+1) $[C_8H_7N]^+$ (**Fig. S1**). Complex (**1b**) molecular ion peak at *m/z* 805.6 (M) corresponding to $[C_{46}H_{42}N_6O_4Cu]^+$ (**Fig. S2**) and other fragmented molecular ion peaks are found at 699.2 (M+1) $[C_{40}H_{37}N_5O_3Cu]^+$, 477.1 (M+1) $[C_{26}H_{27}N_3O_2Cu]^+$, 348.2 (M+2) $[C_{17}H_{18}N_2O_2Cu]^+$ and 283.0 (M+1) $[C_{17}H_{18}N_2O_2]^+$. Complex (**2b**) molecular ion peak at *m/z* 808.2 (M+1) corresponding to $[C_{46}H_{42}N_6O_4Zn]^+$ (**Fig. S3**) and other fragmented molecular ion peaks are found at 700.1 (M+2) $[C_{40}H_{37}N_5O_3Zn]^+$, 526.2 (M-1)

$[C_{29}H_{26}N_4O_2Zn]^+$, 483.0 (M) $[C_{27}H_{22}N_4OZn]^+$, 348.3 (M+1) $[C_{17}H_{18}N_2O_2Zn]^+$ and 283.0 (M+1) $[C_{17}H_{18}N_2O_2]^+$. Also, the generation of other peaks are followed by fragmented molecular ion of ligand (**HL**). The observed similar fragmented molecular ion peaks for other complexes were good agreed with molecular structures [1].

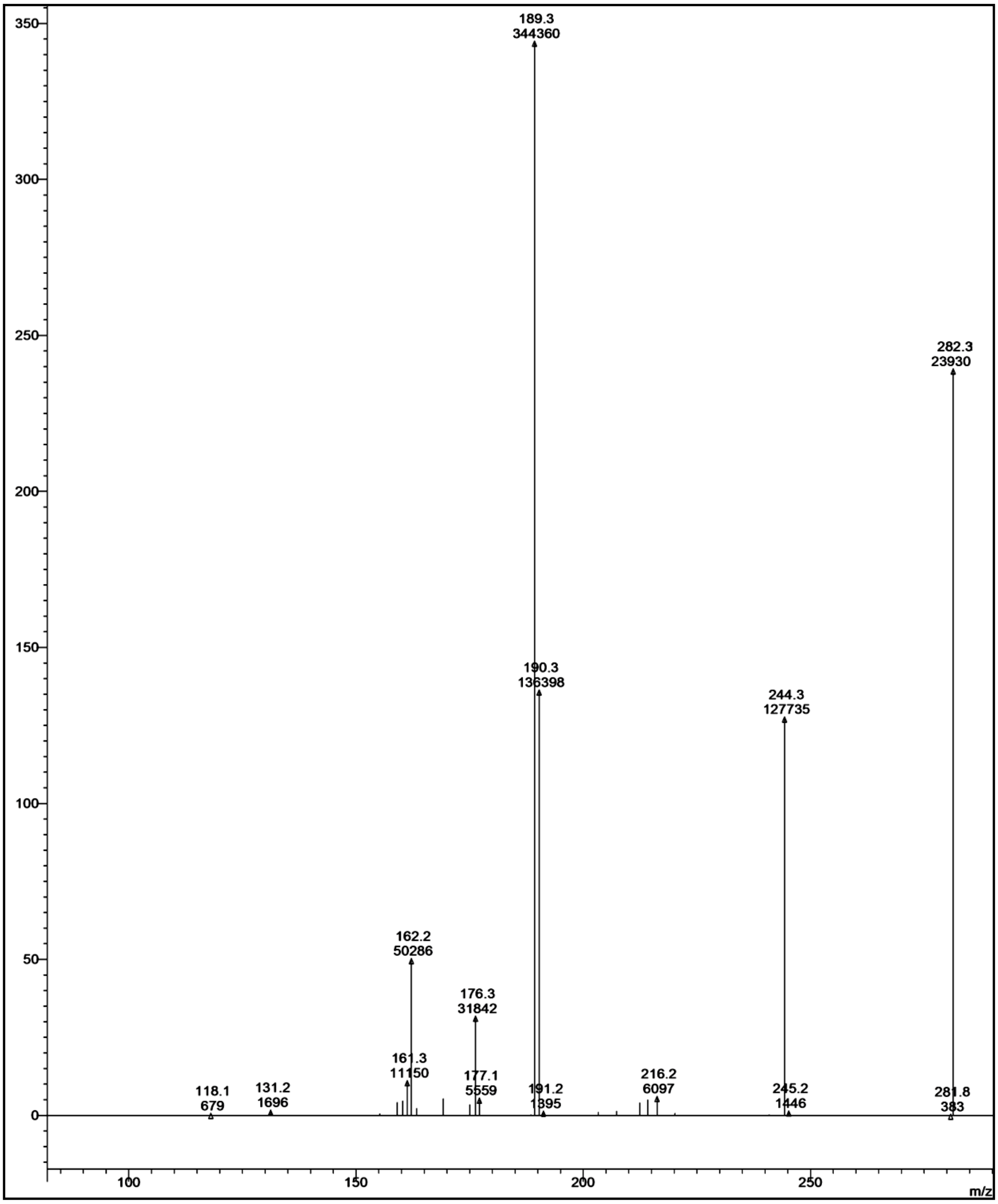

Fig. S1 ESI LC-MS mass spectrum of ligand (**HL**).

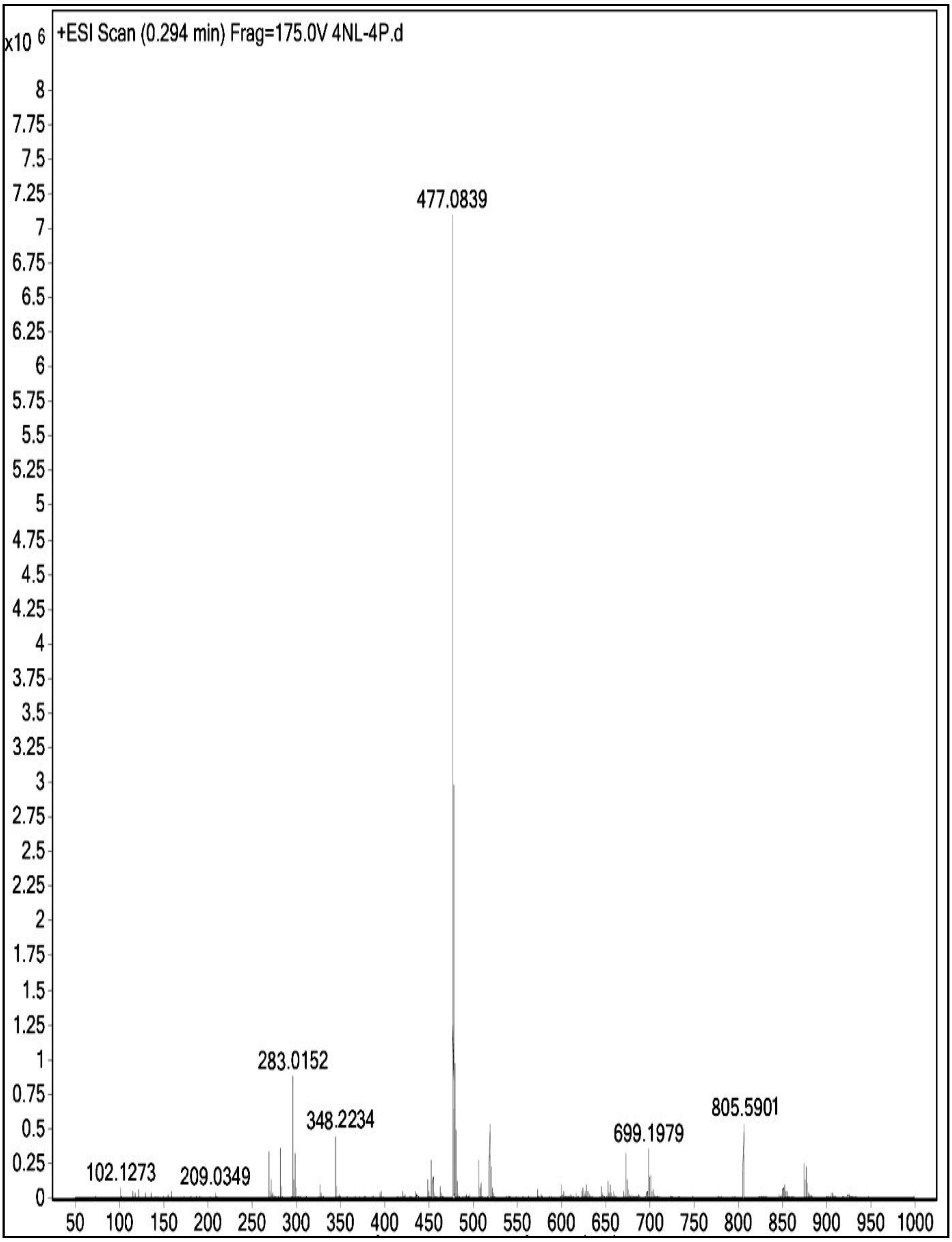

Fig. S2 ESI LC-MS mass spectrum of complex (**1b**)

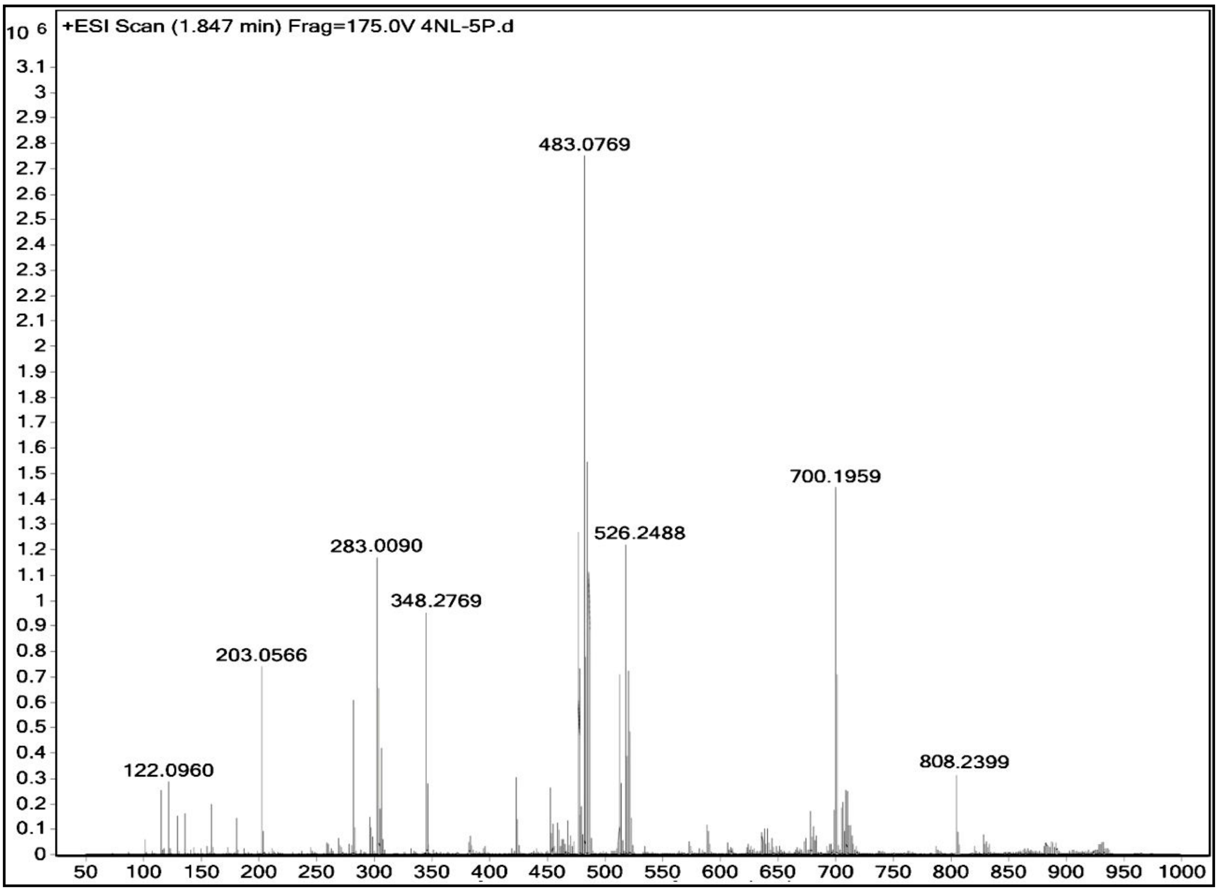

Fig. S3 ESI mass spectrum of complex (**2b**)

3.1.5. Proton Nuclear Magnetic Resonance Spectra (^1H NMR)

The ^{1}H NMR spectral properties were executed in presence of $CDCl_3$ solvent using $Si(CH_3)_4$ as an internal standard. The ligand (**HL**) and its complex (**2b**) demonstrate the following signals in **Figs. S4 & S5** and the observed results were also listed in **Table S2**. δ values of ligand (**HL**): aromatic protons (m, 8H) at 6.45 – 7.65 ppm; azomethine (-HC=N-) proton (s,1H) at 8.58 ppm; morpholinic-OCH_2 protons (t, 4H) at 3.85 ppm; morpholinic-N-CH_2 (t, 4H) at 3.30 ppm; phenolic-OH proton (s, 1H) at 5.10 ppm [2]. The absence of a singlet peak in the range of 13–14 or 5–6 ppm in the complex (**2b**) indicates the loss of the –OH proton due to complexation [3]. Free 1,10' phenanthroline exhibited four sets of resonances, centered at chemical shift (δ) 8.0 ppm (H4, (7.70–8.10) m), 7.26 ppm (H5, (7.15–7.50) dd), 8.81 ppm (H6,(8.70–9.10) dd) and 7. 60 ppm, (H7, (7.55–7.65) m) [4]. These signals (δ) were also

found in the regions at 7.83 ppm (H4, m), 7.15 ppm (H5, dd), 9.80 ppm (H6, dd) and 7.76 ppm, (H7, dd) in complex (**2b**) (**Fig. S5**) [5]. Morpholine fused aromatic protons (m, 8H) were found in the range of 6.88 - 7.84 ppm. It is clearly observed that the azomethine (-HC=N-) (s, 1H) and phen-C=N-CH- protons (6H, dd & 6'H dd) were shifted to downfield at 9.80 ppm and 9.34 ppm respectively due to complexation, which further indicates that the deshielding effect of the electron atmosphere in the phenanthroline ring decreases after complexation of the nitrogen atoms in phen with the Zn^{2+} ion [6–9]. Azomethine and 1,10' phenanthroline iminic proton signals were significantly raised into deshielding due to there have involved in complexation. Furthermore, the strong singlet peak for $CDCl_3$ solvent was observed at 7.26 ppm in both ligand (**HL**) and complex (**2b**) and other signals are no appreciable changes in the complex (**2b**).

Table S2 ^{1}H NMR spectral data of the ligand (**HL**) and its mixed ligand complex (**2b**).

**Compounds (ppm)**	**Ar-protons (m, 8H) / (m, 8H)**	**HC=N- (s,1H)**	**Morp. O-CH$_2$- (t, 4H)**	**Morp. N-CH$_2$- (t, 4H)**	**Phenolic -OH (s,1H)**	**Phen-C=N-CH- (d, H6)**
(**HL**)	6.45 - 7.65	8.58	3.85	3.30	5.10	-
**(2b)**	6.88 - 7.84	9.80	3.88	3.32	-	9.34

HC=N- → Iminic proton, Morp. O-CH$_2$-, N-CH$_2$- → Morpholinic protons, phen-C=N-CH- → 1,10-phenanthroline protons..

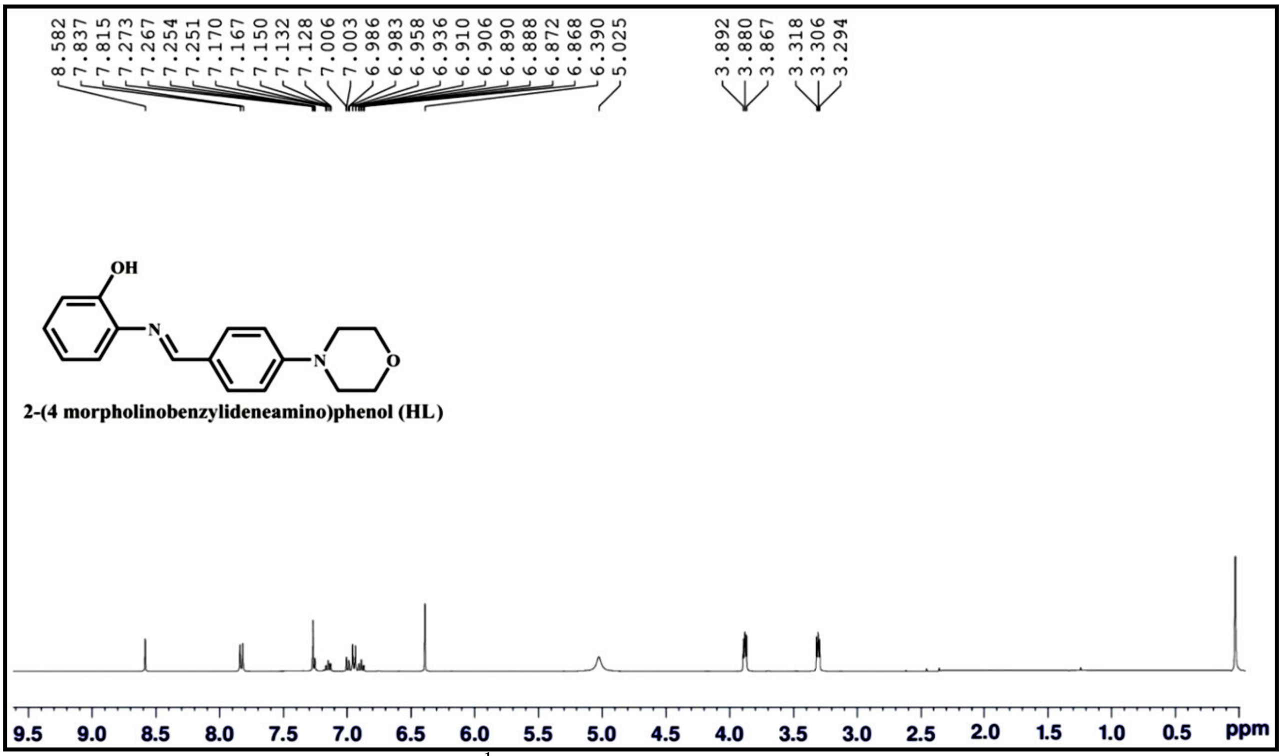

Fig. S4 ^{1}H NMR spectra of ligand (**HL**).

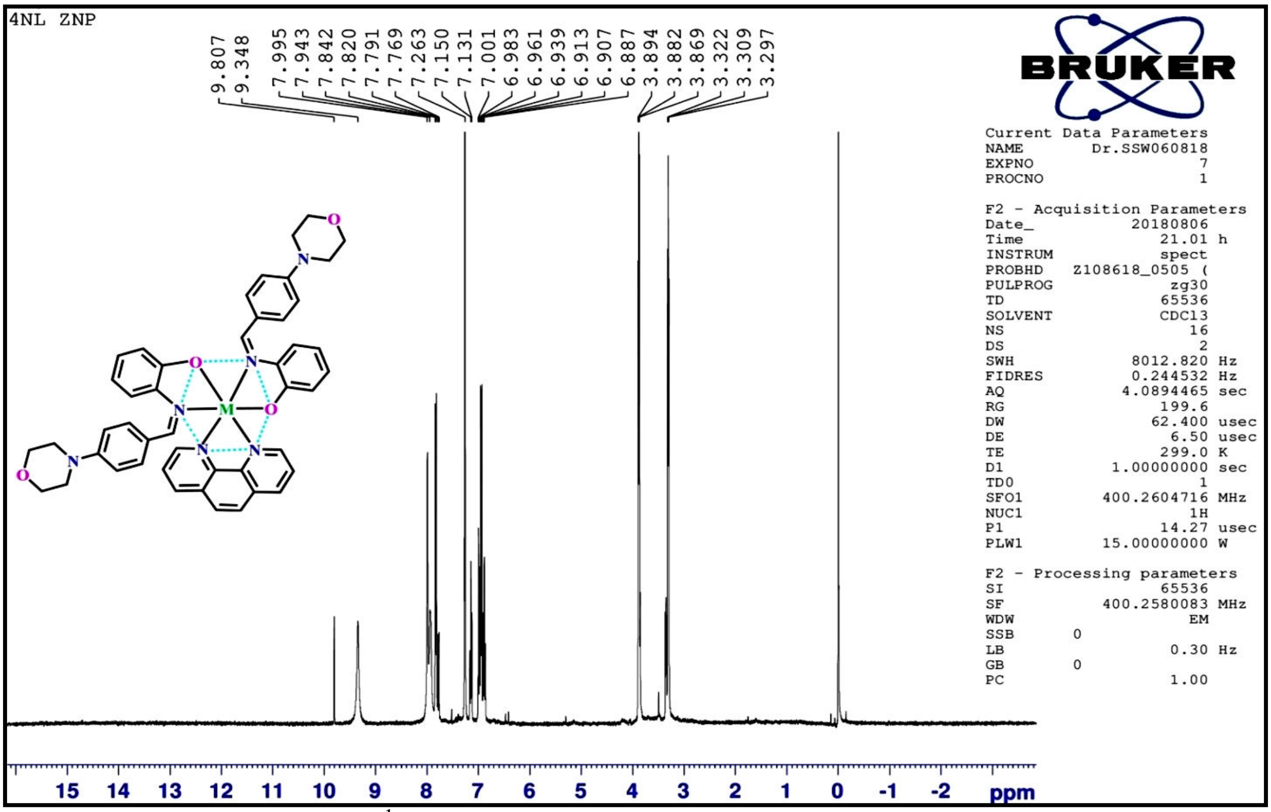

Fig. S5 ^{1}H NMR spectra of mixed ligand complex (**2b**)

3.1.6. ^{13}C NMR spectrum

The ^{13}C NMR spectrum of the ligand (**HL**) and complex (**2b**) were recorded by a Bruker Advance III HD Nanobay 400 MHz spectrometer operating in Fourier transform mode in the presence of $CDCl_3$ at room temperature [10]. The following signals of ligand (**HL**) and complex (**2b**) were demonstrated in **Figs. S6** & **S7**. The observed peak at 156.84 ppm was assigned iminic (azomethine) carbons, which also shifted to the downfield region at164.41 ppm. It indicates the coordination of azomethine nitrogen to the metal centre in the complex (**2b**). The aromatic carbons of the ligand (**HL**) are observed in the range 113.47 – 135.88 ppm. The observed signal in the region 151.95–153.61 ppm indicates the carbon atom adjacent to the phenolic oxygen in the free ligand (**HL**). The signal is also shifted to the downfield region 157.72–161.35 in the complex (**2b**), which reveals that phenolic oxygen coordinated with the metal centre [11]. The peak of morpholinic-$\underline{C}H_2$-O-$\underline{C}H_2$ was found at 66.64 ppm. The chemical shifts for =N-CH_2-$\underline{C}H_2$-N and =N-$\underline{C}H_2$-CH_2-N groups were also obtained at 65.19 and 63.35 respectively. The observed peaks at 47.87 and 47.29 ppm are attributed to morpholinic-$\underline{C}H_2$-N-$\underline{C}H_2$ carbons. Moreover, the observed signals of free 1,10-phenanthroline carbons were in the following region: 151 ppm ($ArC^{1,10}$, 150–152 ppm), 122.5 ppm ($ArC^{2,9}$, 121.5–128.3 ppm), 136 ppm ($ArC^{3,8}$, 135–140 ppm), 129 ppm ($ArC^{4,7}$, 126.6–129.1 ppm), 126 ppm ($ArC^{5,6}$, 123–127.6 ppm) and 145 ppm ($ArC^{11,12}$,144.6–146 ppm) [12–14]. It is also confirmed that the signals for $ArC^{1,10}$, $ArC^{3,8}$ and $ArC^{11,12}$ are shifted to the downfield region at 156.6, 142.55, 151.25 ppm respectively, which indicates that the nitrogen atoms in phenanthroline are coordinated with the Zn^{2+} center (**Fig. S7**)[15]. Furthermore, the strong triplet peaks for $CDCl_3$ solvent were observed in the range of 76.74–77.38 ppm. The observed results also put forward that the locations of carbons in the ligand structure and other signals are no appreciable changes in the complex (**2b**).

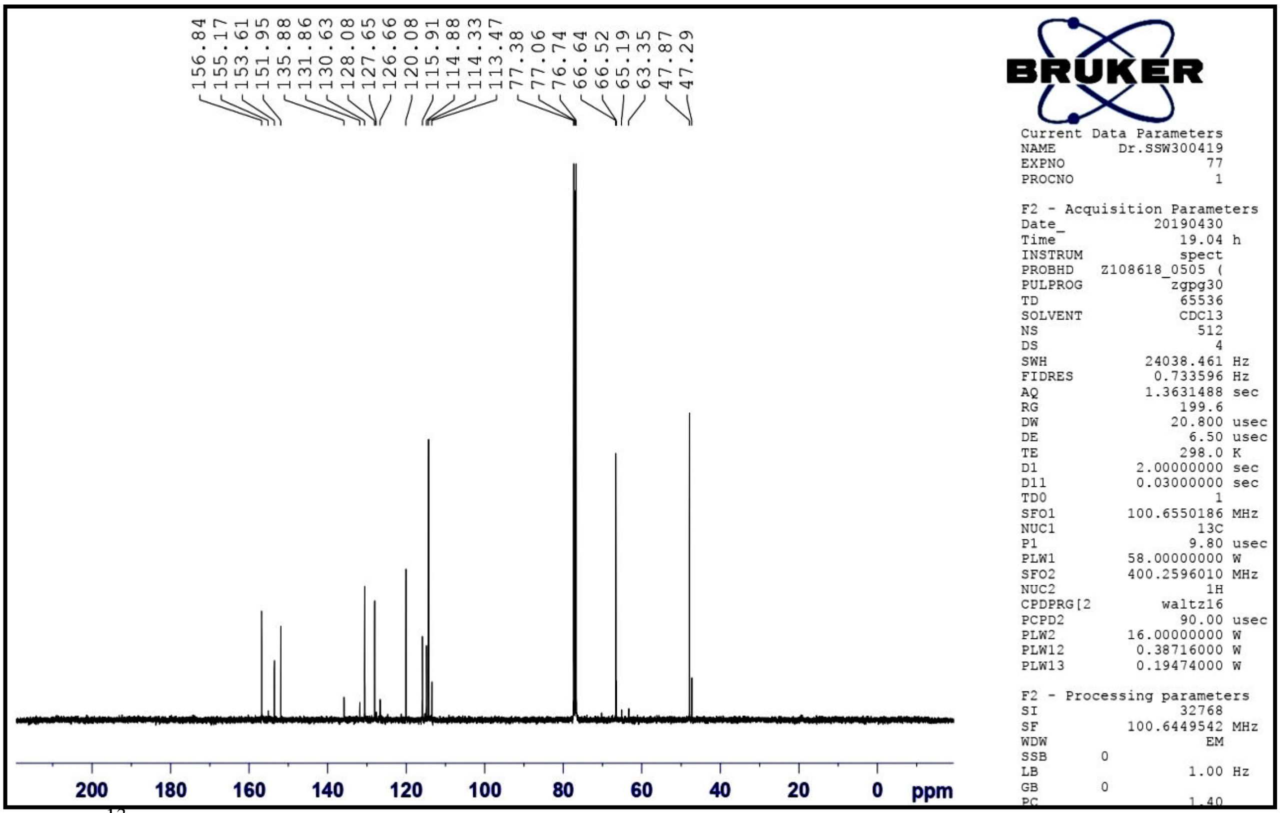

Fig. S6 ^{13}C NMR spectrum of ligand (**HL**).

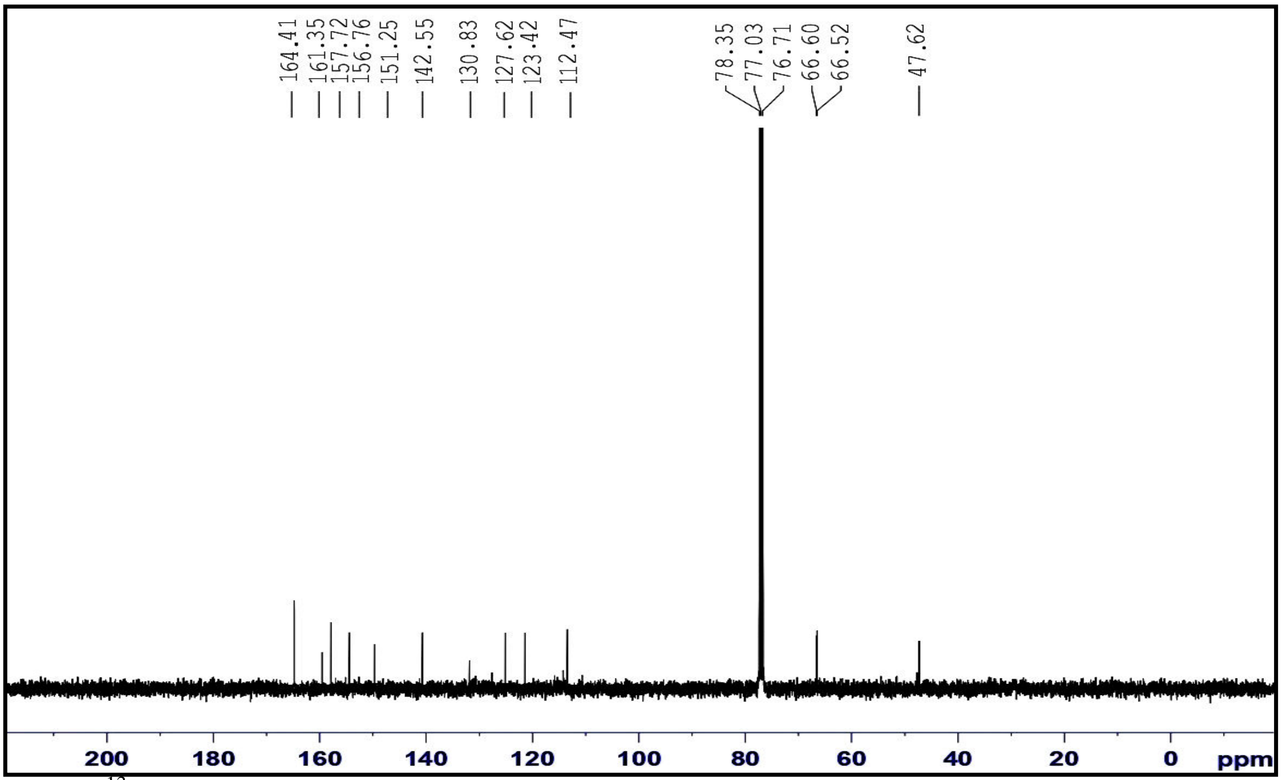

Fig. S7 ^{13}C NMR spectrum of mixed ligand complex (**2b**).

3.1.7. Fourier Transform Infrared Spectra (FT–IR)

The IR spectra were executed using Shimadzu FT–IR spectrometer (4000–400 cm^{-1}) using KBr pellets. The FTIR spectra of the mixed ligand complexes (**1b–2b**) investigated the frequency changes of free ligand (**HL**) due to the complexation and their observed results were also summarized in **Table S3.** FT–IR spectrum of ligand (**HL**) exhibits a strong sharp band of the azomethine group (-HC=N-) at 1632 cm^{-1} the band is also shifted to lower frequencies in the range of 1592 – 1612 cm^{-1} for all complexes due to strong complexation with the central metal ion [16]. The sharp peak of the hydroxy group was identified at 3403 cm^{-1} in the free ligand (**HL**) and the same peak disappeared in the spectra of all complexes due to deprotonation of -OH group during complexation [17]. It is also evidenced that the observed phenolic C-O group at 1268 cm^{-1} in the ligand (**HL**) is shifted to higher frequencies in the range of 1303–1310 cm^{-1} for all complexes (**1b**–**2b**) pointing out confirming deprotonation of the phenolic–OH on chelation [18]. In the FT–IR spectra of all mixed ligand complexes (**1b–2b**), the peaks consequent to the ring stretching frequencies γ(C=N) and γ(C=C) at 1503, 1422 cm^{-1} of free 1,10'-phenanthroline were shifted to higher frequencies in the region 1528–1533 cm^{-1} and 1425–1434 cm^{-1} respectively, which indicate the complexation owing to coordination of the heterocyclic nitrogen atoms to the metal ion [19]. In the far IR spectra of all complexes, the medium bands were found in the region 456 – 464 cm^{-1} and 520 – 528 cm^{-1}, which are consequent to M-N and M-O vibrations respectively and other absorption bands like morpholinic-C-N-C, morpholinic-C-O-C, aromatic C-H and aliphatic C-H have no appreciable changes in the complexes (**1b-2b**) (**Figs. S8, S8a & S8b**).

Table S3 FT–IR spectral data (cm^{-1}) of the ligand (**HL**) and its complexes (**1b**–**2b**).

Compounds	HC=N	Ph-C-O	Morp-C-N-C	Morp-C-O-C	C-H			Ph-OH	Phen C=N & C=C	M-N	M-O
					Ar-C-H	Ali-C-H	Iminic H-C=N				
(**HL**)	1632	1268	1341	1112 (s)<br>1181(as)	2974	2918	2858	3403	---	---	---
(**1b**)	1592	1310	1352	1110 (s)<br>1173(as)	2964	2926	2848	---	1533<br>1425	456	528
(**2b**)	1612	1303	1350	1115 (s)<br>1182(as)	2971	2927	2851	---	1528<br>1434	464	520

s → symmetry, as → asymmetry, Ph-C-O → Phenolic C-O, Morp-C-N-C → Morpholinic C-N-C, Morp-C-O-C → Morpholinic C-O-C, Ar-C-H → Aromatic C-H, Ali-C-H → Aliphatic C-H Ph-OH → Phenolic OH, Phen→ 1,10'-phenanthroline.

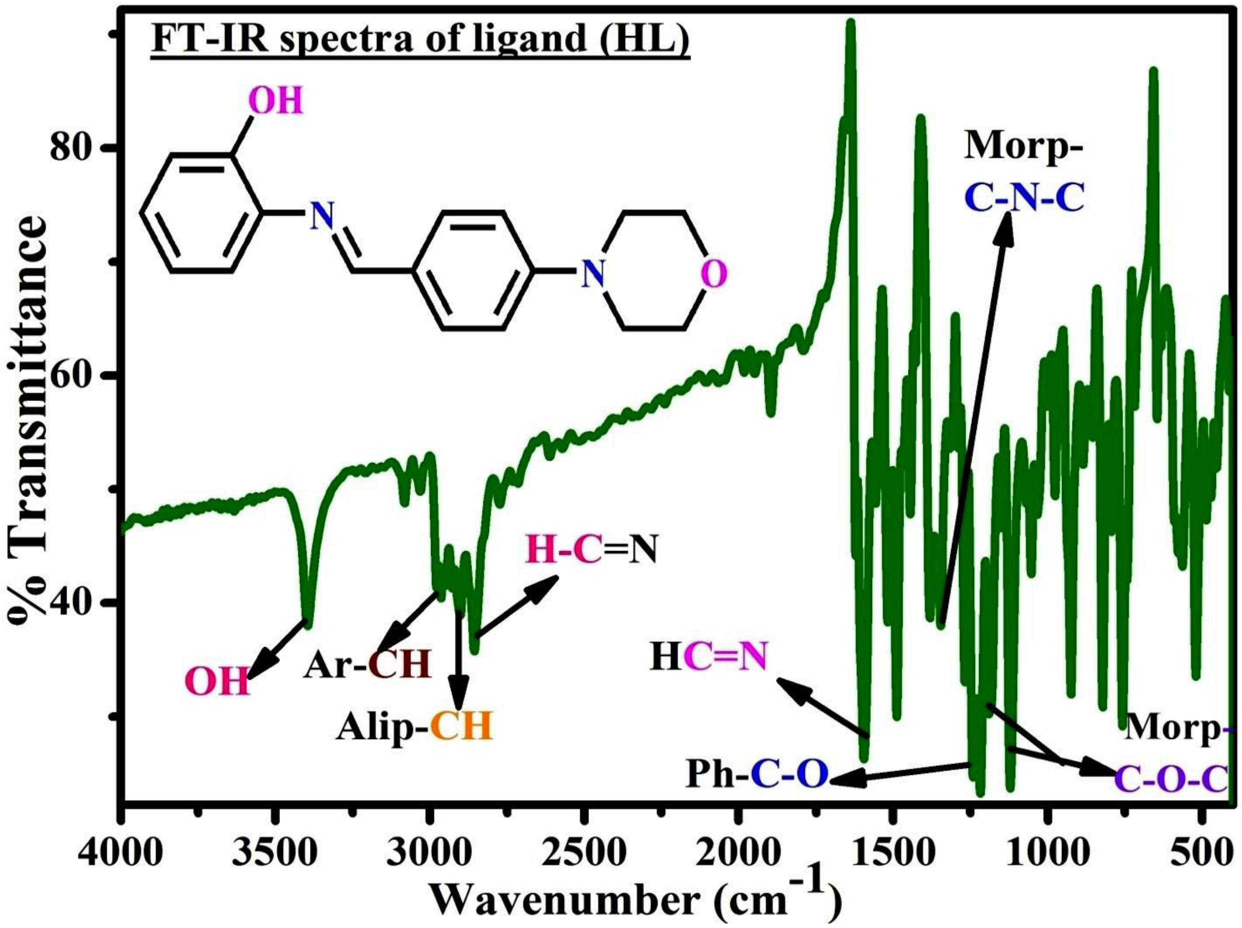

Fig. S8 FT-IR spectrum of free ligand (**HL**)

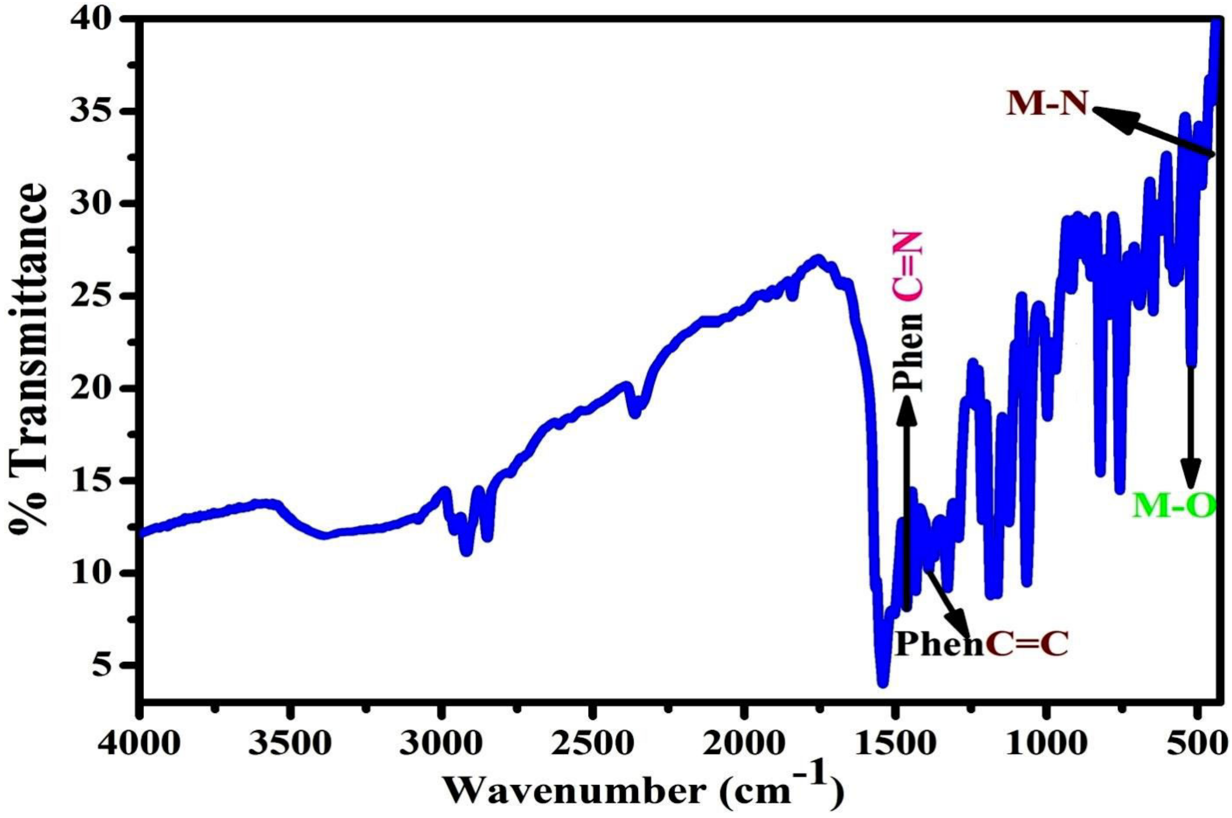

Fig. S8a FT-IR spectrum of complexes (**1b**).

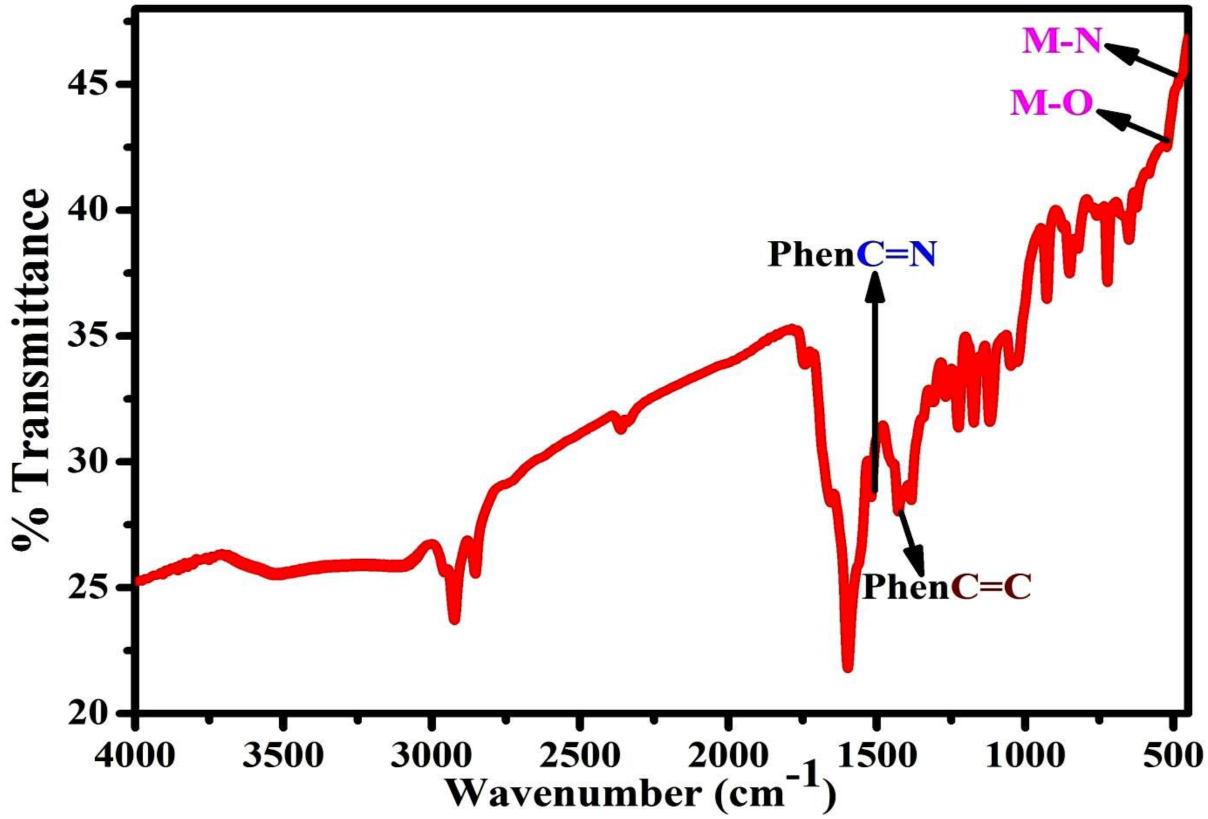

Fig. S8b FT-IR spectrum of complexes (**2b**).

3.1.8. Electronic Absorption Spectra and Magnetic Susceptibility

Ligand (**HL**) and its mixed ligand complex (**1b**) were carried out in methanol by an electronic absorption spectrophotometer. The observed results of absorption maxima and magnetic moment are summarized in **Table S4**. The free ligand (**HL**) exhibited three absorption bands at 358 nm (27,933 cm^{-1}), 247 nm (40,485 cm^{-1}) and 215 nm (46,512 cm^{-1}), which assigns n→π*, π→π* and π→π* transitions respectively owing to the azomethine chromophore and phenyl rings [20]. The bands are transferred into a higher wavelength, which attributes the formation of complex due to the lone pair electron of a sp^2–hybridized orbital of the imino nitrogen in the ligand donates to the metal centre. Moreover, mixed ligand complex (**1b**) revealed only one low-intensity broad band d–d transition at 641 nm (15,600 cm^{-1}) corresponding to $^2Eg \rightarrow ^2T_2g$ owing to dynamic Jahn–Teller distortion and another one band observed at 378 nm (26,455 cm^{-1}) corresponding to intra ligand charge transfer (INCT) with their evaluated effective magnetic moment (μ_{eff}) value at 1.96 B.M, which is faintly higher than the spin-only value 1.73 BM [21]. It suggests the possibility of distorted octahedral geometry (**Table S4 & Fig. S9**). Zinc complex (**2b**) has no appearance of absorption bands in the visible region and CFT does not envisage the d–d transitions due to d^{10} electronic configurations and they are very weakly coloured due to spin forbidden d-d transition and Laporte forbidden. In addition, the obtained μ_{eff} value of the complex (**2b**) was zero, which indicates the diamagnetic nature (**Table S4**) [22]. The obtained results suggest that the complexes (**2b**) belong to an octahedral environment around the central metal(II) ion.

Table S4 Electronic spectral data and magnetic susceptibility values of the synthesized ligand (**HL**) and its mixed ligand complexes (**1b–2b**).

**Compounds**	**Band Position** λ_{max} **nm (γ-cm^{-1})**	**Assignment**	**μ_{eff} (B.M)**	**Geometry**
(**HL**)	358 (27,933)	$n \rightarrow \pi^*$	--	--
	247 (40,485)	$\pi \rightarrow \pi^*$		
	215 (46,512)	$\pi \rightarrow \pi^*$		
(**1b**)	641 (15,600)	$^2E_g \rightarrow ^2T_{2g}$	1.96	Distorted octahedral
	378 (26,455)	INCT		
(**2b**)	437 (22,883)	MLCT	--	--
	336 (29,762)	$n \rightarrow \pi^*$		
	272 (36,765)	$\pi \rightarrow \pi^*$		
	223 (44,843)	$\pi \rightarrow \pi^*$		

INCT→ Intra-ligand charge transfer, μ_{eff} → Effective magnetic moment, B.M→ Bohr magnetons. Wavenumber (γ cm^{-1}) $= \left(\frac{1}{\text{wavelength(nm)}}\right) = \lambda^{-1} \times 10^7$ cm^{-1}. μ_{eff} =0.00 B.M → diamagnetic, MLCT→ metal to ligand charge transfer band

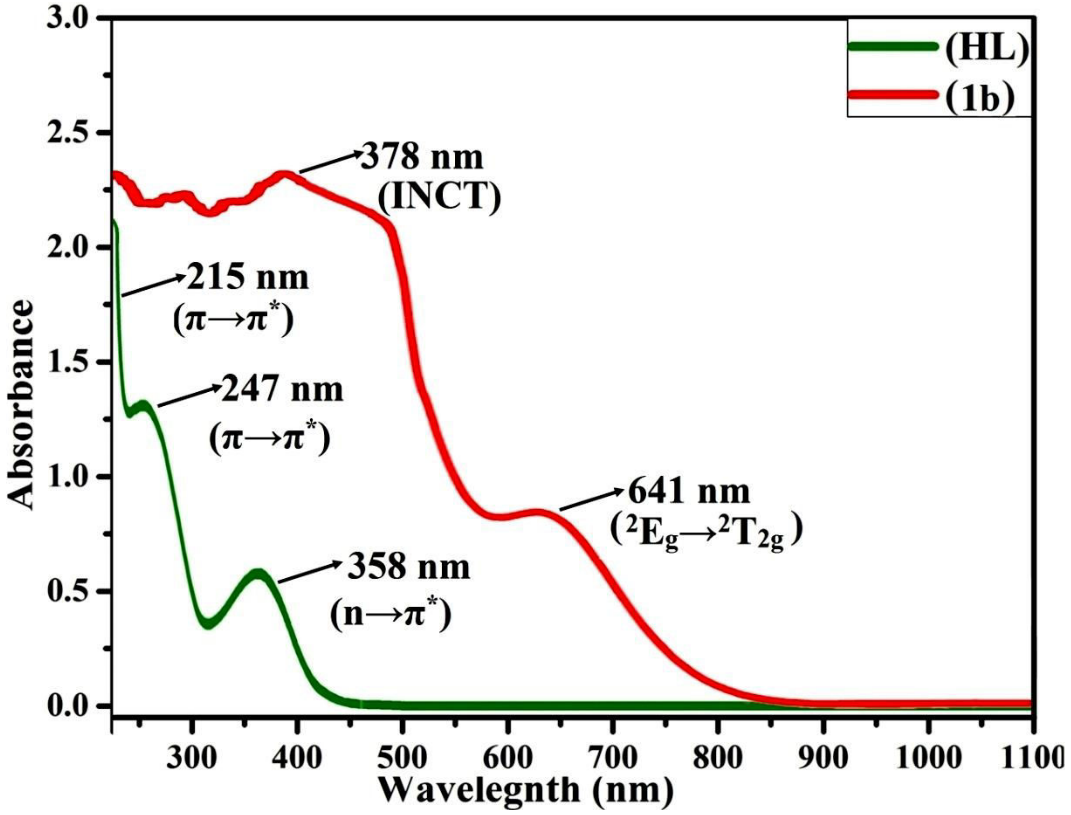

Fig. S9 Electronic spectra of ligand (**HL**) and its complex (**1b**).

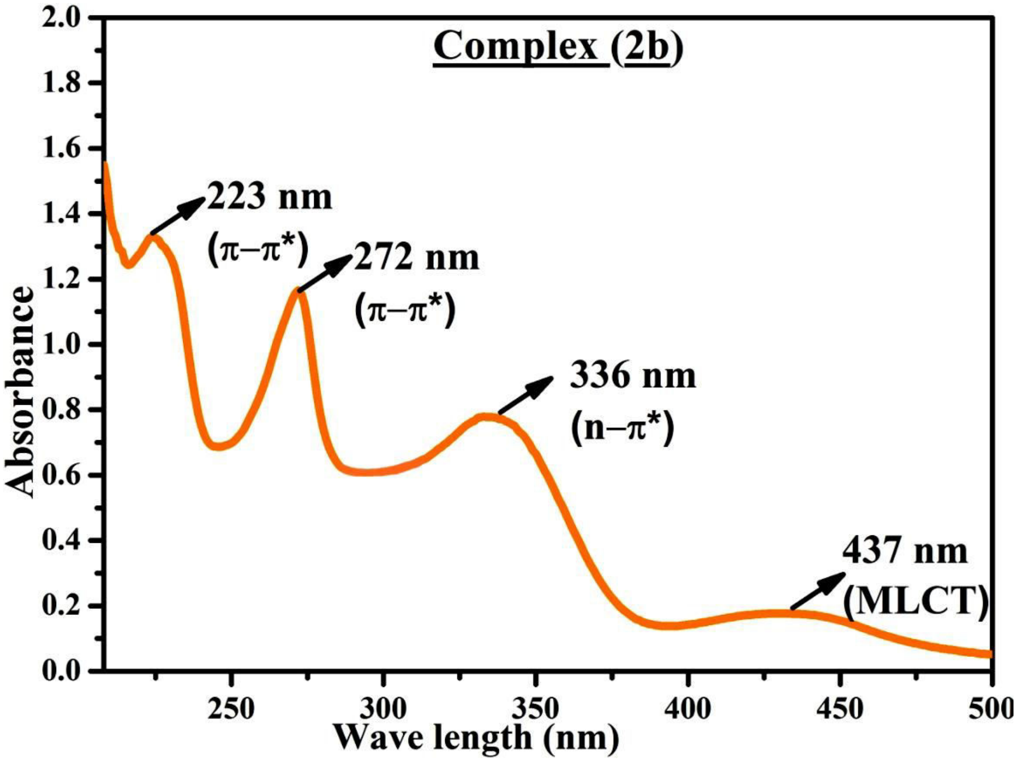

Fig. S9a Electronic spectrum of complex (2**b**).

3.1.9. Electron Spin Resonance Spectra (ESR)

In EPR spectra, copper complex (**1b**) exhibited an anisotropic pattern with well-resolved hyperfine lines in the X-band region at 77 K, which is revealed in **Fig S10** and the obtained results were enclosed in **Table S5**. The measured g-values were found in the following order: $g_{||} > g_{\perp} > g_e$ (2.00277) in the distorted square planar and octahedral copper complexes, which attribute that the unpaired electron lies in the $d_{x^2-y^2}$ orbital ($^2B_{1g}$) as the ground state [23] and further confirm the covalent character of the M–L bond due to $g_{||}$ values were less than 2.3 [24]. The g_{eff} values of complex (**1b**) were observed at 1.396, which were further supportive of the covalent character of the M-L bond due to their values are less than 2.00277 [25]. The observed hyperfine constant parameters for complex (**1b**) were found in the following order: $A_{||} > A_{av} > A_{\perp}$ and the co-factor ($f_{||}$) values of the degree of geometrical distortion for these complexes were observed at 264.01 cm^{-1}. The obtained results are proposed that complex (**1b**) have distorted octahedral geometry [26]. The calculated G value for copper complex was

greater than 4, which attributed that the absence of interaction between Cu–Cu centres in the solid-state complex (**1b**) and the absence of half field signal at 1600 G consequent to the ΔMs = ± 2 transition rules out a Cu–Cu interaction [27,28]. The molecular orbital coefficient parameters α^2 (In-plane σ-bonding), β^2 (in-plane π–bonding) and γ^2 (out-plane π–bonding) for copper complex (**1b**) were measured from Kivelson and Neimann formulae (**Table S5)**. The observed α^2, β^2 and γ^2 results were in the range of 0.436, 0.832 and 0.627 respectively, which are indicated that the copper complex reveals the covalent character and also attributed that σ–bonding and π–bonding are entirely covalent character owing to their value are lower than 1.0 [29]. In addition, the obtained hyperfine interaction (K) value was found in the range of 0.300, which was less than 1.0 and it is more supportive to examine the covalent environment. The obtained orbital reduction factors $K_{\parallel}$ and $K_{\perp}$ values for copper complex (**1b**) were observed at 0.359 and 0.271 respectively, which suggest the presence of out-plane π–bonding in metal-ligand π–bonding [30]. The observed values of fermi contact hyperfine interaction term K_{fermi} for the copper complex were found at 0.232, which is further supported to measure the polarization generated by the uneven sharing of d-electron density on the inner core s-electron. All the observed results suggest that copper complex (**1b**) possesses distorted octahedral geometry and the results are also good agreed with electronic absorption data.

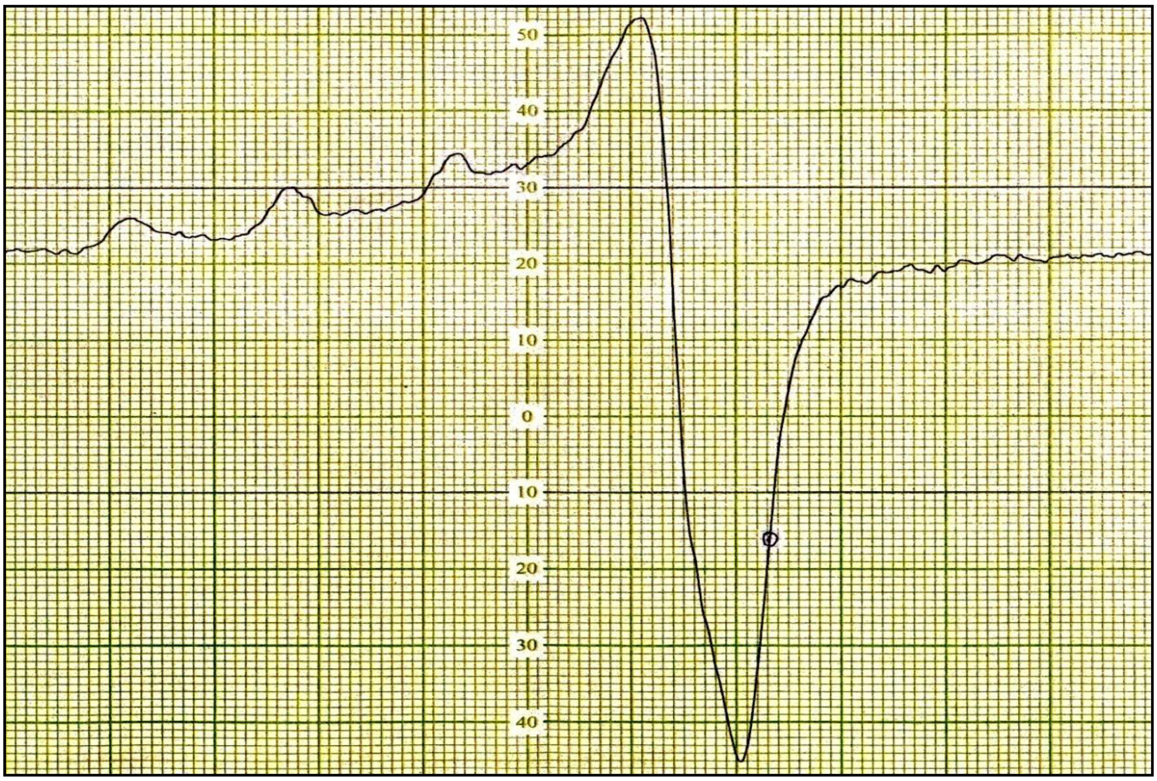

Fig. S10 EPR spectra of complex (**1b**) at liquid nitrogen temperature (77 K).

Table S5 The spin Hamiltonian parameters of complex (**1b**) at 77K.

	**g tensor**					**Hyperfine constant × 10^{-4} (cm^{-1})**				
	$g_{\|\|}$	$g_{\perp}$	g_{av}	g_{eff}		$A_{\|\|}$	$A_{\perp}$		A_{av}	
Complex (**1b**)	2.1552	2.0315	2.0727	1.396		81.66	25.75		44.3866	
	**Bonding Parameters**									
	G	$f_{\|\|}$ (cm^{-1})	α^2	β^2	γ^2	$K_{\|\|}^2$	$K_{\perp}^2$	K	K_{fermi}	μ_{eff}
	5.3051	264.01	0.4316	0.832	0.627	0.359	0.271	0.300	0.232	1.98

g_e = 2.00277, Microwave frequency (γ') = 9.114 × 10^9cycle/sec, Scan range = 2000-3000 G, 1G = 10^{-4} cm^{-1}, Electronic absorption E_{d-d} = 15,600 cm^{-1} for complex (**1b**), one-electron spin orbit coupling constant of free Cu(II) ion λ_0 = -828 cm^{-1}, Magnetic susceptibility (μ_{eff}) = 1.96 B.M, for complex (**1b**). Molecular orbital coefficient parameters α^2, β^2 and γ^2, Co-factor ($f_{||}$) value of degree of geometrical distortion; Ionic environment (K, K_{fermi}), Electron spin (S) = 1/2, G = Exchange interaction coupling constant. (**1**). $g_{\perp} = \frac{(3g_{av} - g_{||})}{2}$; (**2**). $K_{\perp} = \frac{(3A_{av} - A_{||})}{2}$; (**3**). $g_{av} = \frac{(g_{||} + 2g_{\perp})}{3}$; (**4**). $g_{eff} = \frac{(g_{||} + g_{\perp})}{3}$; (**5**). $A_{av} = \frac{(A_{||} + 2A_{\perp})}{3}$; (**6**). $G = \frac{(g_{||} - 2.00277)}{(g_{\perp} - 2.00277)}$; (**7**). $\alpha^2 = \frac{A_{||}}{P} + (g_{||} - 2.00277) + \frac{3}{7}(g_{\perp} - 2.00277) + 0.04$; (**8**). $\beta^2 = (g_{||} - 2.00277)(\frac{E_{d-d}}{-8\lambda_0 a^2})$; (**9**). $\gamma^2 = (g_{\perp} - 2.00277)(\frac{E_{d-d}}{-2\lambda_0 a^2})$; (**10**). $K_{||}^2 = (g_{||} - 2.00277)(\frac{E_{d-d}}{-8\lambda_0})$; (**11**). $K_{\perp}^2 = (g_{\perp} - 2.00277)(\frac{E_{d-d}}{-2\lambda_0})$; (**12**). $f_{||} = g_{||} / A_{||}$; (**13**). $K = \frac{(K_{||}^2 + 2K_{\perp}^2)}{3}$; (**14**). $K_{fermi} = \frac{A_{av}}{P\beta^2} + \frac{(g_{av} - 2.00277)}{\beta^2}$; (**15**). Free ion dipolar term $P = 2\Upsilon_{Cu}\beta_0 B_N (\gamma^{-3})$ = 0.036 cm^{-1}; (**16**). $\mu_{eff} = g_{av}[S(S+1)]^{1/2}$; Υ_{Cu} = magnetic moment value for copper, β_0 = Bohr Magneton, B_N = Nuclear Magneton, γ = the distance from the central nucleus to the electron.

3.1.10. Thermogravimetric Analysis (TGA)

The thermal stability of metal complexes is studied by controlling heating rates 10 °C per minute under nitrogen atmosphere. Thermogram of the mixed ligand complexes (**1b**–**2b**) is stable up to 180 –200 °C, the absence of lattice water as well as coordinated water. Generally in TG lattice water loses at low temperature region between 60–120 °C, where as coordinated water requires 120–200 °C. TGA thermograms of the mixed ligand complexes (**1b**–**2b**) have been recorded in the temperature range from 40 °C to 730 °C (**Figs. S11 & S12**). The stages of decomposition, temperature range, decomposition products, the obtained mass loss and calculated mass loss percentages of complexes (**1b**–**2b**) are summarized in **Table S6** [31, 32]. There are exposed three steps during the thermal decomposition of complexes. In the first step process at 40–180°C, the observed weight losses of decomposition of $[M^{II}(L)_2(phen)]$ complexes were found in the range of 10.70–10.72 % respectively, which corresponds to the loss of morpholine moieties (C_4H_8NO). In the second degradation stages, the weight losses were evaluated in the temperature range of 180–420 °C, which is further confirmed that the elimination of the rest aromatic part of ligand ($C_{13}H_9NO$) present in the complexes and they are found in the range of 24.03–24.46 % for complexes (**1b**–**2b**). Similarly, the obtained weight losses in the third degradation stage at 420–730 °C were pointed out in the range of 57.05–57.06 % elimination of ligand $C_{17}H_{17}N_2O$ (L) and co-ligand 1,10'–phenanthroline. $[C_{12}H_8N_2]$ in the mixed ligand complexes (**1b**–**2b**). The observed final percentage of rest products of metal oxide (MO) residue at above 730 °C was found in the range of 9.24–10.41 % for mixed ligand complexes (**1b**–**2b**). The thermal degradation steps of complexes (**1b**–**2b**) are shown in the flow chart (**Fig. S11**). Based on the above observations, the proposed structures of all complexes have been demonstrated in the experimental section.

Table S6 Thermal analysis of complexes (**1b**–**2b**) by TGA method.

**Complexes (M.W)**	**Step**	**Temperature range (°C)**	**% Weight loss [found (calcd)]**	**Assignment**
(**1b**) [Cu(L)$_2$(phen)] ($C_{46}H_{42}N_6O_4$)Cu (805.71)	I	40–180	10.72 (10.67)	C_4H_8NO
	II	180–420	24.46 (24.20)	$C_{13}H_9NO$
	III	420–730	57.05 (57.24)	$C_{17}H_{17}N_2O$, $C_{12}H_8N_2$
	Residue	>730	09.68 (09.87)	CuO
(**2b**) [Zn(L)$_2$(phen)] ($C_{46}H_{42}N_6O_4$)Zn (807.58)	I	40–180	10.70 (10.65)	C_4H_8NO
	II	180–420	24.03 (24.14)	$C_{13}H_9NO$
	III	420–730	57.06 (57.11)	$C_{17}H_{17}N_2O$, $C_{12}H_8N_2$
	Residue	>730	12.52 (12.46)	ZnO

M.W → Molecular Weight, TGA →Thermo Gravimetric Analysis.

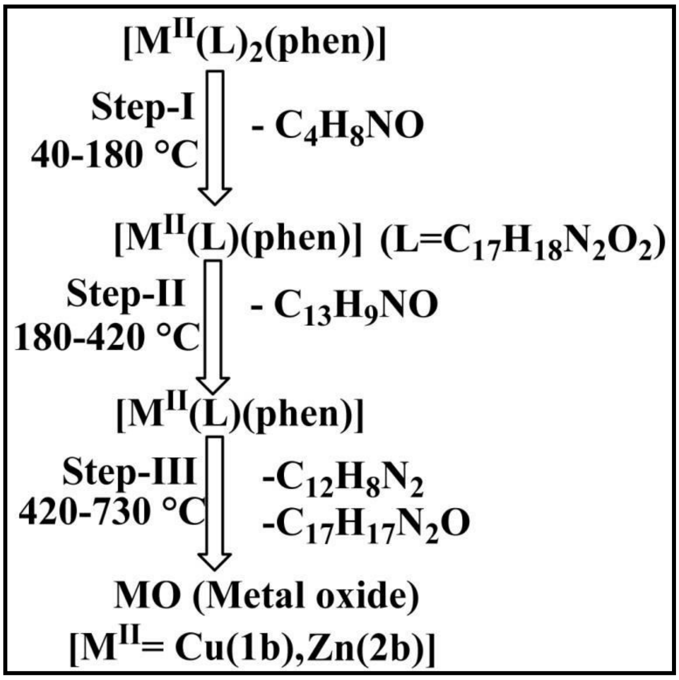

Fig. S11 The various stages for thermal decomposition of complexes (**1b**–**2b**) at temperature range of 40–730 °C.

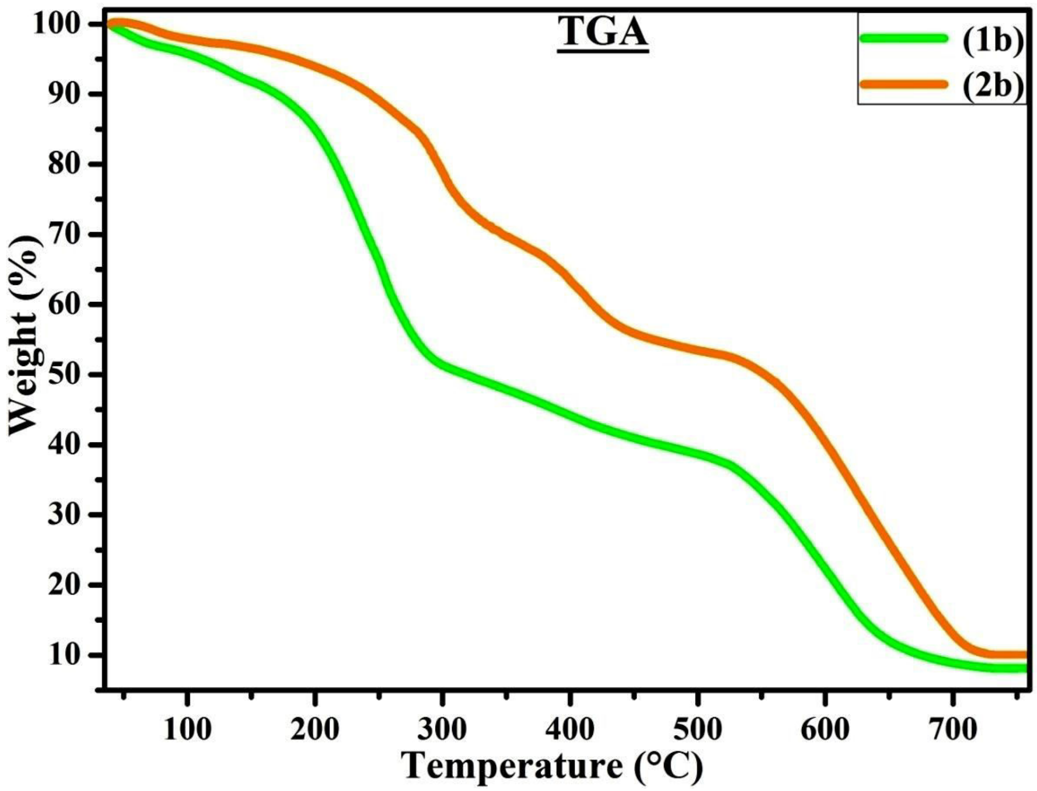

Fig. S12 TG plots of complexes (**1b–2b**) recorded under nitrogen atmosphere between the temperature range 40 and 750 °C at a heating rate of 20° C/min.

3.1.11. Single-Crystal X-ray Diffraction Analysis (SCXRD)

Schiff base ligand (**HL**) was obtained as a light yellow coloured single crystalline form in the presence of ethyl acetate medium by slow evaporation of the chloroform and ethanol mixture solution. Single-crystal XRD analysis of Schiff base ligand (**HL**) was performed to attain detailed information on molecular conformations in the solid-state. This analysis also proves the molecular structure and atom connectivity as shown in **Figs. S13a,b**. The information on the XRD data collection and structure refinements are certained in **Table S7** and hydrogen bonding geometry is given in **Table S8**. The crystallographic data were gathered at room temperature with the assistance of MoKα radiation in the wavelength of 0.71073 Å by Bruker AXS KAPPA APEX-2 diffractometer equipped with a graphite monochromator. The molecular structure of ligand was resolved by direct methods and refined by full-matrix least-

squares calculations using the SHELXL-2014 program [33]. The Schiff base ligand (**HL**) crystalline form was isomorphous in the monoclinic system and its space group P21/c with molecular formula $C_{17}H_{18}N_2O_2$ in the unit cell. The obtained results were also good agreed with spectral and analytical data. The bond length (Å) between carbon-carbon in the ligand (**HL**) was obtained as C(1)-C(2)→1.197(11), C(2)-C(3)→1.423(13), C(3)-C(4)→1.466(14), C(4)-C(5)→1.410(5), C(5)-C(6)→1.446(5), C(7)-C(8)→1.229(11), C(8)-C(9)→1.382(12), C(9)-C(10)→1.193(11), C(10)-C(11)→1.562(12), C(11)-C(12)→1.377(11), C(12)-C(13)→1.223(12), C(14)-C(15)→1.405(13) and C(16)-C(17)→1.348(13). The bond length (Å) between carbon-hydrogen was observed as C(2)-H(2), C(2)-H(2), C(3)-H(3), C(4)-H(4), C(5)-H(5), C(7)-H(7), C(9)-H(9), C(10)-H(10), C(12)-H(12), C(13)-H(13)→0.9300, C(14)-H (14A), C(14)-H (14B), C(15)-H (15A), C(15)-H (15B), C(16)-H (16A), C(16)-H (16B), C(17)-H (17A), C(17)-H (17B)→0.9700. Also, the bond lengths (Å) for carbon-nitrogen, carbon-oxygen and oxygen-hydrogen atoms were observed as C(6)-N(1)→1.215(10), C(7)-N(1)→1.360(11), C(11) N(2)→1.266(10), C(14)-N(2)→1.467(12), C(17)-N(2)→1.587(11), C(1)-O(1)→1.420(10), C(15)-O(2)→1.491(12), C(16)-O(2)→1.463(13) and O(1)-H(1O)→1.28(14). The bond angle (°) for carbon-carbon-carbon in the ligand (**HL**) was found as C(1)-C(2)-C(3)→105.2(8), C(2)-C(3)-C(4)→137.5(8), C(5)-C(4)-C(3)→120.9(8), C(4)-C(5)-C(6)→96.7(7), C(5)-C(6)-C(1)→139.6(7), C(7)-C(8)-C(9)→109.4(8), C(7)-C(8)-C(13)→122.4(9), C(9)-C(8)-C(13)→128.1(8), C(10)-C(9)-C(8)→107.1(9), C(9)-C(10)-C(11)→124.8(9), C(12)-C(11)-C(10)→128.3(8), C(13)-C(12)-C(11)→105.4(9), C(12)-C(13)-C(8)→126.3(9). Also, The bond angle (°) for carbon-carbon-oxygen, oxygen-carbon-hydrogen, nitrogen-carbon-carbon and carbon-nitrogen-carbon in the ligand (HL) was found as C(2)-C(1)-O(1)→105.8(8), O(1)-C(1)-C(6)→134.4(7), C(14)-C(15)-O(2)→116.6(8), O(2)-C(15)-H(15A)→108.1,O(2)-C(15)-H(15B)→108.1, C(17)-C(16)-O(2)→96.1(9), O(2)-C(16)-H(16A)→112.5, O(2)-C(16)-H(16B)→112.5, C(1)-O(1)-H(1O)→116(5), C(16)-O(2)-

C(15)→121.0(7), N(1)-C(6)-C(5)→106.2(7), N(1)-C(6)-C(1)→113.9(7), C(8)-C(7)-N(1)→127.1(9), C(6)-N(1)-C(7)→119.3(7), C(11)-N(2)-C(14)→99.3(7), C(11)-N(2)-C(17)→119.8(7), C(14)-N(2)-C(17)→127.9(7). The two phenyl rings are bridged by the iminic group (HC=N) in the molecular structure of ligand and its bond length (Å) of HC=N was found at 1.360(11) and its bond angle of C(6)=N(1)-C(7) around 119.3(7)°. Also, the bond angle of nitrogen in the sp^3 hybridized morpholine ring system [C(14)-N(2)-C(17)] was 127.9(7)°. The dihedral angle between these two phenyl rings was observed at 7.8(13)°, which demonstrates that both the phenyl rings are almost coplanar. However, the atoms O(1) slightly deviates from the least-quares plane of the phenyl ring [34]. Furthermore, the phenyl rings are stacked face-to-face in crystalline lattice leading to π–π interactions, which are further supported to assemble the molecules in the unit cell. The intermolecular interactions, especially classical and non-classical H-bonds are playing an essential role in the generation of the crystal and their physicochemical characteristics [35]. These weak intermolecular interactions and hydrogen bonding interactions can be classified and analyzed with graph-set nomenclature, which is more helpful in comparing the stability of the interactions [36]. However, the crystal packing is stabilized through intra-molecular hydrogen bonding interactions (O-H--N) and π–π interactions. The H-bonding dimensions are summarized in **Table S8** and the packing arrangement of molecules is represented in **Fig. S13b**. Moreover, the morpholine fragments are stabilized in the crystal packing by forming intermolecular hydrogen bonds C(2)-H(2)...O(2) between the morpholine ring oxygen O(2) and the hydrogen atom of phenyl ring [C(2)-H(2)] [37] and the packing may also be completed by hydrophobic van der Waals force attractions between the morpholine bearing ligands. In addition, the intramolecular hydrogen bonds O(1)-H...N between the hydroxyl group and iminic group, may lead to the further stabilization of the molecular structure of the ligand (**HL**) (**Table S8**). In addition, some other crystal data including structure refinement, atomic

coordinates, bond lengths [Å] and angles [°], equivalent isotropic displacement and anisotropic displacement parameters for the ligand (**HL**) are also summarized in the section (**Tables S8 – S13**).

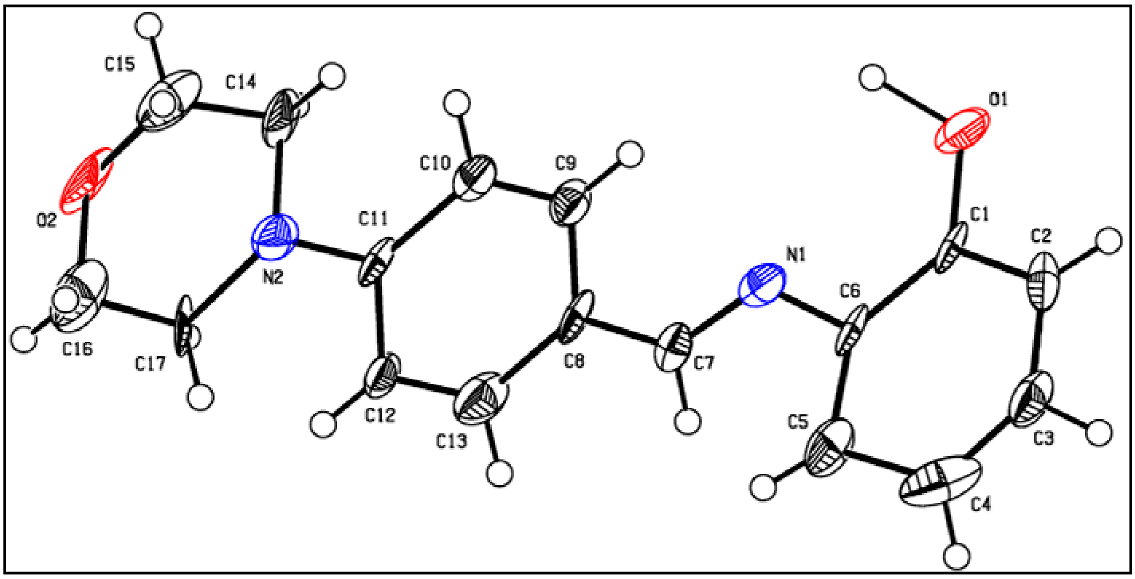

(a)

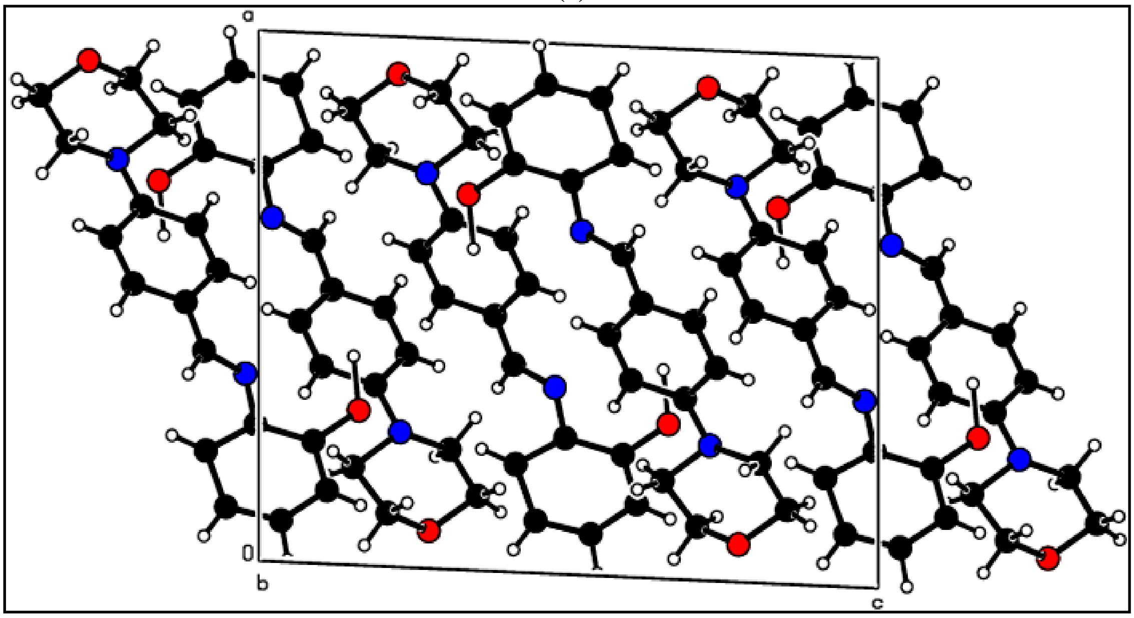

(b)

Fig.13 (**a**) ORTEP view of crystal structure of the ligand (**HL**) and (**b**) Unit cell packing diagram of ligand (**HL**).

Table S7 Crystal data and structure refinement for the ligand (**HL**).

Empirical formula	$C_{17}H_{18}N_2O_2$
Formula weight	282.33
Temperature	101 (2) K
Wavelength	0.71073 Å
Crystal system	Monoclinic
Space group	$P2_1/c$
Unit cell dimensions	a = 14.86 78 (12) Å, α = 90°<br>b = 7.5710 (6) Å, β = 93.197 (2)°<br>c = 12.3380 (8) Å, γ = 90°
Volume	1386.65 (18) $Å^3$
Z, calculated Density	4, 1.352 Mg/m^3
Absorption coefficient	0.090 mm^{-1}
F(000)	600
Crystal size	0.312 × 0.311 × 0.078 mm
Theta range for data collection	θ = 2.744 to 25.332°.
Limiting indices (Index ranges)	h = –17 → 17<br>k = –09 → 09<br>l = –14 → 14
Reflections collected	37050
Independent reflections	2505 [R(int) = 0.5296]
Completeness to Δ = 25.332°	98.8 %
Refinement method	Full-matrix least-squares on F2
Data / restraints / parameters	2505 / 0 / 218
Goodness-of-fit on F2	1.222
Final R indices [I > 2 σ (I)]	R1 = 0.1442, wR2 = 0.1293
R indices (all data)	R1 = 0.1002, wR2 = 0.0694
Largest diff. peak and hole	0.987 and –0.546 e.$Å^{-3}$

Table S8 Hydrogen bonds for the ligand (**HL**) (Å & °).

**D-H...A**	**d(D-H)**	**d(H...A)**	**d(D...A)**	**<(DHA)**
C(2)-H(2)...O(2)#1	0.93	2.53	3.255(12)	135.2

Symmetry transformations used to generate equivalent atoms: #1 x-1,-y+3/2,z+1/2

Table S9 Atomic coordinates ($\times 10^4$) and equivalent isotropic displacement parameters ($Å^2 \times 10^3$) for Schiff base ligand (**HL**). U(eq) is defined as one third of the trace of the orthogonalized U^{ij} tensor for the ligand (**HL**).

**Atoms**	**x**	**y**	**z**	**U(eq)**
C(1)	2302(6)	7693(10)	5879(6)	26(2)
C(2)	1389(7)	7688(11)	6100(6)	35(2)
C(3)	781(7)	7005(11)	5358(7)	41(3)
C(4)	989(7)	6332(12)	4470(7)	58(3)
C(5)	2036(6)	6333(11)	4147(5)	43(3)
C(6)	2575(6)	6999(10)	4947(5)	26(2)
C(7)	3919(7)	6180(11)	4117(6)	30(2)
C(8)	4821(6)	6283(11)	3817(6)	31(2)
C(9)	5443(7)	7397(10)	4336(6)	31(2)
C(10)	6282(7)	7544(11)	3989(6)	34(2)
C(11)	6589(6)	6600(10)	3118(6)	27(2)
C(12)	5979(7)	5463(11)	2607(6)	32(2)
C(13)	5122(8)	5356(12)	2971(7)	44(3)
C(14)	8153(7)	7373(13)	3479(6)	41(2)
C(15)	8989(8)	7814(13)	2922(8)	56(3)
C(16)	8596(8)	6145(14)	1467(9)	61(3)
C(17)	7742(7)	5617(12)	1916(6)	35(2)
N(1)	3516(6)	7145(9)	4780(5)	34(2)
N(2)	7452(6)	6836(9)	2719(5)	37(2)
O(1)	2918(5)	8375(9)	6614(4)	51(2)
O(2)	9292(5)	6503(10)	2254(5)	64(2)

Table S10 Bond lengths [Å] and angles [°] for the ligand (**HL**).

Positions of atoms	Bond lengths [Å] and bond angles [°]
C(1)-C(2)	1.197(11)
C(1)-O(1)	1.420(10)
C(1)-C(6)	1.550(12)
C(2)-C(3)	1.423(13)
C(2)-H(2)	0.93
C(3)-C(4)	1.466(14)
C(3)-H(3)	0.93
C(4)-C(5)	1.410(5)
C(4)-H(4)	0.93
C(5)-C(6)	1.446(5)
C(5)-H(5)	0.93
C(6)-N(1)	1.215(10)
C(7)-C(8)	1.229(11)
C(7)-N(1)	1.360(11)
C(7)-H(7)	0.93
C(8)-C(9)	1.382(12)
C(8)-C(13)	1.521(13)
C(9)-C(10)	1.193(11)
C(9)-H(9)	0.93
C(10)-C(11)	1.562(12)
C(10)-H(10)	0.93
C(11)-N(2)	1.266(10)
C(11)-C(12)	1.377(11)
C(12)-C(13)	1.223(12)
C(12)-H(12)	0.93
C(13)-H(13)	0.93
C(14)-C(15)	1.405(13)
C(14)-N(2)	1.467(12)
C(14)-H(14A)	0.97
C(14)-H(14B)	0.97
C(15)-O(2)	1.491(12)
C(15)-H(15A)	0.97
C(15)-H(15B)	0.97
C(16)-C(17)	1.348(13)
C(16)-O(2)	1.463(13)
C(16)-H(16A)	0.97
C(16)-H(16B)	0.97
C(17)-N(2)	1.587(11)
C(17)-H(17A)	0.97
C(17)-H(17B)	0.97
O(1)-H(1O)	1.28(14)

C(2)-C(1)-O(1)	105.8(8)
C(2)-C(1)-C(6)	119.8(8)
O(1)-C(1)-C(6)	134.4(7)
C(1)-C(2)-C(3)	105.2(8)
C(1)-C(2)-H(2)	127.4
C(3)-C(2)-H(2)	127.4
C(2)-C(3)-C(4)	137.5(8)
C(2)-C(3)-H(3)	111.3
C(4)-C(3)-H(3)	111.3
C(5)-C(4)-C(3)	120.9(8)
C(5)-C(4)-H(4)	119.5
C(3)-C(4)-H(4)	119.5
C(4)-C(5)-C(6)	96.7(7)
C(4)-C(5)-H(5)	131.6
C(6)-C(5)-H(5)	131.6
N(1)-C(6)-C(5)	106.2(7)
N(1)-C(6)-C(1)	113.9(7)
C(5)-C(6)-C(1)	139.6(7)
C(8)-C(7)-N(1)	127.1(9)
C(8)-C(7)-H(7)	116.5
N(1)-C(7)-H(7)	116.5
C(7)-C(8)-C(9)	109.4(8)
C(7)-C(8)-C(13)	122.4(9)
C(9)-C(8)-C(13)	128.1(8)
C(10)-C(9)-C(8)	107.1(9)
C(10)-C(9)-H(9)	126.5
C(8)-C(9)-H(9)	126.5
C(9)-C(10)-C(11)	124.8(9)
C(9)-C(10)-H(10)	117.6
C(11)-C(10)-H(10)	117.6
N(2)-C(11)-C(12)	106.8(8)
N(2)-C(11)-C(10)	124.8(8)
C(12)-C(11)-C(10)	128.3(8)
C(13)-C(12)-C(11)	105.4(9)
C(13)-C(12)-H(12)	127.3
C(11)-C(12)-H(12)	127.3
C(12)-C(13)-C(8)	126.3(9)
C(12)-C(13)-H(13)	116.9
C(8)-C(13)-H(13)	116.9
C(15)-C(14)-N(2)	92.2(8)
C(15)-C(14)-H(14A)	113.3
N(2)-C(14)-H(14A)	113.3
C(15)-C(14)-H(14B)	113.3
N(2)-C(14)-H(14B)	113.3
H(14A)-C(14)-H(14B)	110.6
C(14)-C(15)-O(2)	116.6(8)

C(14)-C(15)-H(15A)	108.1
O(2)-C(15)-H(15A)	108.1
C(14)-C(15)-H(15B)	108.1
O(2)-C(15)-H(15B)	108.1
H(15A)-C(15)-H(15B)	107.3
C(17)-C(16)-O(2)	96.1(9)
C(17)-C(16)-H(16A)	112.5
O(2)-C(16)-H(16A)	112.5
C(17)-C(16)-H(16B)	112.5
O(2)-C(16)-H(16B)	112.5
H(16A)-C(16)-H(16B)	110
C(16)-C(17)-N(2)	114.0(8)
C(16)-C(17)-H(17A)	108.7
N(2)-C(17)-H(17A)	108.7
C(16)-C(17)-H(17B)	108.7
N(2)-C(17)-H(17B)	108.7
H(17A)-C(17)-H(17B)	107.6
C(6)-N(1)-C(7)	119.3(7)
C(11)-N(2)-C(14)	99.3(7)
C(11)-N(2)-C(17)	119.8(7)
C(14)-N(2)-C(17)	127.9(7)
C(1)-O(1)-H(1O)	116(5)
C(16)-O(2)-C(15)	121.0(7)

Symmetry transformations used to generate equivalent atoms

Table S11 Anisotropic displacement parameters (Å^2 × 10^3) for the ligand (**HL**). The anisotropic displacement factor exponent takes the form: $-2\pi^2[h^2a^{*2}U^{11} + ... + 2 h k a^* b^* U^{12}]$.

Atoms	U^{11}	U^{22}	U^{33}	U^{23}	U^{13}	U^{12}
C(1)	8(3)	24(3)	48(4)	6(3)	10(3)	-1(2)
C(2)	32(6)	30(5)	44(6)	1(4)	23(5)	1(4)
C(3)	19(5)	38(6)	66(7)	-5(5)	10(5)	1(4)
C(4)	44(7)	29(6)	99(9)	-1(6)	-32(6)	-2(5)
C(5)	19(5)	48(6)	62(7)	3(5)	1(4)	9(4)
C(6)	8(3)	24(3)	48(4)	6(3)	10(3)	-1(2)
C(7)	18(5)	36(5)	36(5)	2(4)	5(4)	1(4)
C(8)	7(4)	34(5)	53(6)	5(5)	8(4)	3(4)
C(9)	24(5)	25(5)	44(6)	-4(4)	8(4)	-4(4)
C(10)	24(5)	38(5)	42(6)	-4(5)	5(4)	-8(4)
C(11)	8(4)	25(5)	47(6)	5(4)	5(4)	0(4)
C(12)	15(5)	34(5)	47(6)	-11(5)	9(4)	-3(4)
C(13)	26(6)	37(5)	70(7)	-9(5)	0(5)	1(4)
C(14)	14(5)	53(6)	56(6)	-4(5)	11(4)	8(4)
C(15)	24(6)	47(7)	97(9)	-1(6)	-6(6)	1(5)
C(16)	31(6)	47(7)	108(10)	-5(6)	14(6)	10(5)
C(17)	21(5)	38(5)	49(6)	-4(5)	28(4)	5(4)
N(1)	24(4)	38(5)	41(5)	-3(4)	-1(3)	-7(3)
N(2)	21(4)	40(5)	50(5)	-14(4)	1(4)	4(3)
O(1)	23(4)	58(4)	71(5)	-22(4)	0(3)	-13(3)
O(2)	7(3)	74(5)	111(6)	-14(5)	8(4)	1(3)

Table S12 Hydrogen coordinates (× 10^4) and isotropic displacement parameters (Å^2 × 10^3) for the ligand (**HL**).

Atoms	x	y	z	U(eq)
H(2)	1133	8053	6642	42
H(3)	52	6977	5467	49
H(4)	422	5898	4114	70
H(5)	2295	6012	3599	52
H(7)	3463	5353	3862	36
H(9)	5245	7933	4858	37
H(10)	6792	8274	4257	41
H(12)	6178	4895	2095	38
H(13)	4604	4640	2704	53
H(14A)	7874	8346	3800	49
H(14B)	8339	6442	3886	49
H(15A)	9622	8078	3297	68
H(15B)	8784	8856	2604	68
H(16A)	8442	7165	1112	74
H(16B)	8880	5247	1095	74
H(17A)	7886	4473	2148	42
H(17B)	7122	5540	1503	42
H(1O)	3940(110)	8420(160)	6530(80)	110(40)

Table S13 Torsion angles [°] for for the ligand (**HL**).

Positions of atoms	Torsion angles [°]
O(1)-C(1)-C(2)-C(3)	179.9(6)
C(6)-C(1)-C(2)-C(3)	-1.0(11)
C(1)-C(2)-C(3)-C(4)	1.4(15)
C(2)-C(3)-C(4)-C(5)	-3.2(16)
C(3)-C(4)-C(5)-C(6)	3.5(11)
C(4)-C(5)-C(6)-N(1)	-177.9(7)
C(4)-C(5)-C(6)-C(1)	-4.6(13)
C(2)-C(1)-C(6)-N(1)	176.6(9)
O(1)-C(1)-C(6)-N(1)	-4.7(13)
C(2)-C(1)-C(6)-C(5)	3.8(15)
O(1)-C(1)-C(6)-C(5)	-177.6(9)
N(1)-C(7)-C(8)-C(9)	7.8(13)
N(1)-C(7)-C(8)-C(13)	-169.2(8)
C(7)-C(8)-C(9)-C(10)	-175.5(9)
C(13)-C(8)-C(9)-C(10)	1.3(13)
C(8)-C(9)-C(10)-C(11)	-0.9(13)
C(9)-C(10)-C(11)-N(2)	175.4(10)
C(9)-C(10)-C(11)-C(12)	-0.3(15)
N(2)-C(11)-C(12)-C(13)	-175.1(8)
C(10)-C(11)-C(12)-C(13)	1.2(13)
C(11)-C(12)-C(13)-C(8)	-0.8(13)
C(7)-C(8)-C(13)-C(12)	176.0(10)
C(9)-C(8)-C(13)-C(12)	-0.4(15)
N(2)-C(14)-C(15)-O(2)	-49.9(10)
O(2)-C(16)-C(17)-N(2)	46.5(10)
C(5)-C(6)-N(1)-C(7)	-23.2(11)
C(1)-C(6)-N(1)-C(7)	161.6(7)
C(8)-C(7)-N(1)-C(6)	173.3(9)
C(12)-C(11)-N(2)-C(14)	-156.1(7)
C(10)-C(11)-N(2)-C(14)	27.4(10)
C(12)-C(11)-N(2)-C(17)	-11.9(10)
C(10)-C(11)-N(2)-C(17)	171.6(7)
C(15)-C(14)-N(2)-C(11)	-171.9(7)
C(15)-C(14)-N(2)-C(17)	48.1(11)
C(16)-C(17)-N(2)-C(11)	171.0(9)
C(16)-C(17)-N(2)-C(14)	-56.0(13)
C(17)-C(16)-O(2)-C(15)	-61.2(11)
C(14)-C(15)-O(2)-C(16)	72.9(12)

Symmetry transformations used to generate equivalent atoms

References

[1] K. Sakthikumar, J. Dhaveethu Raja, R. Vijay Solomon, M. Sankarganesh, Density functional theory molecular modelling, DNA interactions, antioxidant, antimicrobial, anticancer and biothermodynamic studies of bioactive water-soluble mixed ligand complexes, J. Biomol. Struct.37 (2018) 2498–2514, http://dx.doi.org/10.1080/07391102.2018.1492970.

[2] K. Sakthikumar, R.V. Solomon, J.D. Raja, Spectro-electrochemical assessments of DNA/BSA interactions, cytotoxicity, radical scavenging and pharmacological implications of biosensitive and biologically active morpholine-based metal(II) complexes: a combined experimental and computational investigation, RSC Advances. 9 (2019) 14220–14241, http://dx.doi.org/10.1039/c8ra09218d.

[3] M. Asadi, N. Savaripoor, Z. Asadi, M.H. Ghatee, F. Moosavi, R. Yousefi, M. Jamshidi, Synthesis and characterization of some new Schiff base complexes of group 13 elements, ab initio studies, cytotoxicity and reaction with hydrogen peroxide, Spectrochim. Acta A Mol. Biomol. Spectrosc.101 (2013) 394–399, https://doi.org/10.1016/j.saa.2012.09.007/.

[4] F. Najóczki, M. Szabó, N. Lihi, A. Udvardy, I. Fábián, Synthesis and Characterization of 1,10-Phenanthroline-mono-N-oxides, Molecules, 26, (2021) 3632, https://doi.org/10.3390/molecules26123632.

[5] W.Z. Shen, G. Trötscher-Kaus, B. Lippert, ^{1}H NMR spectroscopic identification of binding modes of 2,2′-bipyridine ligands in complexes of square-planar d8 metal ions, Dalton Trans. 39 (2009) 8203, https://doi.org/10.1039/b904173g.

[6] X. Li, H. Wanyan, W. Dong, R. Yang, Synthesis of novel mixed-ligand complexes of lanthanide ions with 1,4-bis-(1′-phenyl-3′-methyl-5′-pyrazolone-4′)-1,4-butanedione and 1,10-phenanthroline and their UV, IR, ^{1}H NMR, fluorescence and thermal analysis studies, Polyhedron. 9 (1990)2285–2291, https://doi.org/10.1016/s0277-5387(00)86955-7.

[7] S. Sirinavin, C. Techasaensiri, S. Benjaponpitak, R. Pornkul, M. Vorachit, Invasive Chromobacteriumviolaceum Infection in Children, J. Pediatr. Infect. Dis. 24 (2005) 559–561, https://doi.org/10.1097/01.inf.0000164761.81491.3f.

[8] D.M. Boghaei, M. Gharagozlou, Spectral characterization of novel ternary zinc(II) complexes containing 1,10-phenanthroline and Schiff bases derived from amino acids and salicylaldehyde-5-sulfonates, Spectrochim. Acta A Mol. Biomol. Spectrosc. 67 (2007) 944–949, https://doi.org/10.1016/j.saa.2006.09.012.

[9] S. Srinivasan, J. Annaraj, PR. Athappan, Spectral and redox studies on mixed ligand complexes of cobalt(III) phenanthroline/bipyridyl and benzoylhydrazones, their DNA binding and antimicrobial activity, J. Inorg. Biochem. 99, (2005) 876–882, https://doi.org/10.1016/j.jinorgbio.2005.01.002.

[10] Y.M. Song, Q. Wu, P.J. Yang, N.N. Luan, L.F. Wang, Y.M. Liu, DNA Binding and cleavage activity of Ni(II) complex with all-trans retinoic acid, J. Inorg. Biochem. 100 (2006) 1685–1691, http://dx.doi.org/10.1016/j.jinorgbio.2006.06.00.

[11] K. Sakthikumar, J.D. Raja, M. Sankarganesh, J. Rajesh, Antimicrobial, Antioxidant and DNA Interaction Studies of Water-soluble Complexes of Schiff Base Bearing Morpholine Moiety, Indian J. Pharm. Sci. 80 (2018) 723–738, http://dx.doi.org/10.4172/pharmaceutical-sciences.1000413.

[12] K.L. V. Joseph, J. Lim, A. Anthonysamy, H. Kim, W. Choi, J.K. Kim, Squaraine-sensitized composite of a reduced graphene oxide/TiO2 photocatalyst: π–π stacking as a new method of dye anchoring, J. Mater. Chem A. 3 (2015) 232–239, https://doi.org/10.1039/c4ta04313h.

[13] R.J. Allenbaugh, J.R. Zachary, J.R. Williams, A.L. Shaw, J.D. Bryson, A.N. Underwood, E.E. O’Donnell, Mechanochemical synthesis of zinc and palladium complexes of dialkyl 2,2′-bipyridine-4,4′-dicarboxylate and analysis of solid-state reaction kinetics,

Inorganica.Chimica Acta. 477 (2018) 102–108, http://dx.doi.org/10.1016/j.ica.2018.03.004.

[14] S. Utsuno, Y. Yoshikawa, A. Tatehata, H. Yamatera, ^{13}C NMR Spectra of Series of Bis(2,2′-bipyridine)cobalt(III)and Bis(1,10-phenanthroline)cobalt(III) Complexes, Bull. Chem. Soc. Jpn, 54 (1981) 1814–1817, https://doi.org/10.1246/bcsj.54.1814.

[15] L. Pazderski, T. Pawlak, J. Sitkowski, L. Kozerski, E. Szłyk, ^{1}H NMR assignment corrections and1H,13C,15N NMR coordination shifts structural correlations in Fe(II), Ru(II) and Os(II) cationic complexes with 2,2′-bipyridine and 1,10-phenanthroline, Magn. Reson. Chem, 0, (2010), https://doi.org/10.1002/mrc.2600.

[16] E.S. Aazam, A.F. EL Husseiny, H.M. Al-Amri, Synthesis and photoluminescent properties of a Schiff-base ligand and its mononuclear Zn(II), Cd(II), Cu(II), Ni(II) and Pd(II)metal complexes, Arab. J. Chem. 5 (2012) 45–53, http://dx.doi.org/10.1016/j.arabjc.2010.07.022.

[17] N. Raman, S. Ali, D. Raja, Designing, synthesis and spectral characterization of Schiff base transition metal complexes: DNA cleavage and antimicrobial activity studies, J. Serb. Chem. Soc. 73 (2008) 1063–1071l, http://dx.doi.org/10.2298/jsc0811063r.

[18] Y. Li, Z.Y. Yang, Z.C. Liao, Z.C. Han, Z.C. Liu, Synthesis, crystal structure, DNA binding properties and antioxidant activities of transition metal complexes with 3-carbaldehyde-chromone semicarbazone, Inorg. Chem. Commun. 13 (2010)1213–1216, https://doi.org/10.1016/j.inoche.2010.07.005.

[19] G.D. Dimitrov, M.S. Atanassova, Synthesis and Spectroscopic Characterization of a Complex of 1, 10-Phenanthroline with Magnesium, Z. anorg. allg. Chem. 629 (2003) 12–14, https://doi.org/10.1002/zaac.200390005.

[20] M. Shakir, A. Abbasi, M. Azam, A.U. Khan, Synthesis, spectroscopic studies and crystal structure of the Schiff base ligand L derived from condensation of 2-

thiophenecarboxaldehyde and 3,3′-diaminobenzidine and its complexes with Co(II), Ni(II), Cu(II), Cd(II) and Hg(II): Comparative DNA binding studies of L and its Co(II), Ni(II) and Cu(II) complexes, Spectrochim. Acta A Mol. Biomol. 79 (2011) 1866–1875, http://dx.doi.org/10.1016/j.saa.2011.05.077.

[21] A.B.PLever, Electronic Spectra of d^n ions in Inorganic Electronic Spectroscopy, second ed., Elsevier, Amsterdam, Netherlands, (1984) 1-863.

[22] P.K. Panchal, H.M. Parekh, P.B. Pansuriya, M.N. Patel, Bactericidal activity of different oxovanadium(IV) complexes with schiff bases and application of chelation theory, J. Enzyme Inhib. Med. Chem. 21 (2006) 203-209, https://doi.org/10.1080/14756360500535229.

[23] M.B. Halli, R.B. Sumathi, M. Kinni, Synthesis, spectroscopic characterization and biological evaluation studies of Schiff's base derived from naphthofuran-2-carbohydrazide with 8-formyl-7-hydroxy-4-methyl coumarin and its metal complexes Spectrochim. Acta A Mol. Biomol. 99 (2012) 46–56, http://dx.doi.org/10.1016/j.saa.2012.08.089.

[24] D. Kivelson, R. Neiman, ESR Studies on the Bonding in Copper Complexes, J. Chem. Phy. 35 (1961) 149–155, http://dx.doi.org/10.1063/1.1731880.

[25] A. Syamal, Some improper terms in coordination chemistry, J. Chem. Edu. 62 (1985) 143, http://dx.doi.org/10.1021/ed062p143.

[26] D. E. Nikles, M. J. Powers, F. L. Urbach, Copper(II) complexes with tetradentate bis(pyridyl)-dithioether and bis(pyridyl)-diamine ligands. Effect of thio ether donors on the electronic absorption spectra, redox behavior, and EPR parameters of copper(II) complexes, Inorg. Chem. 22, no. 22 (1983) 3210–3217, https://doi.org/10.1021/ic00164a009.

[27] B.J. Hathaway, D.E. Billing, The electronic properties and stereochemistry of mono-nuclear complexes of the copper(II) ion, Coord. Chem. Rev. 5 (1970) 143–207, http://dx.doi.org/10.1016/s0010-8545(00)80135-6.

[28] A. L. Sharma, I. O. Singh, M. A. Singh, H. R. Singh, R. M. Kadam, M. K. Bhide, M. D. Sastry, EPR studies on dichloromono(1-phenylamidino-*o*-alkylurea)copper(II) complexes. Evidence for field induced partial ordering in the solid state and some unusual features in solution, Transit. Met. Chem. 26 (2001) 532–537, https://doi.org/10.1023/a:1011063631998.

[29] Y.Z. Tang, G.X. Wang, Q. Ye, R.G. Xiong, R.X. Yuan, Heterometallic Tetrazole Coordination Polymer Formed through 2 + 3 Cycloaddition Reaction between Inorganic Complexes in the Presence of Lewis Acid, Cryst. Growth Des. 7 (2007) 2382–2386, http://dx.doi.org/10.1021/cg070528g.

[30] B.J. Hathaway, A.A.G. Tomlinson, Copper(II) ammonia complexes, Coord. Chem. Rev. 5 (1970) 1–43, http://dx.doi.org/10.1016/s0010-8545(00)80073-9.

[31] W. Gurnule, P. Rahangdale, L. Paliwal, R. Kharat, Synthesis, characterization and ion-exchange properties of 4-hydroxyacetophenone, biuret and formaldehyde terpolymer resins, React. Funct. Polym. 55 (2003) 255–265, http://dx.doi.org/10.1016/s1381-5148(03)00016-6.

[32] P.P. Kalbende, M.V. Tarase, A.B. Zade, Preparation, Characterization, and Thermal Degradation Studies of p-Nitrophenol-Based Copolymer, J. Chem. 2013 (2013) 1–9, https://doi.org/10.1155/2013/846327.

[33] M.G. Miguel, S. Nunes, S.A. Dandlen, A.M. Cavaco, M.D. Antunes, Phenols, flavonoids and antioxidant activity of aqueous and methanolic extracts of propolis (Apis mellifera L.) from Algarve, South Portugal, Food. Sci. Tech (Campinas). 34 (2014) 16–23, http://dx.doi.org/10.1590/s0101-20612014000100002.

[34] Boora, E. Chirisa, S. Mukanganyama, Evaluation of Nitrite Radical Scavenging Properties of Selected Zimbabwean Plant Extracts and Their Phytoconstituents, J. Food Proc. 2014 (2014) 1–7, http://dx.doi.org/10.1155/2014/918018.

[35] S. Shujah, N. Khalid, S. Ali, Homobimetallic organotin(IV) complexes with succinohydrazide Schiff base: Synthesis, spectroscopic characterization and biological screening, Russ. J. Gen. Chem. 87 (2017) 515–522, http://dx.doi.org/10.1134/s1070363217030227.

[36] M. Balouiri, M. Sadiki, S.K. Ibnsouda, Methods for in vitro evaluating antimicrobial activity: A review, J. Pharm. Analysis. 6 (2016) 71–79, http://dx.doi.org/10.1016/j.jpha.2015.11.005.

[37] I. Ali, W.A. Wani, K. Saleem, M.F. Hsieh, Anticancer metallo drugs of glutamic acid sulphonamides: in silico, DNA binding, hemolysis and anticancer studies, RSC Adv. 4 (2014) 29629–29641, http://dx.doi.org/10.1039/c4ra02570a.

……………………………………………………………………………………

Supplementary Figures

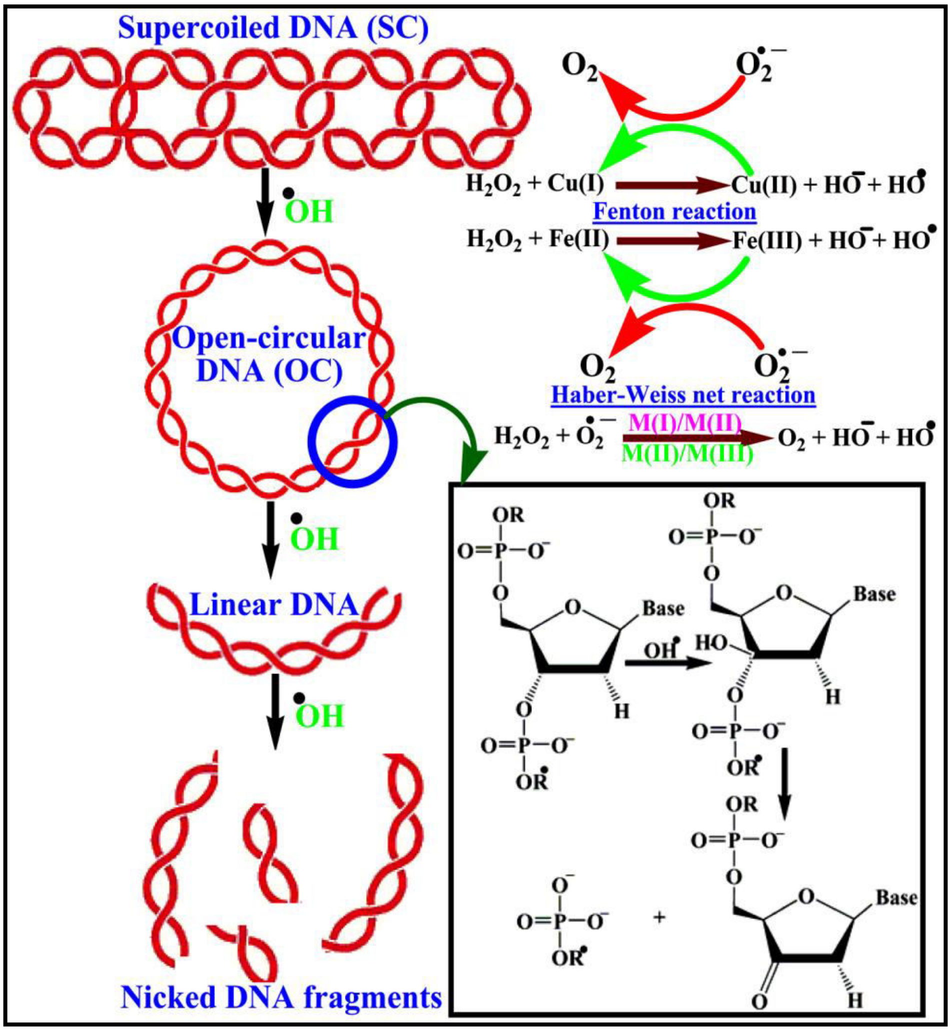

Fig. S14. Fenton and Haber-Weiss mechanisms for DNA cleavage in the H_2O_2 environment.

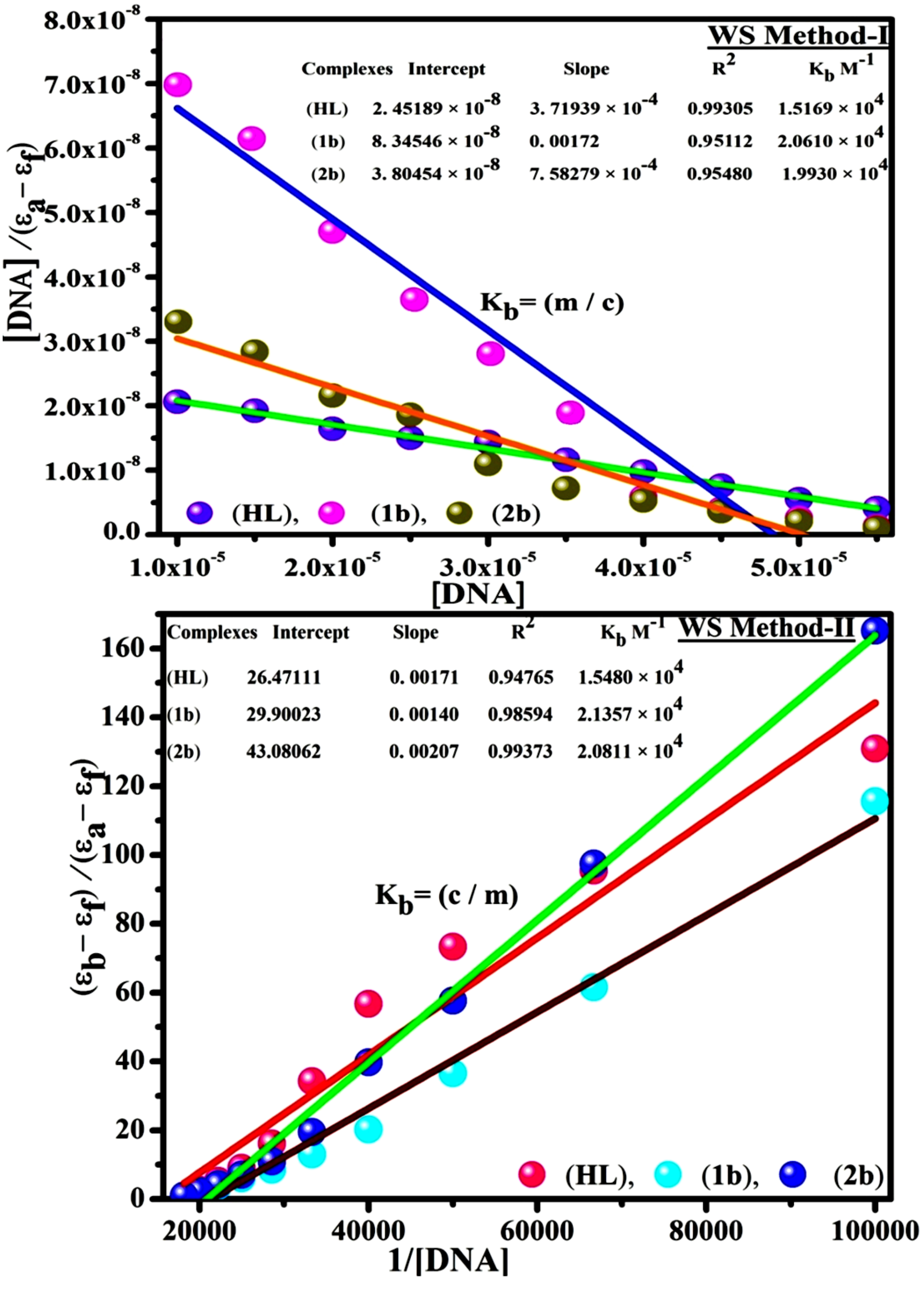

Fig.S15. Linear plots of {[DNA] / ($\varepsilon_a - \varepsilon_f$)} *vs* [DNA] and {($\varepsilon_b - \varepsilon_f$)/ ($\varepsilon_a - \varepsilon_f$)} *vs* [DNA] M^{-1} by Wolfe–Shimmer methods (I & II) for the estimation of the intrinsic DNA binding constants (K_b) for ligand (**HL**) and mixed ligand complexes (**1b**–**2b**).

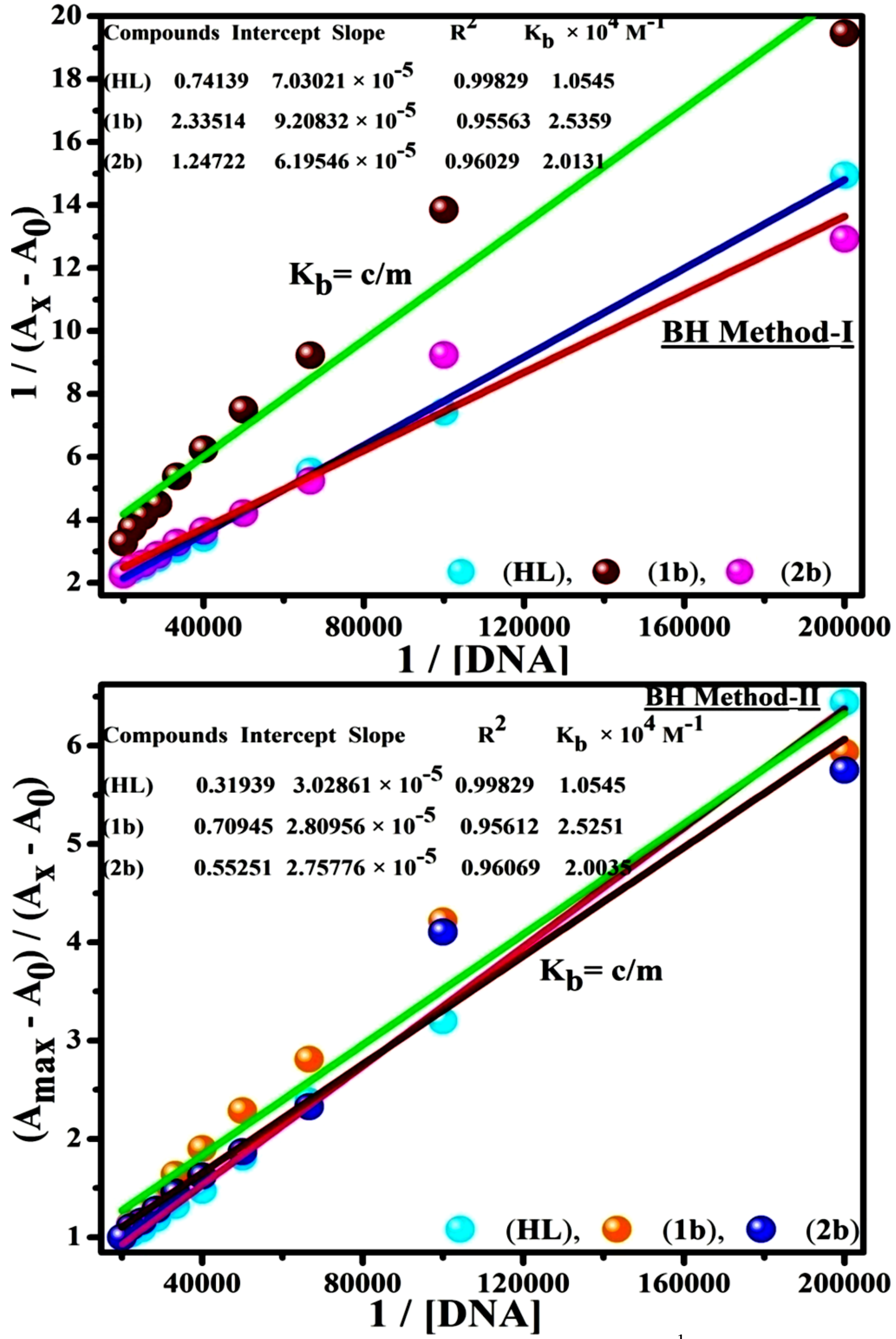

Fig.16. Linear plots of [1 / ($A_x - A_0$)] *vs* {1 / [DNA]} M^{-1} and [($A_{max} - A_0$) / ($A_x - A_0$)] *vs* {1 / [DNA]} M^{-1} by Benesi-Hildebrand (I & II) methods for the estimation of the intrinsic DNA binding constants (K_b) for ligand (**HL**) and complexes (**1b–2b**).

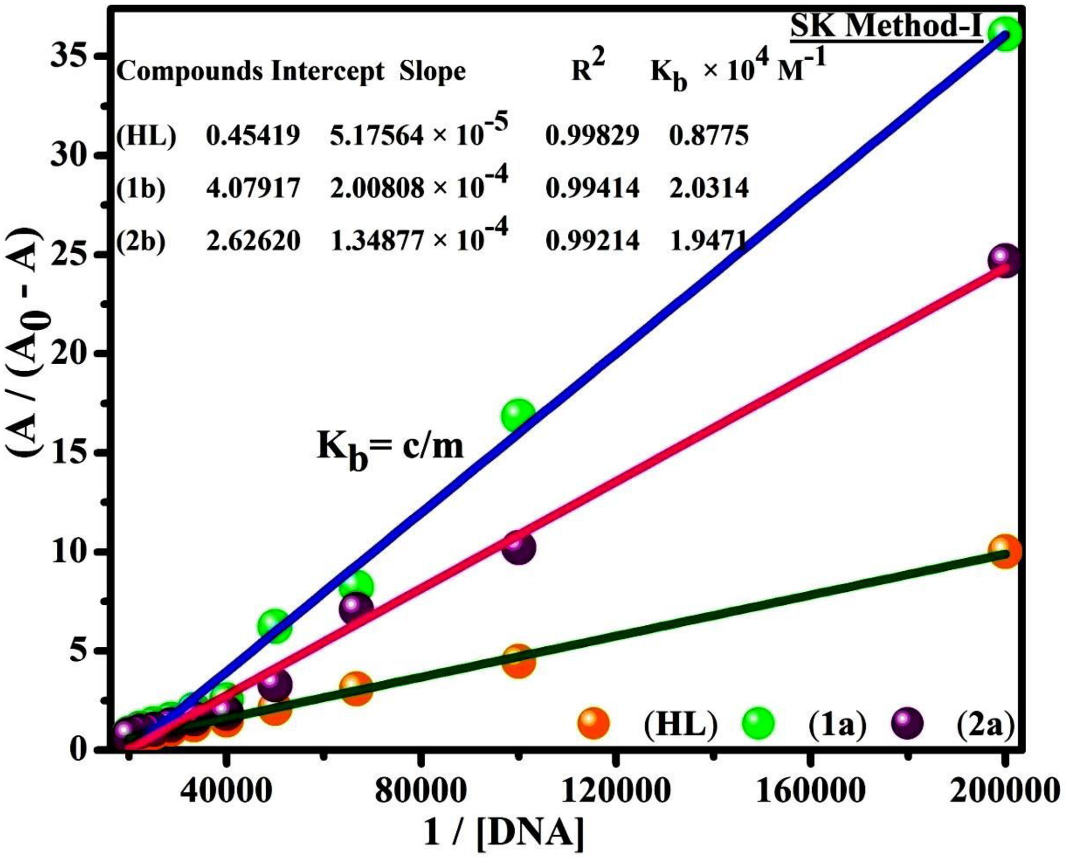

Fig.S17. Linear plot of [(A / (A_0 – A)] *vs* {1 / [DNA]} M^{-1} by Sakthi–Kruase-I method for the estimation of the intrinsic DNA binding constants (K_b) for ligand (**HL**) and complexes (**1b**–**2b**).

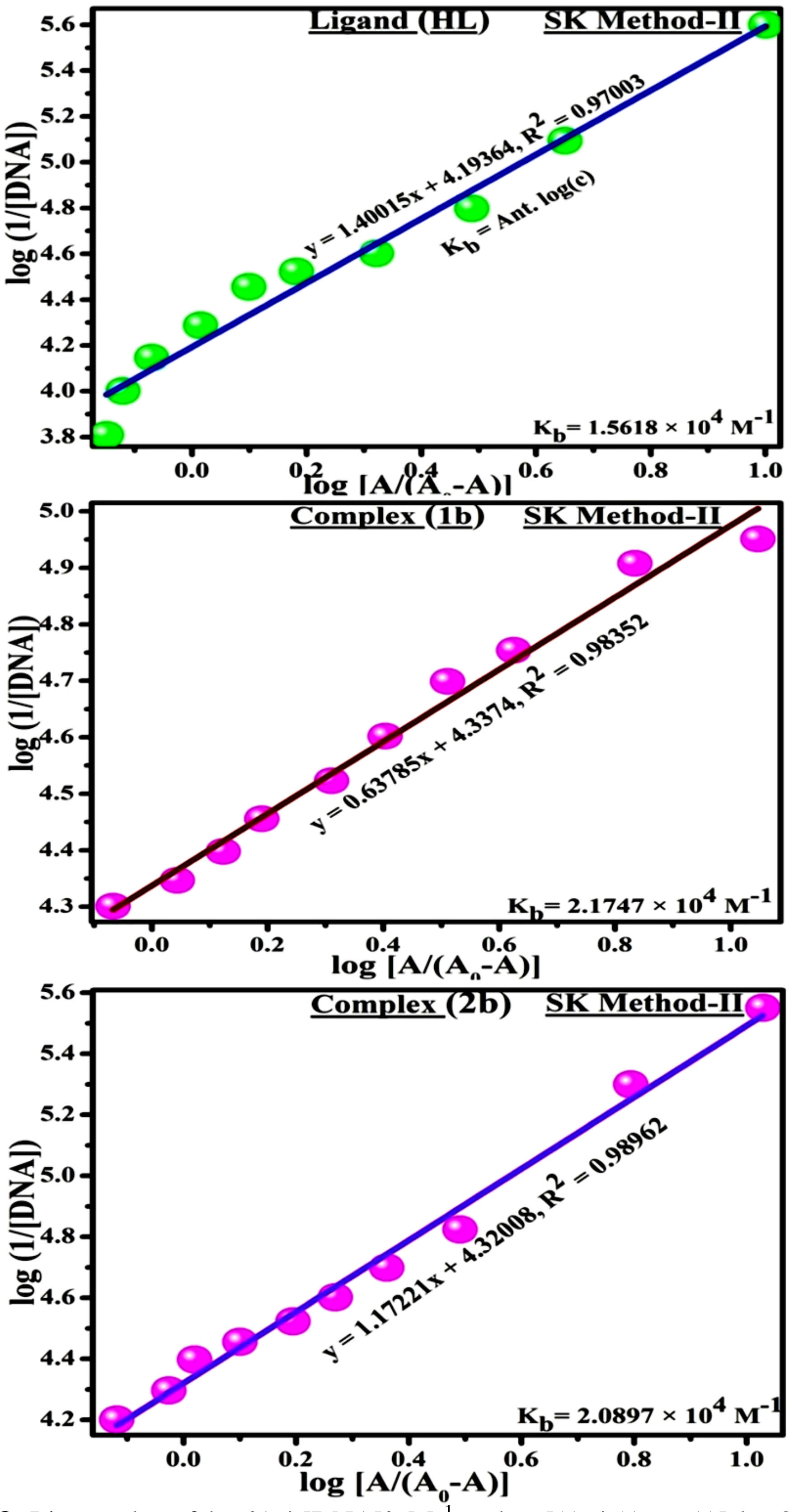

Fig.S18. Linear plot of log{1 / [DNA]} M^{-1} *vs* log [(A / (A_0 – A)] by Sakthi–Kruase-II method for the determination of the intrinsic DNA binding constant (K_b) for ligand (**HL**) and complexes (**1b–2b**).

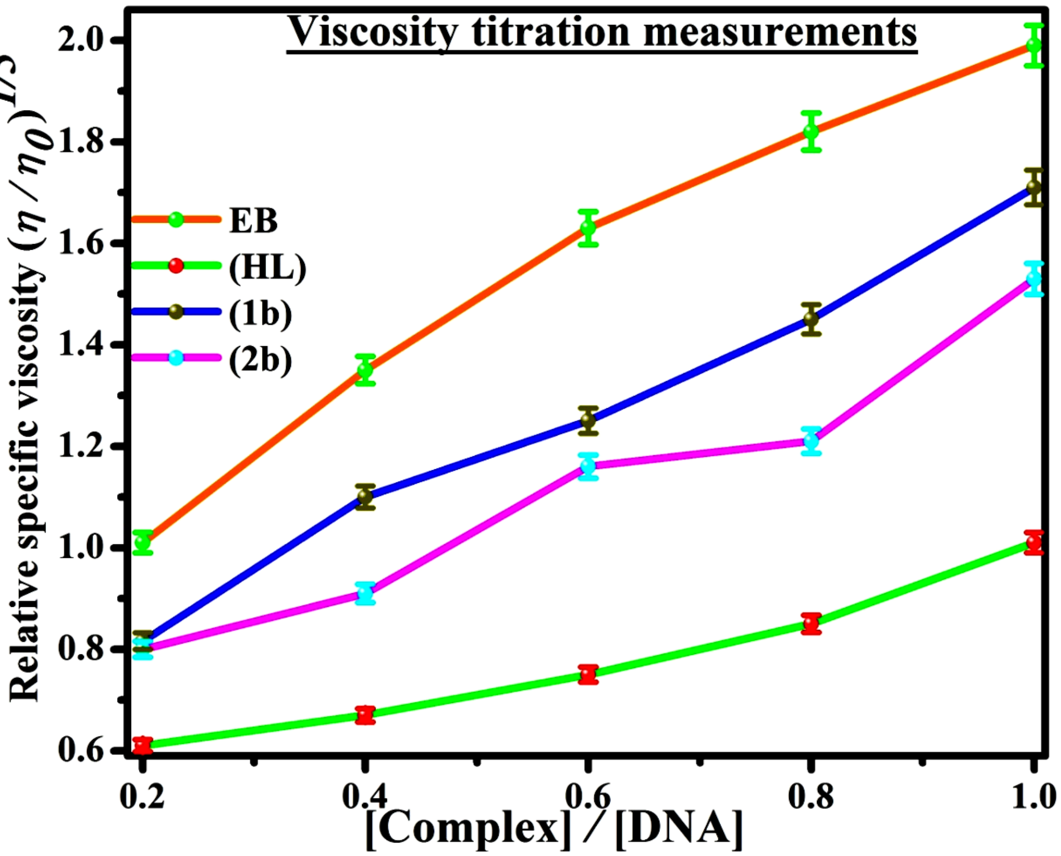

Fig.S19. Relative specific viscosities of CT-DNA in the presence of increasing amounts of ligand (**HL**), mixed ligand complexes (**1b**–**2b**) and Ethidium bromide (**EB**) at 25 °C in 5mM Tris-HCl buffer pH = 7.2 and Error limit ± 2.0 %.

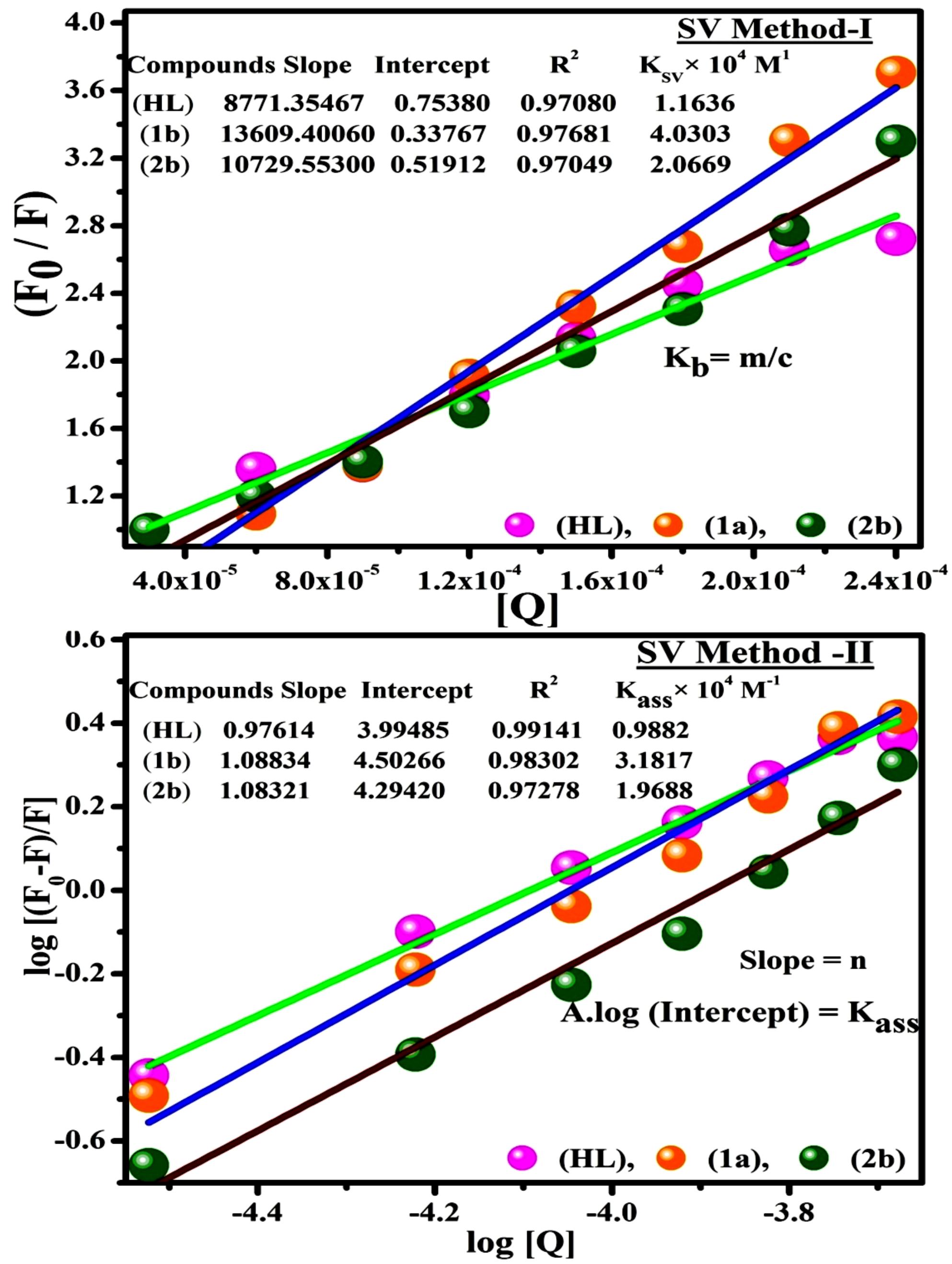

Fig.S20. Stern–Volmer linear plots of F_0 / F *vs* [Q] (Method-I) and log [(F_0-F) / F] *vs* log [Q] (SV Method-II) for the quenching of fluorescence of ethidium bromide (EB)-DNA complex caused by ligand (**HL**) and mixed ligand complexes (**1b**–**2b**).

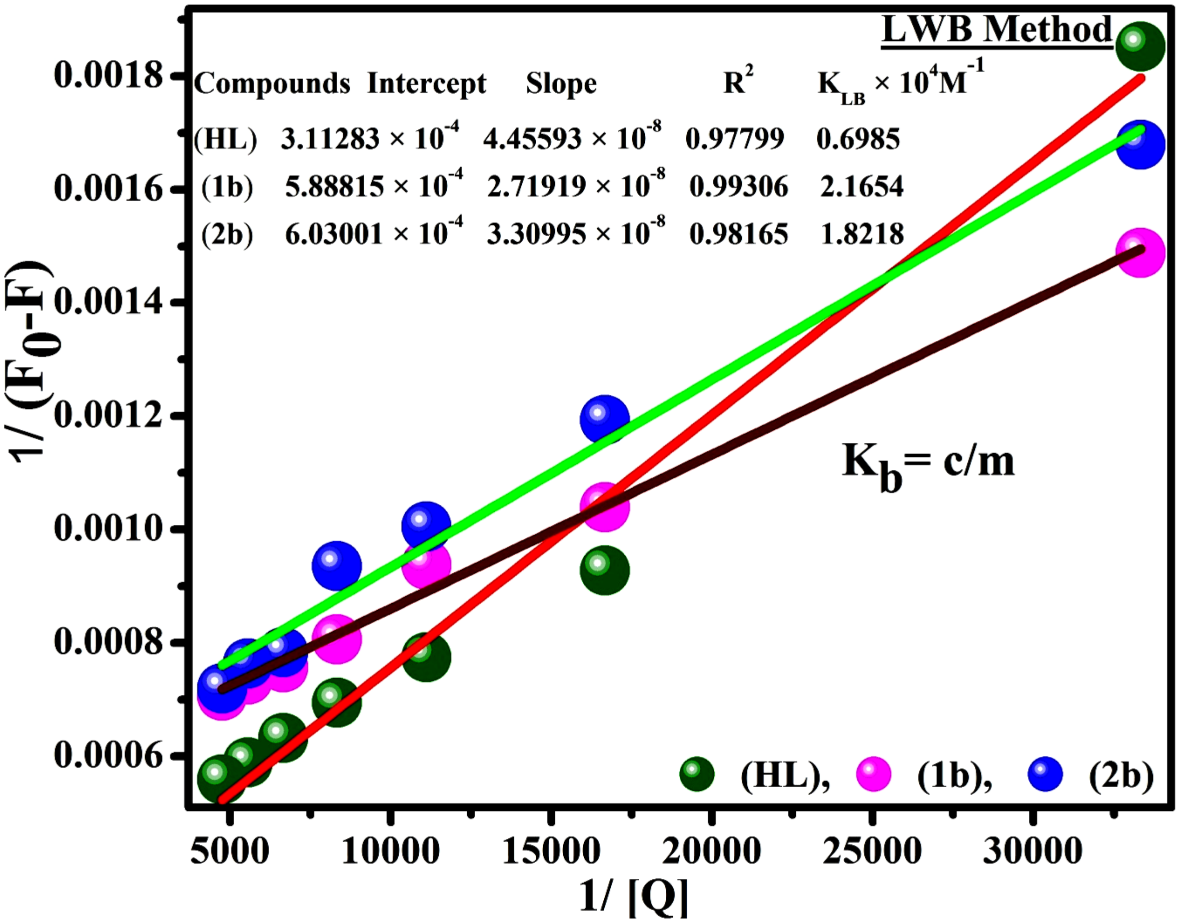

Fig.S21. Lineweaver–Burk linear plot of 1/ (F_0–F) *vs* 1/ [Q] for the quenching of fluorescence of ethidium bromide (EB)-DNA complex caused by ligand (**HL**) and mixed ligand complexes (**1b**–**2b**) (0–240 μM).

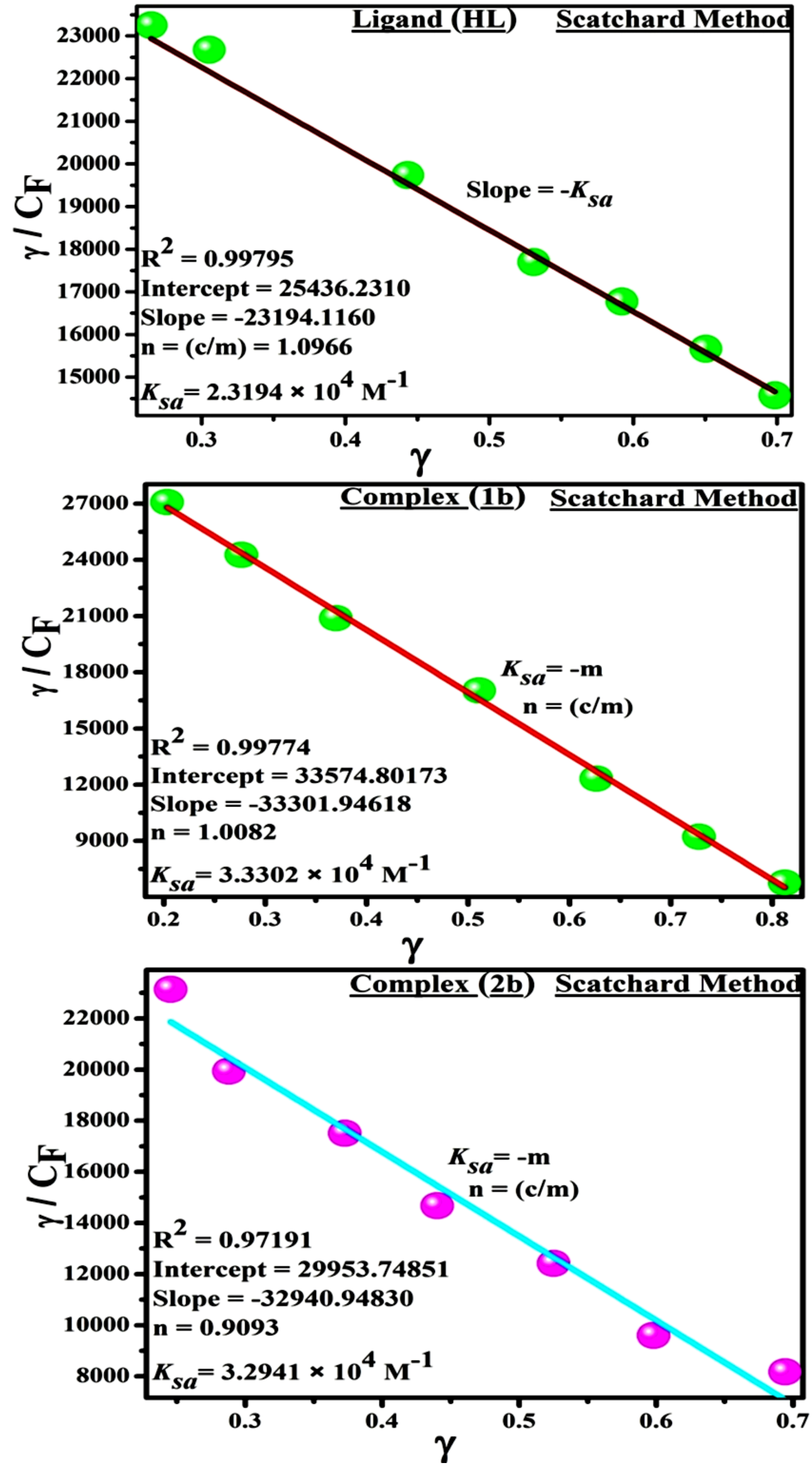

Fig.S22. Determining association binding constant and number of binding sites for ligand (**HL**) and complexes (**1b**–**2b**)–DNA by Scatchard linear plot of (γ / C_F) *vs* γ.

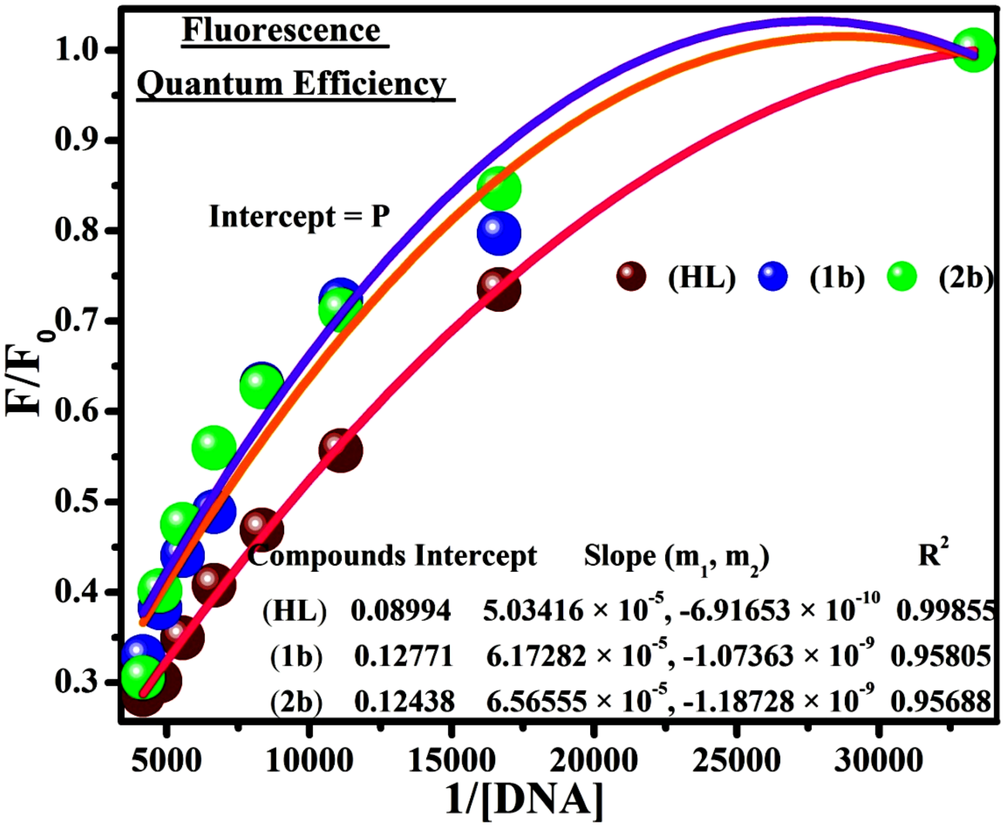

Fig.S23. Determination of ratio of the fluorescence quantum efficiency ($P = \phi_b / \phi_f$) of DNA bound and free compounds [ligand (**HL**) and complexes (**1b–2b**)] from Stern-Volmer linear plots F/F0 *vs* 1/[DNA].

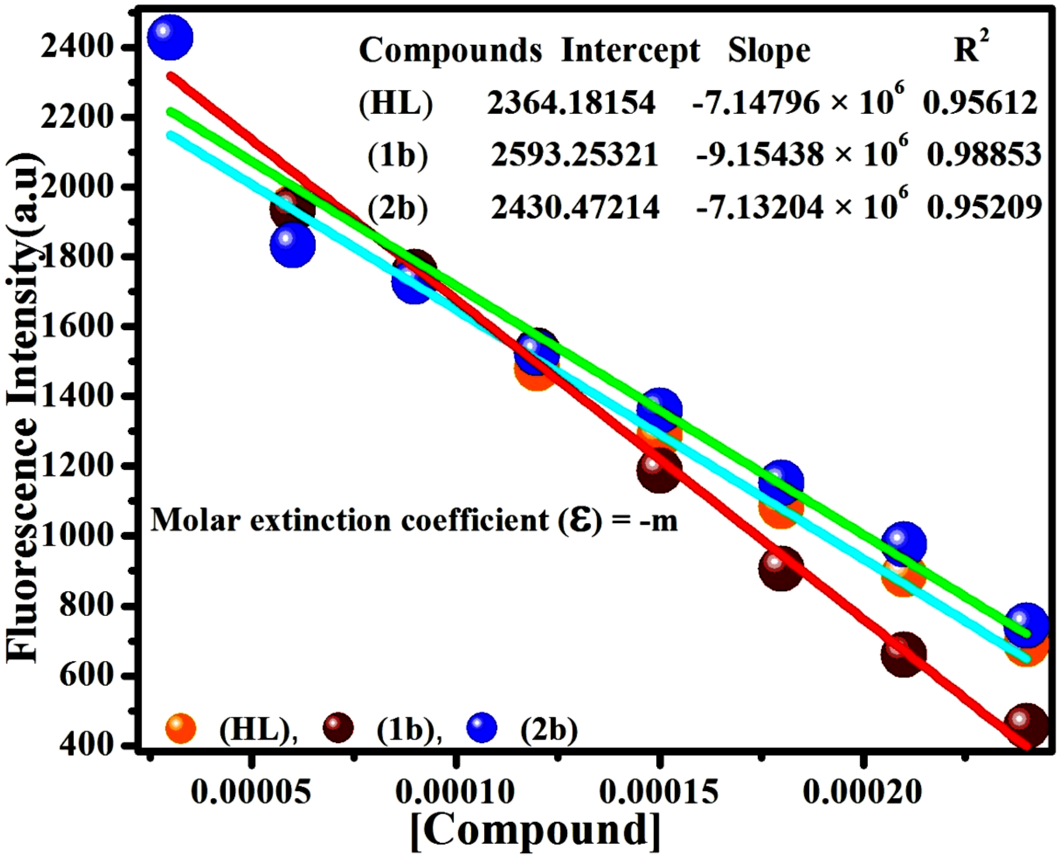

Fig.S24. Determination of apparent DNA binding constant (K_{app}) from the linear plot of fluorescence intensity *vs* concentration of compounds. $K_{app} = K_{EB}\{\frac{[EB]}{[compound]}\}$; Where, $K_{EB} = 10^7\ M^{-1}$ at the concentration of 50 μM EB. IC_{50} value of ligand(**HL**) and complex (**1b**–**2b**) concentration was measured at a 50 % reduction of the fluorescence intensity of EB.

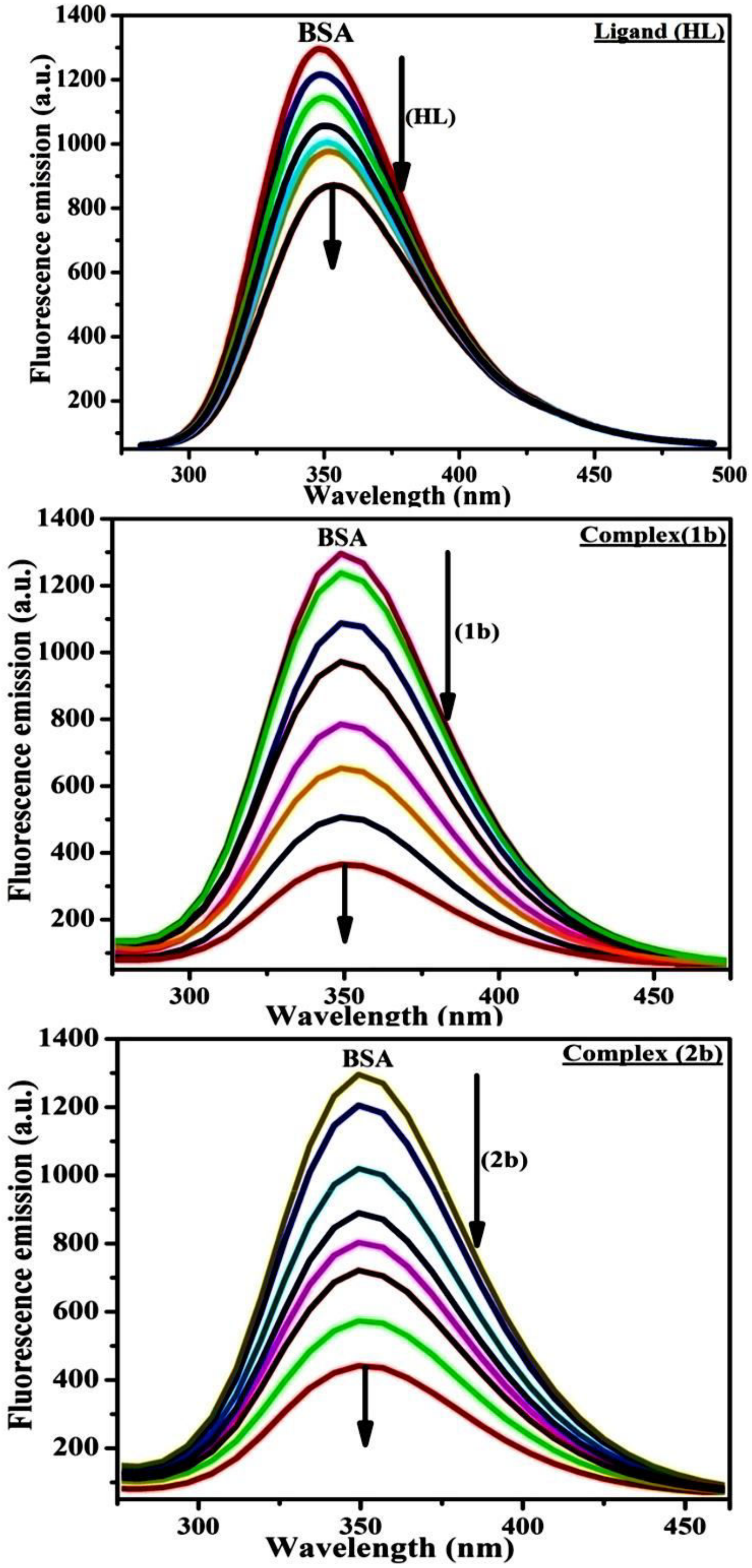

Fig. S25. Fluorescence quenching spectra of BSA (25 µM) in presence of various concentrations of ligand (**HL**) and mixed ligand complex (**1b**–**2b**) (0–25 µM) at 350 nm.

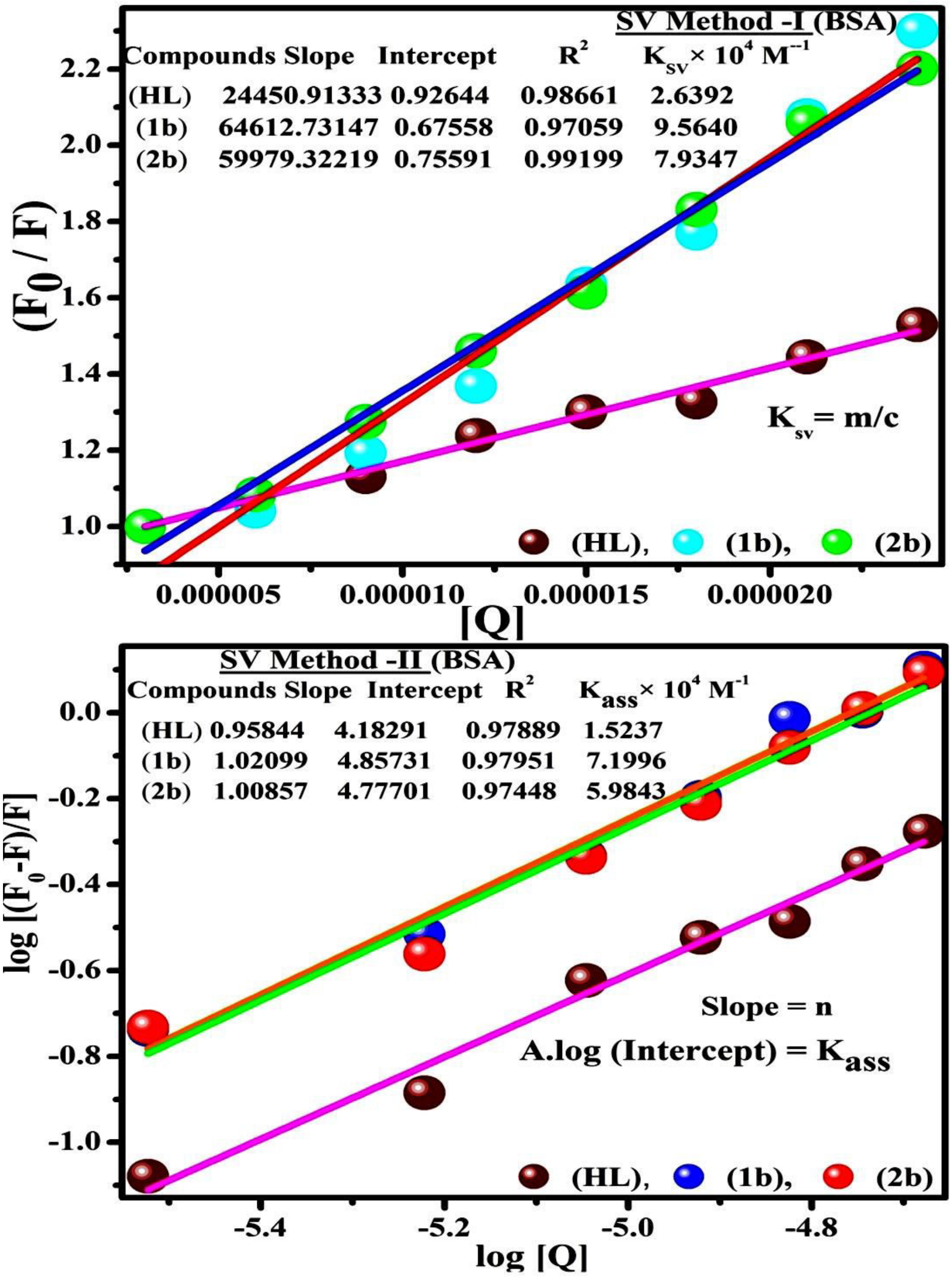

Fig.S26. Stern-Volmer linear plots of F_0 / F *vs* [Q] (Method-I), log [(F_0–F) / F] *vs* log [Q] (SV Method-II) for the fluorescence quenching of **BSA** by free ligand (**HL**) and mixed ligand complexes (**1b**–**2b**).

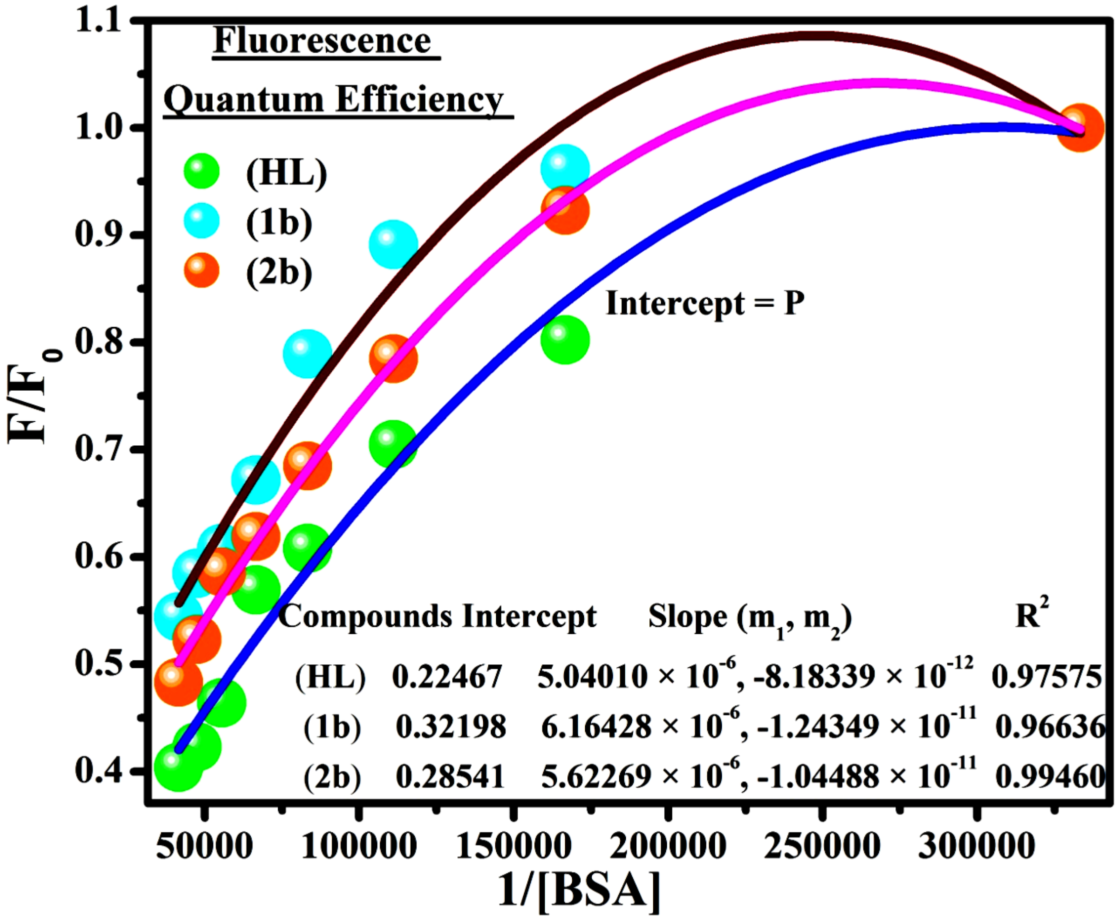

Fig.S27. Determination of ratio of the fluorescence quantum efficiency (P) of BSA bound and free complex (P = ϕ_b / ϕ_f) for ligand (**HL**) and mixed ligand complexes (**1b**–**2b**) from Stern–Volmer linear plots F/F0 *vs* 1/[BSA].

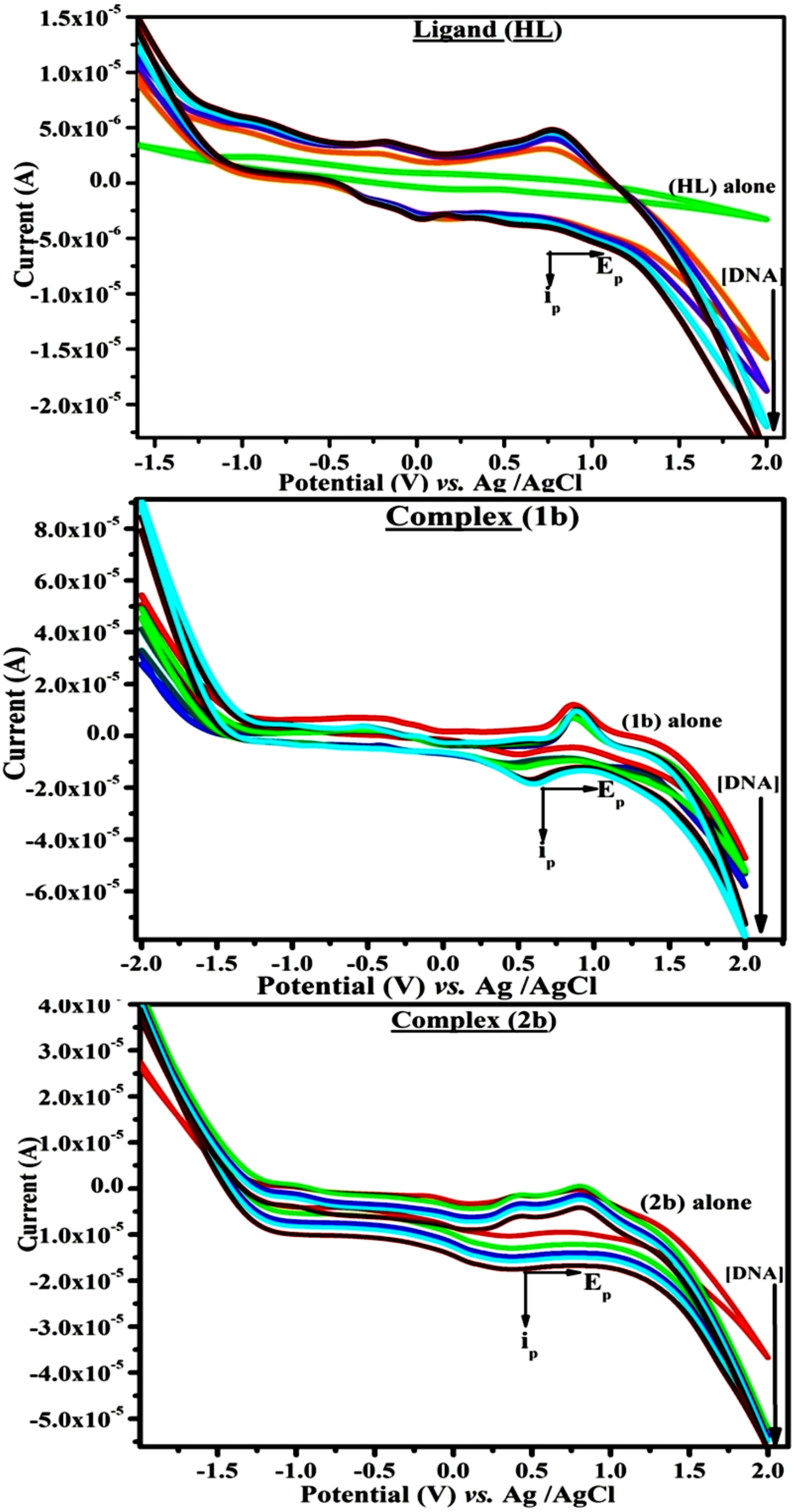

Fig.S28. Cyclic voltammograms of ligand (**HL**) and its mixed ligand complexes (**1b**–**2b**) in 0.05 M Tris-HCl buffer (Supporting electrolyte) pH = 7.2 at 25 °C in presence of increasing concentration of DNA (10 – 50 μM) and arrow indicates the changes in peak current and potentials at scan rate 100 mvs^{-1}.

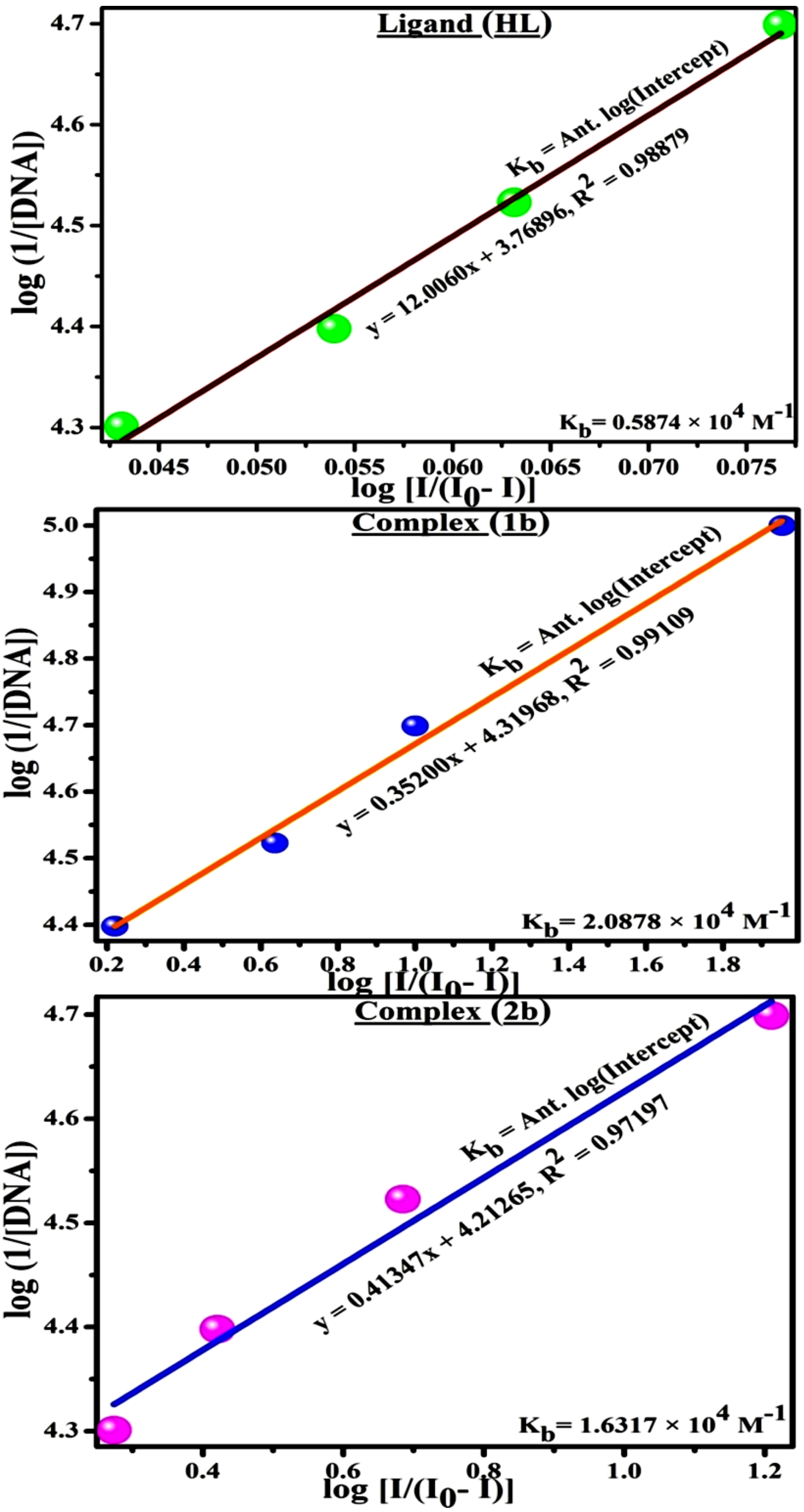

Fig.S29. Plot of log (1/[DNA]) *vs* log (I/(I_0−I)) to evaluate the binding constants for ligand (**HL**) and mixed ligand complexes (**1b**–**2b**) with DNA by Method-I.

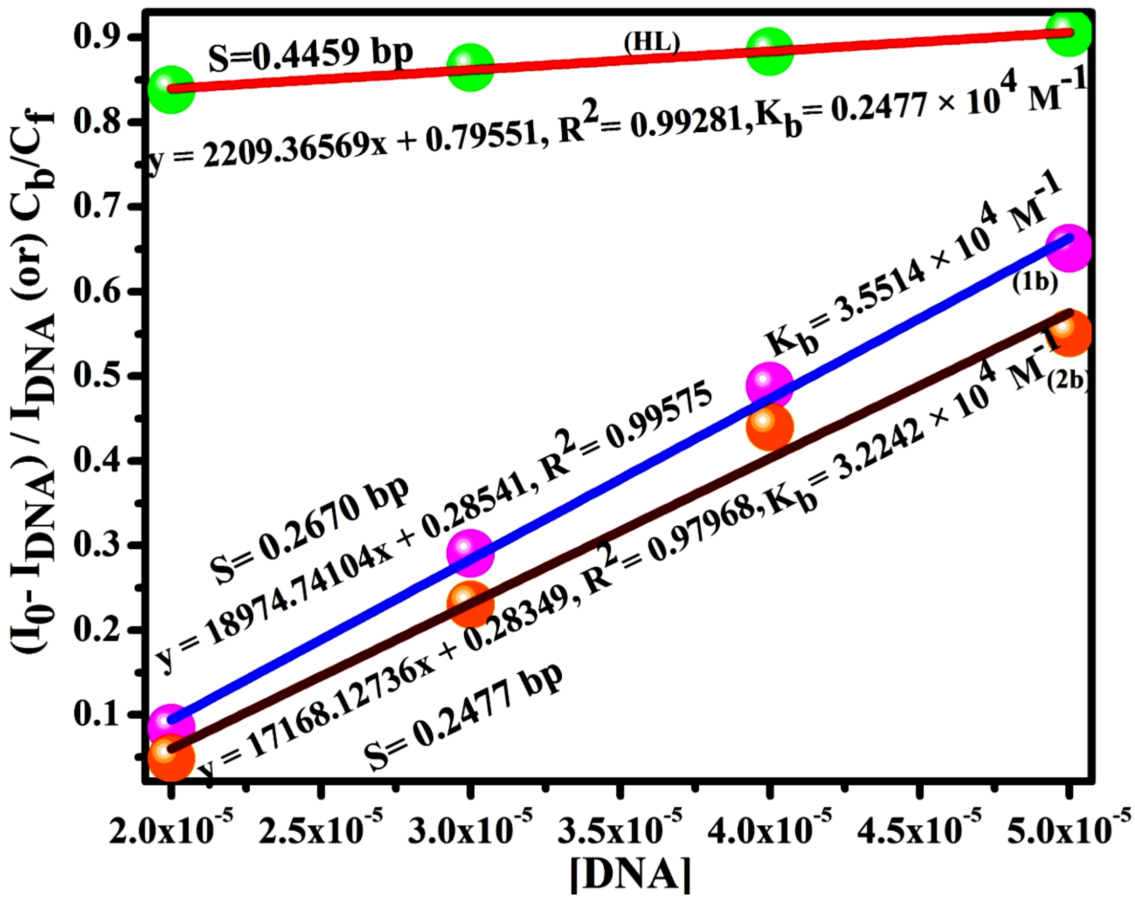

Fig.S30. Plot of C_b/C_f *vs* [DNA] for determination of binding site size (S) and binding constant for ligand (**HL**) and mixed ligand complexes (**1b**–**2b**) with DNA by Method-II.

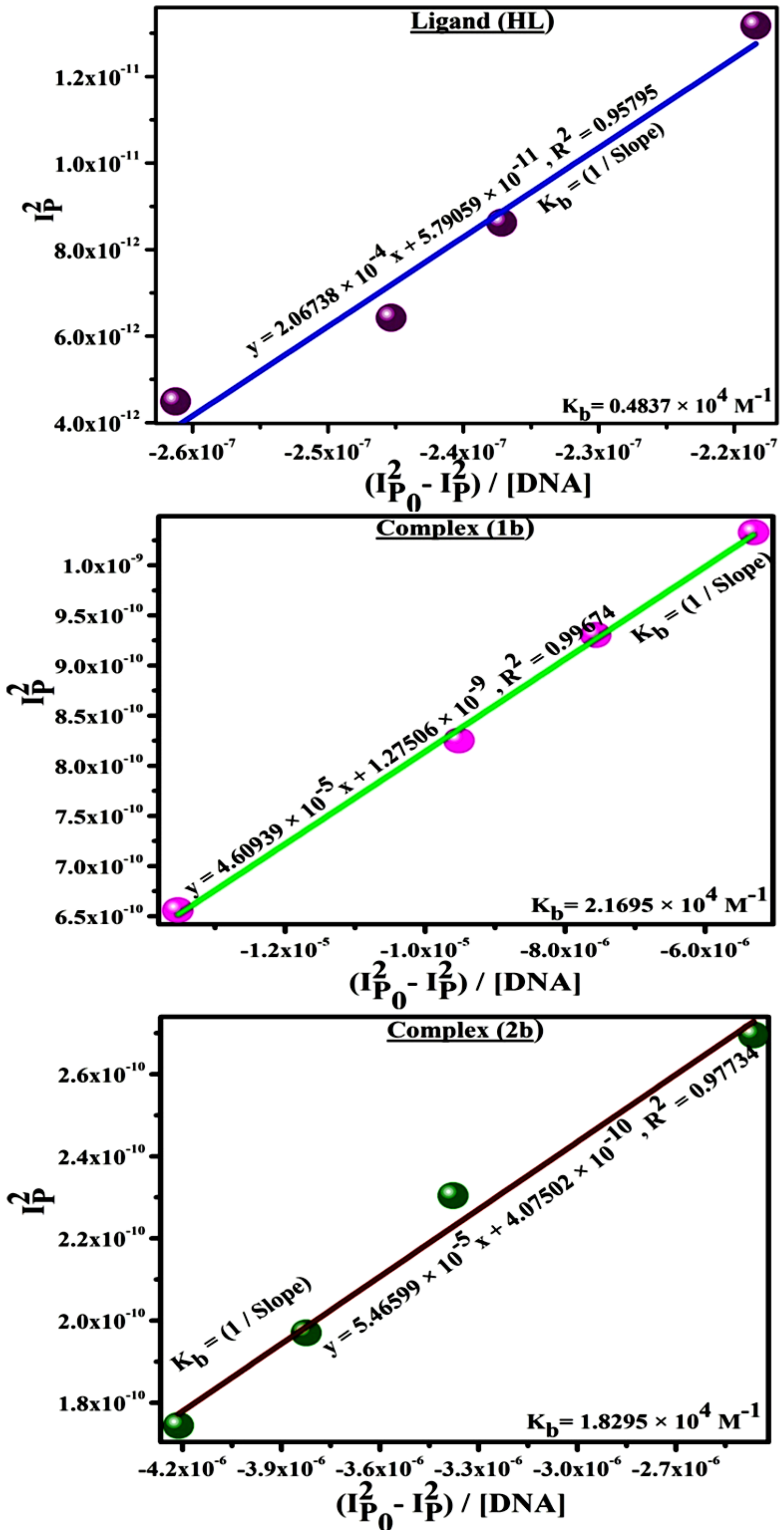

Fig.S31. Plot of Ip^2 *vs* $(I_{po}^2 - I_p^2)$ / [DNA] to evaluate the binding constants for ligand (**HL**) and mixed ligand complexes (**1b**–**2b**) with DNA by Method-III.

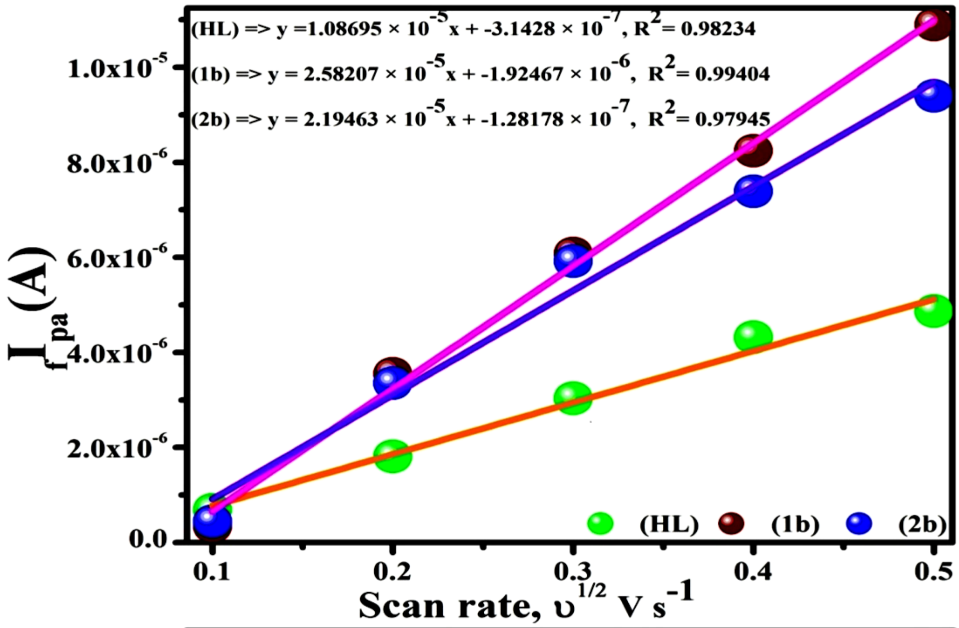

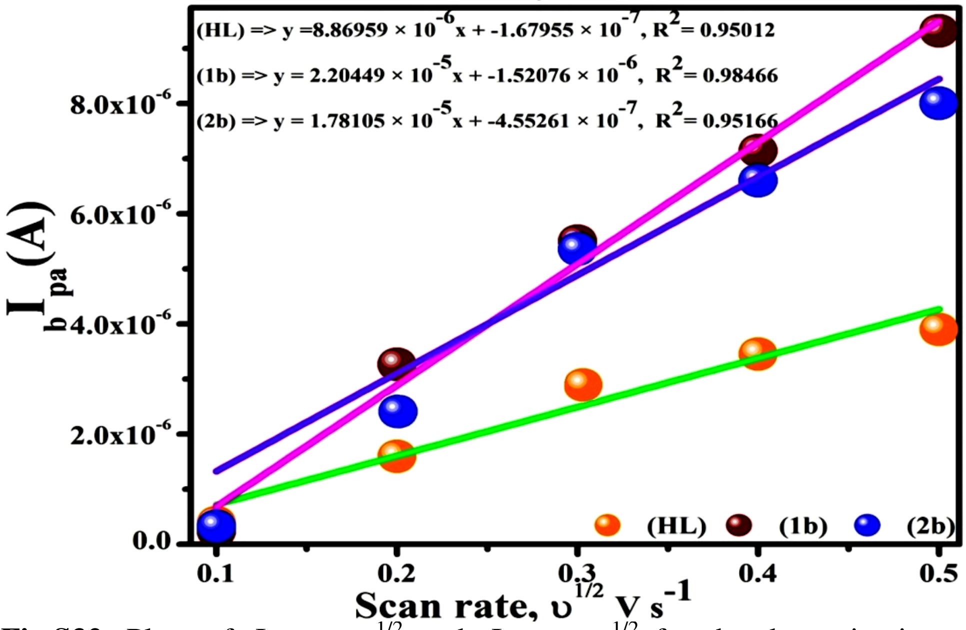

Fig.S32. Plots of $_{f}$Ipa *vs*. $v^{1/2}$ and $_{b}$Ipa *vs*. $v^{1/2}$ for the determination of diffusion coefficients (D_0) of the free compounds in the absence and presence of DNA at scan rates 0.01–0.3 V/s.

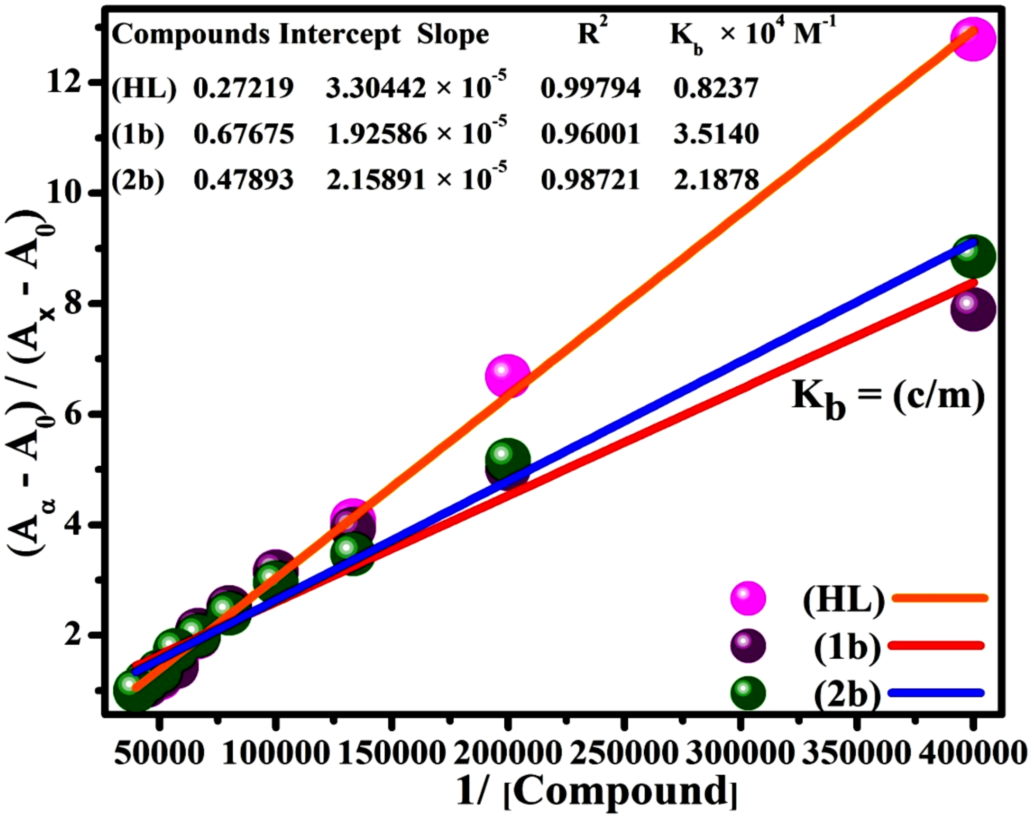

Fig.S33. Benesi–Hildebrand linear plot [(A_∞–A_0) / (A_x–A_0)] *vs* 1 / [compound] determining binding constant for ligand (**HL**) and mixed ligand complexes (**1b-2b**) – BSA at 298 K.

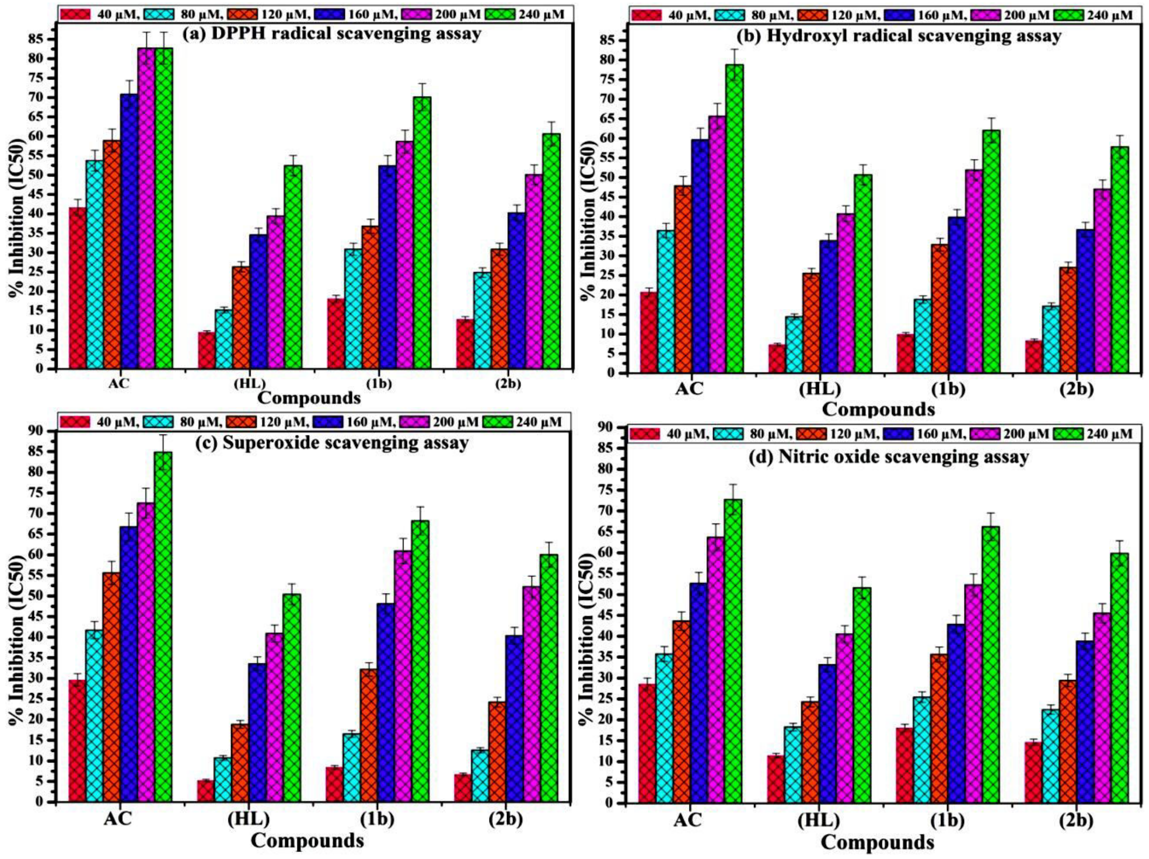

Fig.S34. % inhibition-IC_{50} of of (**a**) DPPH, (**b**) Hydroxyl, (**c**) Superoxide and (**d**) Nitric oxide scavenging assay for ligand (**HL**), mixed ligand complexes (**1b**–**2b**) and standard ascorbic acid (AC-control at various concentration (40, 80,120,160, 200, 240 μM). Error limits ± 2.5 - 5.0.

Supplementary Tables

Table S14 Absorption coefficient (ε) values of ligand (**HL**) and its complexes (**1b**–**2b**) bound to CT-DNA.

Absorption coefficient (ε) ($LM^{-1}cm^{-1}$) & Wavelength (λ_{max}) (nm)	**Apparent absorption coefficient (ε_a) ($LM^{-1}cm^{-1}$) of test compounds.**		
	Ligand (**HL**)	Complex (**1b**)	Complex (2**b**)
**ε_1 (ε_f) (λ_{max})*** for free compound	13385.4545 (**336 nm**)	10281.8182 (**335 nm**)	14230.9091 (**334 nm**)
**ε_2**	13580	10378	14314
**ε_3**	13591.1111	10962.2222	14986.6666
**ε_4**	13890	11427.5	14792.5
**ε_5**	14242.85714	12348.5714	15574.28571
**ε_6**	14803.3333	13510	16966.6666
**ε_7**	16400	15188	19100
**ε_8**	18720	17180	21810
**ε_9**	22553.3333	21506.6666	26673.3333
**ε_{10}**	31740	29710	37970
**ε_{11} (ε_b) (λ_{max})*** for fully binding	62004 (**340 nm**)	52180 (**342 nm**)	67660 (**339 nm**)

$\frac{[DNA]}{(\varepsilon_a-\varepsilon_f)} = \frac{[DNA]}{(\varepsilon_b-\varepsilon_f)} + \frac{1}{K_b(\varepsilon_b-\varepsilon_f)}$ (**1**) → Wolfe-shimmer equation, Where, ε_f → Absorption coefficient of free complex [ε_f = (A/C), where, A→ absorption of free compounds, C→ concentration of free compounds and ε_b → Absorption coefficient of the fully bound to DNA, ε_a is represented as the apparent absorption coefficient value for the MLCT band at a specific concentration of deoxyribonucleic acid and evaluated from Abs/[complex].

Table S15 HOMO-LUMO gaps of ligand (**HL**) and complexes (**1b**–**2b**) calculated in the gas phase and methanol at different DFT levels.

**Compounds**	**Descriptor**	**Gas phase (eV)**			**Methanol (eV)**		
		**B3LYP**	**CAM-B3LYP**	**M06-2X**	**B3LYP**	**CAM-B3LYP**	**M06-2X**
(**HL**)	HOMO(H)	–5.18	–6.56	–6.59	–5.30	–6.68	–7.03
	LUMO(L)	–1.19	–0.08	–0.31	–1.42	–0.16	–0.33
	H-L	3.99	6.64	6.90	3.88	6.84	7.36
(**1b**)	HOMO(H)	–4.16	–5.46	–5.49	–4.80	–6.15	–6.15
	LUMO(L)	–2.27	–1.08	–1.43	–2.68	–0.96	–1.40
	H-L	1.89	4.38	4.06	2.12	5.19	4.75
(**2b**)	HOMO(H)	–4.14	–5.49	–5.42	–4.73	–6.09	–6.12
	LUMO(L)	–1.53	– 0.70	–1.19	–2.02	–0.76	–1.18
	H-L	2.61	4.79	4.23	2.71	5.33	4.94

Table S16 IC_{50} values of DPPH radical scavenging assay at 517 nm.

Compounds	% Inhibition (IC_{50})					
	**40 µM**	**80 µM**	**120 µM**	**160 µM**	**200 µM**	**240 µM**
Ascorbic acid	41.63	53.74	58.95	70.86	82.75	85.65
(**HL**)	09.45	15.21	26.36	34.56	39.41	52.45
(**1b**)	18.11	30.88	36.82	52.42	58.65	70.12
(**2b**)	12.82	24.88	30.89	40.27	50.12	60.66

Scavanging (%) = $\left[\frac{(A_0 - A_S)}{A_0}\right] \times 100$, Where, A_0 → absorbance of the control or (DPPH alone in ethanol) and A_S → absorbance of the sample or (Mixture of DPPH and compounds in ethanol). Error limits ± 2.5 - 5.0.

Table S17 IC_{50} values of Hydroxyl radical scavenging assay at 230 nm.

Compounds	% Inhibition (IC_{50})					
	**40 µM**	**80 µM**	**120 µM**	**160 µM**	**200 µM**	**240 µM**
Ascorbic acid	20.73	36.46	47.87	59.65	65.64	78.83
(**HL**)	07.26	14.43	25.52	33.86	40.73	50.68
(**1b**)	09.94	18.86	32.85	39.84	51.93	62.05
(**2b**)	08.33	17.12	27.05	36.68	47.03	57.83

Scavanging (%) = $\left[\frac{(A_0 - A_S)}{A_0}\right] \times 100$, Where, A_0 → absorbance of the control or (DPPH alone in ethanol) and A_S → absorbance of the sample or (Mixture of DPPH and compounds in ethanol). Error limits ± 2.5 - 5.0.

Table S18 IC_{50} values of Superoxide scavenging assay at 590 nm.

**Compounds**	% Inhibition (IC_{50})					
	**40 µM**	**80 µM**	**120 µM**	**160 µM**	**200 µM**	**240 µM**
Ascorbic acid	29.64	41.72	55.63	66.75	72.55	84.85
(**HL**)	05.23	10.74	18.85	33.55	40.91	50.42
(**1b**)	08.45	16.53	32.18	48.09	60.93	68.21
(**2b**)	06.70	12.58	24.23	40.35	52.23	60.02

Scavanging (%) = $\left[\frac{(A_0 - A_S)}{A_0}\right] \times 100$, Where, A_0 → absorbance of the control or (DPPH alone in ethanol) and A_S → absorbance of the sample or (Mixture of DPPH and compounds in ethanol). Error limits ± 2.5 - 5.0.

Table S19 IC_{50} values of Nitric oxide scavenging assay at 546 nm.

**Compounds**	% Inhibition (IC_{50})					
	**40 µM**	**80 µM**	**120 µM**	**160 µM**	**200 µM**	**240 µM**
Ascorbic acid	28.55	35.75	43.65	52.68	63.72	72.73
(**HL**)	11.42	18.26	24.28	33.21	40.53	51.62
(**1b**)	18.03	25.43	35.67	42.86	52.29	66.21
(**2b**)	14.67	22.43	29.42	38.82	45.53	59.88

Scavanging (%) = $\left[\frac{(A_0 - A_S)}{A_0}\right] \times 100$, Where, A_0 → absorbance of the control or (DPPH alone in ethanol) and A_S → absorbance of the sample or (Mixture of DPPH and compounds in ethanol). Error limits ± 2.5 - 5.0.

……………………………………………………………………………
